# ADAR Regulates RNA Editing, Transcript Stability, and Gene Expression

**DOI:** 10.1016/j.celrep.2013.10.002

**Published:** 2013-10-31

**Authors:** Isabel X. Wang, Elizabeth So, James L. Devlin, Yue Zhao, Ming Wu, Vivian G. Cheung

**Affiliations:** 1 Howard Hughes Medical Institute, Chevy Chase, MD 20815, USA; 2 Department of Genetics, University of Pennsylvania School of Medicine, Philadelphia, PA 19104, USA; 3 Department of Pediatrics, University of Pennsylvania School of Medicine, Philadelphia, PA 19104, USA

## Abstract

Adenosine deaminases acting on RNA (ADARs) convert adenosine to inosine, which is then recognized as guanosine. To study the role of ADAR proteins in RNA editing and gene regulation, we sequenced and compared the DNA and RNA of human B cells. Then, we followed up the findings experimentally with siRNA knockdown and RNA and protein immunoprecipitations. The results uncovered over 60,000 A-to-G editing sites and several thousand genes whose expression levels are influenced by ADARs. Of these ADAR targets, 90% were identified. Our results also reveal that ADAR regulates transcript stability and gene expression through interaction with HuR (ELAVL1). These findings extend the role of ADAR and show that it cooperates with other RNA-processing proteins to regulate the sequence and expression of transcripts in human cells.

## INTRODUCTION

Molecular studies and, more recently, genome and transcriptome sequencing have uncovered the complexity of RNA processing. From the same DNA templates, events such as RNA editing generate different forms of transcripts. In this study, we focused on ADAR-mediated RNA editing and its interactions with other RNA processing steps to regulate gene expression. In human cells, two classes of proteins are known to be involved in RNA editing: the ADAR and APOBEC families. ADARs, which are expressed in a wide variety of cell types, deaminate adenosine to inosine, which is then recognized by the translation and splicing machineries as guanosine (Bass and Weintraub, 1988; Kim et al., 1994; Rueter et al., 1995; Yang et al., 1995). APOBEC1 is expressed predominantly in human liver and converts cytidine to uridine (C-to-U) (Chen et al., 1987; Powell et al., 1987). There are only a few characterized targets of human APOBEC1, the *APOB* and *NF1* genes.

Recent work has uncovered many more RNA-editing events mediated by ADAR proteins. These findings led to new questions. Most of the A-to-G editing sites were identified by computational analysis of sequence data without experimental validation. Some of the findings were based on a comparison of RNA sequences with reference DNA sequences that were not derived from the same cells. In addition, it has been suggested that ADAR plays a role in other biological processes in an editing-independent manner (Clerzius et al., 2009; Heale et al., 2009), but the extent of these processes is not known. Lastly, it is not clear whether ADAR1 and ADAR2 play the same role or different roles in human cells. To address these issues, we sought to answer three main questions: (1) What sites do ADAR proteins edit? (2) Do ADAR proteins regulate gene expression, and if so, is this regulation dependent on editing? (3) What other proteins interact with ADARs in RNA processing?

We compared DNA and RNA sequences in human B cells from two individuals to identify RNA-DNA sequence differences (RDDs). We validated the findings by RNAi and RNA immunoprecipitation (RNA-IP). The results uncovered ~10,000 known and ~50,000 unknown ADAR-mediated A-to-G editing sites in premature and mature mRNAs and long noncoding RNAs (lncRNAs). We also found that ADAR proteins have an editing-independent effect on gene expression. Our results showed that ADAR1 interacts with HuR (ELAVL1) to regulate transcript stability. Together, these results provided us with a deeper understanding of ADAR proteins in RNA editing and gene regulation.

## RESULTS

### DNA and RNA Sequencing

**Figure 1:**
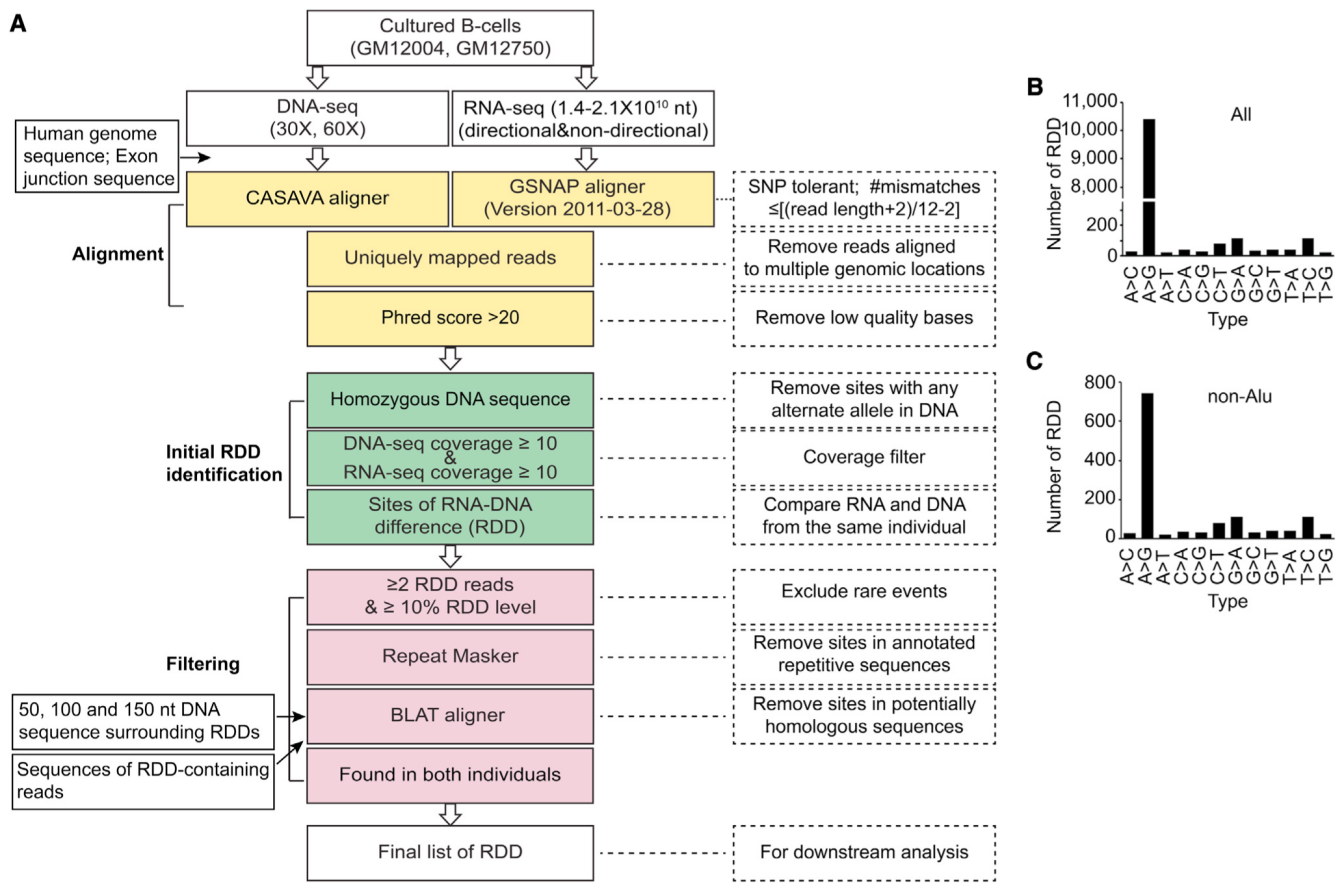
Identification of RDDs (A) Analysis steps to identify RDDs (see also the Supplemental Experimental Procedures). All 12 types of RDDs were found. (B) Sites detected genome wide. (C) Sites detected in non-Alu regions. See also Tables S1 and S2.

**Figure 2:**
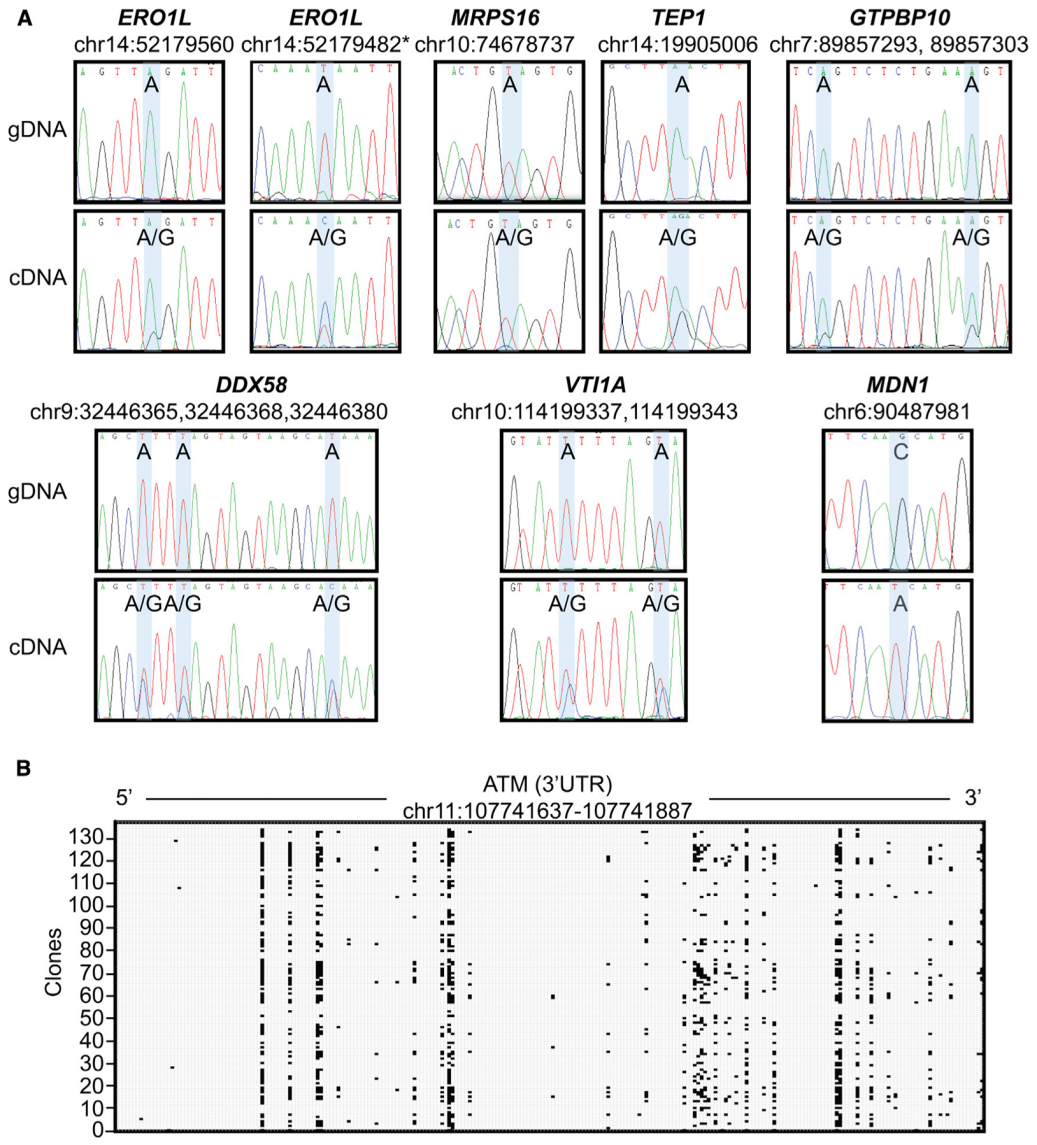
Validation of A-to-G Editing and RDD Sites by Sanger Sequencing (A) Sequences surrounding editing or RDD sites were amplified by PCR using genomic DNA or cDNA from the same two individuals as templates. The sites validated by Sanger sequencing are highlighted in blue and the corresponding nucleotide changes are labeled. Some samples were sequenced from the reverse strand, and the nucleotides are labeled according to the forward strand. *An example of an editing site in *ERO1L* that did not meet our inclusion criteria but nonetheless was validated by Sanger sequencing. (B) Hyperedited region in *ATM* transcript. 3′ UTR of *ATM* was PCR amplified from cDNA and cloned. Sequences from 137 individual clones are illustrated. Each black dot represents an A-to-G site detected in a clone by Sanger sequencing. See also Figure S1.

We sequenced the DNA and mRNA from cultured B cells of two individuals using Illumina-based next-generation sequencing (NGS) (Bentley et al., 2008). We conducted DNA sequencing (DNA-seq) to >30× coverage and obtained >140 million RNA sequencing (RNA-seq) reads for each sample. At least 80% of the sequence reads mapped to the reference genome sequence (Table S1). For each individual, we compared their DNA and mRNA sequences to identify editing and other types of RDDs (Chen et al., 2012; Ju et al., 2011; Li et al., 2011). Data from strand-specific (directional) sequencing allowed us to annotate all 12 types of possible mismatches between DNA and RNA sequences. To simplify the mapping of the sequence reads, repetitive sequences are often excluded. However, since most of the ADAR-mediated A-to-G editing sites were found in Alu repeats (Athanasiadis et al., 2004; Kim et al., 2004; Levanon et al., 2004; Peng et al., 2012), we retained Alu sequences (but excluded other sequence repeats) in our analysis. Using stringent thresholds, we identified 10,992 sites where the RNA sequences were discordant from the corresponding DNA sequences in both individuals (Figure 1A; Table S2). All 12 types of RDDs (A-to-C, A-to-G, etc.) were found (Figure 1B). These included 9,675 sites in Alu-containing regions and 1,317 sites in nonrepetitive regions of the genome. The distributions of the 12 types of RDDs were very different for Alu-containing and Alu-free regions of the genome. Most (99%) of the sites in Alu regions were A-to-G editing sites, whereas in regions without Alu repeats, only 57% were A-to-G sites (Figure 1C). We then validated the results by Sanger sequencing and emulsion-based droplet digital PCR (Figures 2 and S1). Twenty-four out of 25 sites were validated by Sanger sequencing, and five out of six sites were validated by droplet digital PCR. Thus, the false discovery rate (FDR) is approximately 6.5% (Figure S1).

### ADAR1 Plays a Major Role in A-to-G RNA Editing in Human B Cells

**Figure 3:**
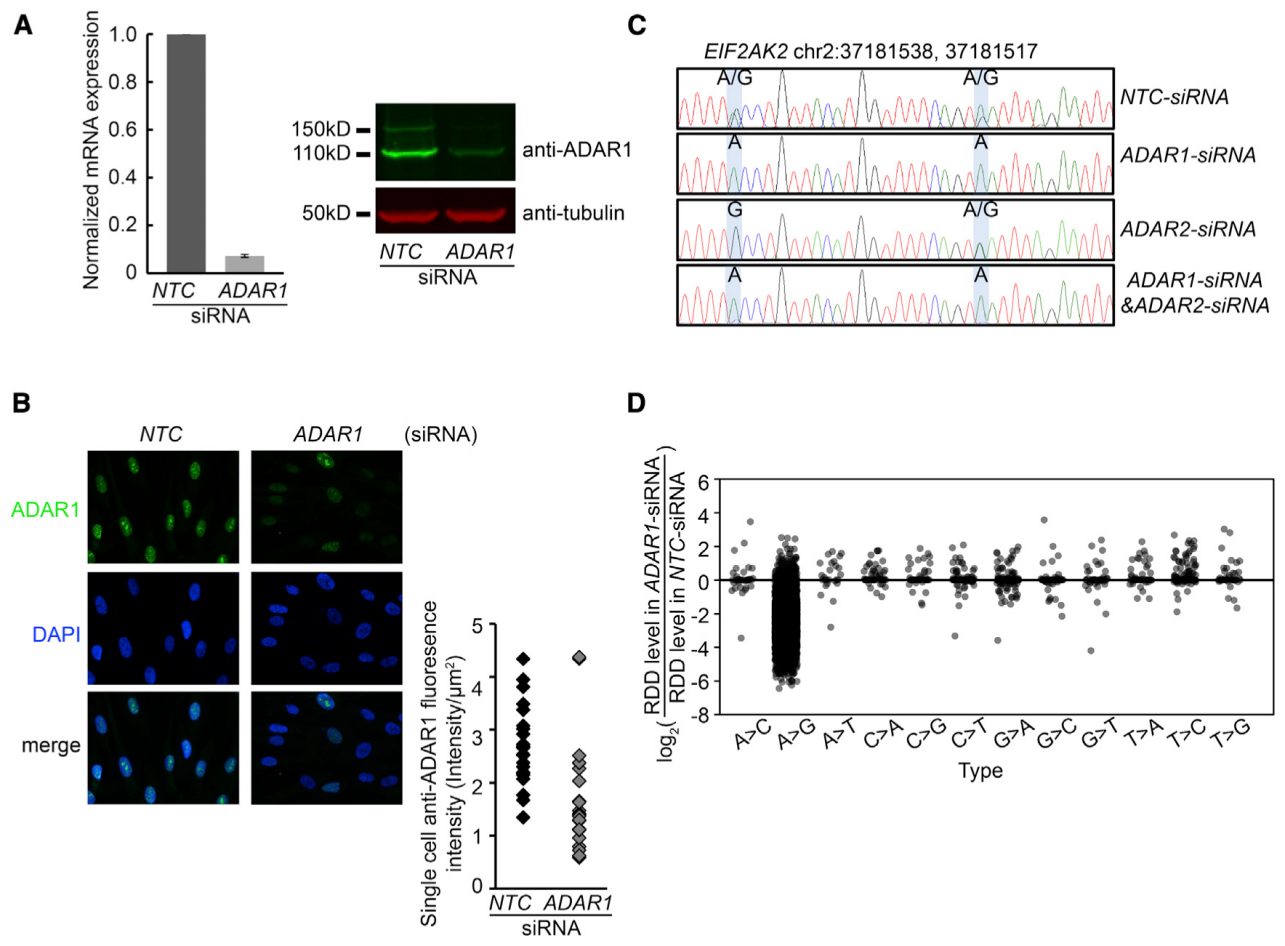
siRNA Knockdown of *ADAR1* Resulted in Reduced A-to-G Levels (A) Left panel: real-time RT-PCR shows the decrease in the *ADAR1* mRNA level following knockdown using pooled siRNA. The average fold change from triplicates is shown. Error bar indicates SEM. Right panel: western blot shows the decrease of ADAR1 protein following knockdown. (B) Immunofluorescence staining of primary fibroblast confirmed that siRNA knockdown results in a decrease of ADAR1 expression. Left panel: representative immunofluorescence image of primary fibroblasts treated with nontargeting control siRNA (NTC) or *ADAR1*-siRNA. Right panel: fluorescence quantification of ADAR1 expression in 24 cells treated with NTC-siRNA or *ADAR1*-siRNA, respectively. (C) Editing levels at two A-to-G sites in *EIF2AK2* were reduced following *ADAR1* knockdown, but the levels increased following *ADAR2* knockdown and were abolished following double knockdown. (D) *ADAR1* knockdown led to reduced levels in 96% A-to-G sites, but had a minimal effect on other types of RDDs. See also Figures S2–S4 and Tables S3, S6, and S7.

To assess the extent to which the ADAR family of deaminases contributes to mismatches between RNA and corresponding DNA sequences, we carried out RNAi-mediated gene knockdowns and deep sequencing of the resulting cells. Human B cells possess three members of the ADAR family: ADAR1, ADAR2, and ADAR3. ADAR1 and ADAR2 are functional deaminases (Bass and Weintraub, 1988; Kim et al., 1994), whereas ADAR3 does not have a known enzymatic function (Chen et al., 2000). The expression level of *ADAR1* is >20 times higher than that of *ADAR2* and *ADAR3* (reads per kilobase of transcript per million mapped reads [RPKM] of *ADAR1* = 7 compared with RPKM of *ADAR2* and *ADAR3* < 0.3), suggesting that ADAR1 is the predominant form of ADARs in human B cells. Following gene knockdown with four independent siRNAs and a pool comprising the four siRNAs, *ADAR1* was reduced by >50% at mRNA and protein levels (Figures 3A, 3B, S2A, and S2B). The editing activities were also reduced, as A-to-G editing in *EIF2AK2* mRNA, a known target of ADAR1 (Blow et al., 2004), was abolished following *ADAR1* knockdown (Figures 3C and S2C). Similar results were obtained from the different siRNAs; for subsequent experiments, we used the pooled siRNAs to minimize off-target effects (Supplemental Experimental Procedures; Figure S2; Grimson et al., 2007).

Next, we sequenced and compared the DNA and RNA of the siRNA-treated B cells. This allowed us to experimentally validate the editing sites and determine the effect of ADAR1 on editing. False-positive results due to misalignment of sequence reads or other artifacts would not “respond” to siRNA treatments.

ADAR and RNAi pathways work cooperatively (Scadden and Smith, 2001; Wu et al., 2011; Yang et al., 2005), so the double-stranded RNAs (dsRNAs) used in gene knockdown likely have effects on ADAR function other than knockdown of its expression level. To study the specific effects of *ADAR1* knockdown, we compared the sequences of cells transfected with control siRNAs with those of cells treated with pooled *ADAR1*-specific siRNAs. In the cells treated with control siRNA, we found 6,996 sites where the RNA and DNA sequences were discordant, including 6,524 A-to-G editing sites. In the *ADAR1* knockdown cells, the editing level of 6,258 (96%) sites decreased by 20% or more in samples from both individuals, whereas only 43 sites of the other 11 types of RDDs decreased by the same extent (Figure 3D; Table S3). A small number of sites (91 of the A-to-G sites and 125 of the other RDDs) showed increased levels following *ADAR1* knockdown. The editing levels of >2,000 A-to-G sites were reduced to zero following *ADAR1* knockdown. These included sites in genes that encode caspases (*CASP8* and *CASP10*) and the von Hippel-Lindau (*VHL*) tumor suppressor, which have been implicated in various cancers. In contrast, the levels of the other types of RDDs did not change or decreased very modestly. This suggests that ADAR1 mediates the majority of A-to-G editing in B cells and does not contribute to the other types of RDDs. In addition, these results show that the FDR of A-to-G editing is no more than 4%.

The above data were obtained at one time point. In order to study the kinetics of A-to-G editing, we carried out RNA-seq on the cells at several time points after siRNA transfection. The expression level of *ADAR1* and the editing levels of its many targets remained low throughout the time course (Figures S3A and S3B). For instance, the A-to-G editing levels in *TRAF1*, *CENPH*, and *USP46* were less than 5% of those in control samples 96 hr after siRNA transfection. Gene Ontology analysis (Ashburner et al., 2000; Huang et al., 2009a, 2009b) showed that editing targets are enriched for genes that encode zinc-finger proteins (p < 0.05), as well as proteins that are involved in chromosomal organization (p < 10^−5^) and antiviral defense (p < 10^−3^).

### Role of ADAR2 in RNA Editing in Human B Cells

**Figure 4:**
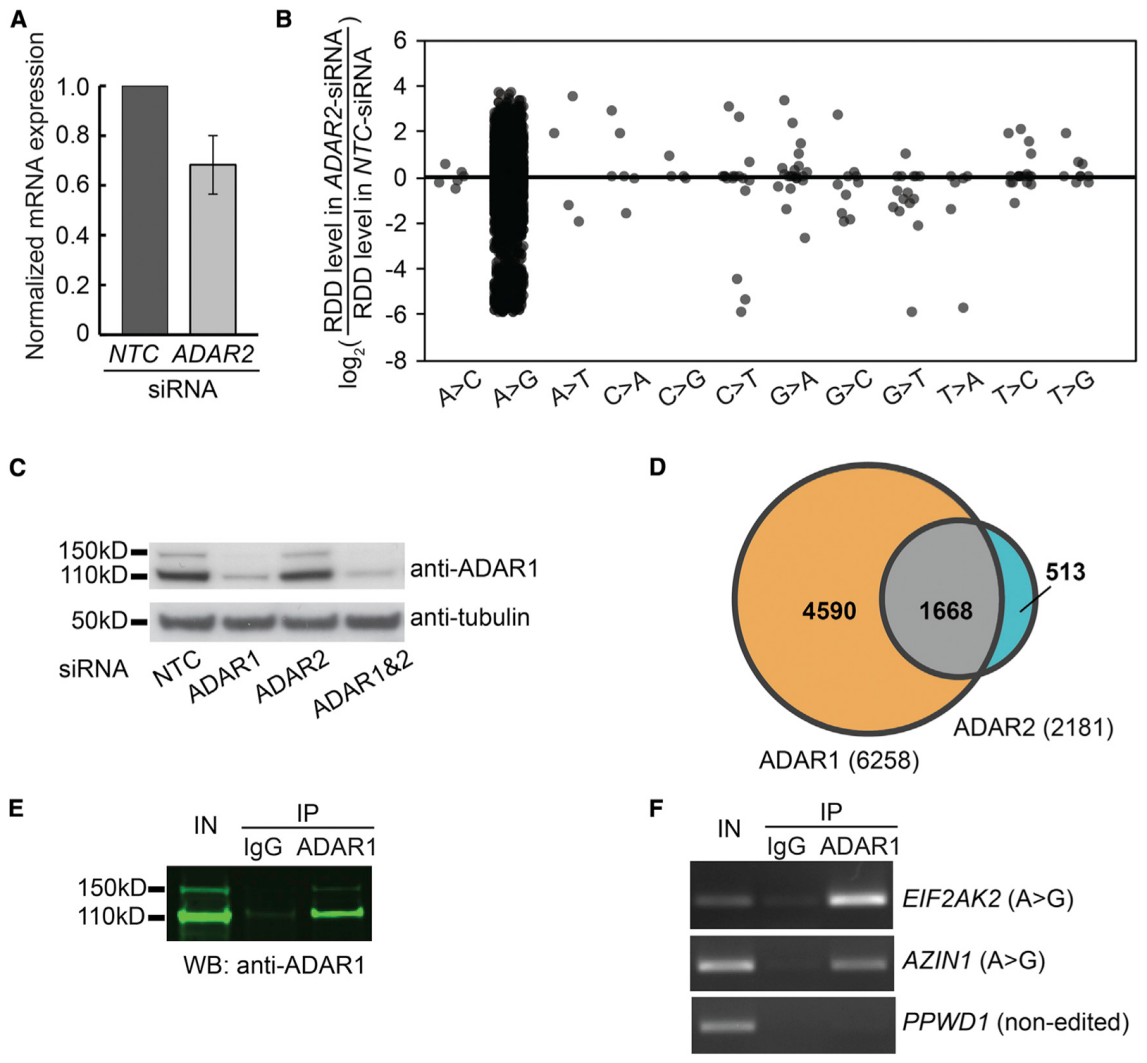
Role of ADAR2 in RNA Editing (A) Real-time RT-PCR shows that the *ADAR2* mRNA level is downregulated following siRNA knockdown. We were unable to assess changes in the ADAR2 protein level because none of the antibodies we tested gave a specific ADAR2 signal in western blot. Error bar indicates SEM. (B) *ADAR2* knockdown led to changes of editing levels in ~2,000 A-to-G sites. See also Table S4. (C) Western blot shows that the ADAR1 protein level is not upregulated following *ADAR2* knockdown. (D) ADAR1 targets more editing sites than ADAR2. The Venn diagram shows shared and unique editing sites targeted by ADAR1 and ADAR2. (E) Anti-ADAR1 RNA-IP pulled down ADAR1 protein and its associated editing targets specifically. Western blot shows that anti-ADAR1 pulled down ADAR1 protein. (F) RT-PCR shows that ADAR1 antibody pulled down transcripts of the editing targets, *EIF2AK2* and *AZIN1*, but not the negative control transcript, *PPWD1*. See also Figure S2 and Table S5.

Next, we carried out siRNA knockdown of *ADAR2* (*ADARB1*) followed by nucleic acid sequencing. The *ADAR2* mRNA level was reduced by 25% (Figures 4A and S2A). The lack of specific antibodies prevented us from measuring ADAR2 protein expression. Following *ADAR2* knockdown, we observed a decrease in its activity: the editing levels of 2,181 of 6,084 A-to-G sites (Table S4), and 32 of the other types of RDDs decreased by at least 20%. In contrast to *ADAR1* knockdown, after *ADAR2* knockdown, the levels of 2,240 A-to-G sites increased by 20% or more (Figure 4B). We reasoned that these sites (e.g., those in *EIF2AK2*) are mainly targeted by ADAR1; therefore, following *ADAR2* knockdown, a compensatory increase in ADAR1 binding or activity would lead to higher editing levels, which would be abolished by the simultaneous silencing of *ADAR1* and *ADAR2*. This hypothesis was confirmed by a decrease in EIF2AK2 editing following double knockdown of *ADAR1* and *ADAR2* (Figure 3C). The compensation is not due to higher ADAR1 protein expression, since it increased only minimally following *ADAR2* knockdown (Figure 4C). These results suggest that the increase in editing levels following *ADAR2* knockdown could be due to increased availability of the sites to ADAR1 and/or homodimerization of ADAR1, a more active form of ADAR1 (Chilibeck et al., 2006; Lehmann and Bass, 2000).

### Shared Editing Targets of ADAR1 and ADAR2

Next, we examined the specificity of ADAR1 and ADAR2 by comparing editing sites identified from the knockdown experiments described above. We found that the editing levels of 6,771 sites decreased after at least one of the ADAR proteins was silenced. Of these, 1,668 sites showed a reduction in editing levels by ≥20% following knockdown of *ADAR1* and *ADAR2*, suggesting they are targets of both enzymes (Figure 4D; Tables S3 and S4). These included sites in genes that encode the DNA damage repair protein ERCC4 and the telomerase-associated protein TEP1. Other targets appeared to be specific to ADAR1 or ADAR2: 4,590 sites showed a decrease in levels following only *ADAR1* silencing, and 513 sites showed a decrease only in *ADAR2* knockdown (Figure 4D). The extent of *ADAR2* knockdown is smaller than that of *ADAR1* knockdown, which could account for the more modest decrease in A-to-G editing following *ADAR2* knockdown.

### RNA-IP Uncovered Many Additional A-to-G Editing Sites

ADAR deaminases are RNA-binding proteins that interact directly with their substrates (Klaue et al., 2003). To understand the RNA-binding activity of ADAR1, we carried out native IP of ADAR1 in B cells and sequenced the RNA that coprecipitated with the ADAR1 protein (Figure 4E). Previously, we selected polyadenylated mRNAs for analysis in order to obtain adequate sequence coverage. Here, we targeted the IP to RNAs that are specifically bound to ADAR1 in vivo without selecting for polyadenylated mRNAs. This allowed us to study the effects of ADAR1 on a broader set of RNAs, including immature transcripts whose introns have yet to be spliced out. To test the quality of the ADAR RNA-IP, we showed that known ADAR1 substrates, such as *EIF2AK2* and *AZIN1*, were bound by ADAR1 protein, in contrast to the control transcript *PPWD1*, which is not edited (Figure 4F). We next carried out RNA-seq analysis and identified edited transcripts that were pulled down by ADAR1 antibody but not by negative-control immunoglobulin G (IgG). Using the same thresholds as above, we identified 55,719 A-to-G sites in the two individuals, which is far more than the 10,412 editing sites identified from the mRNA samples of the same individuals (Table S5). Transcripts that are bound and edited by ADAR1 protein include those that encode WEE1, a protein kinase that plays a role in DNA replication, and COPB1, a member of the coatomer protein complex that is involved in trafficking between the Golgi and the endoplasmic reticulum.

Of these 55,719 sites, fewer than 4,500 sites have been previously reported (Bahn et al., 2012; Carmi et al., 2011; Kiran and Baranov, 2010; Li et al., 2009; Peng et al., 2012). The majority (81%) of the sites were found in introns and some were found in lncRNAs, including *LINC00265* and *LINC00476*. The transcripts from the RNA-IP were hyperedited: >30% of the editing sites clustered in 224 transcripts, each of which had >50 A-to-G editing sites (Table 1). More than 97% of the 55,719 editing sites were in Alu repeats that promote dsRNA formation and therefore binding and hyperediting by ADAR proteins (Osenberg et al., 2009). When we examined a hyperedited region of *ATM* more closely, we found that each adenosine was deaminated. However, the editing level at a given site ranged from 1% to 99%, and within a given transcript there was no obvious pattern as to which adenosine was edited (Figure 2B).

### Features Differ between A-to-G Editing Sites and Other Types of RDDs

**Figure 5:**
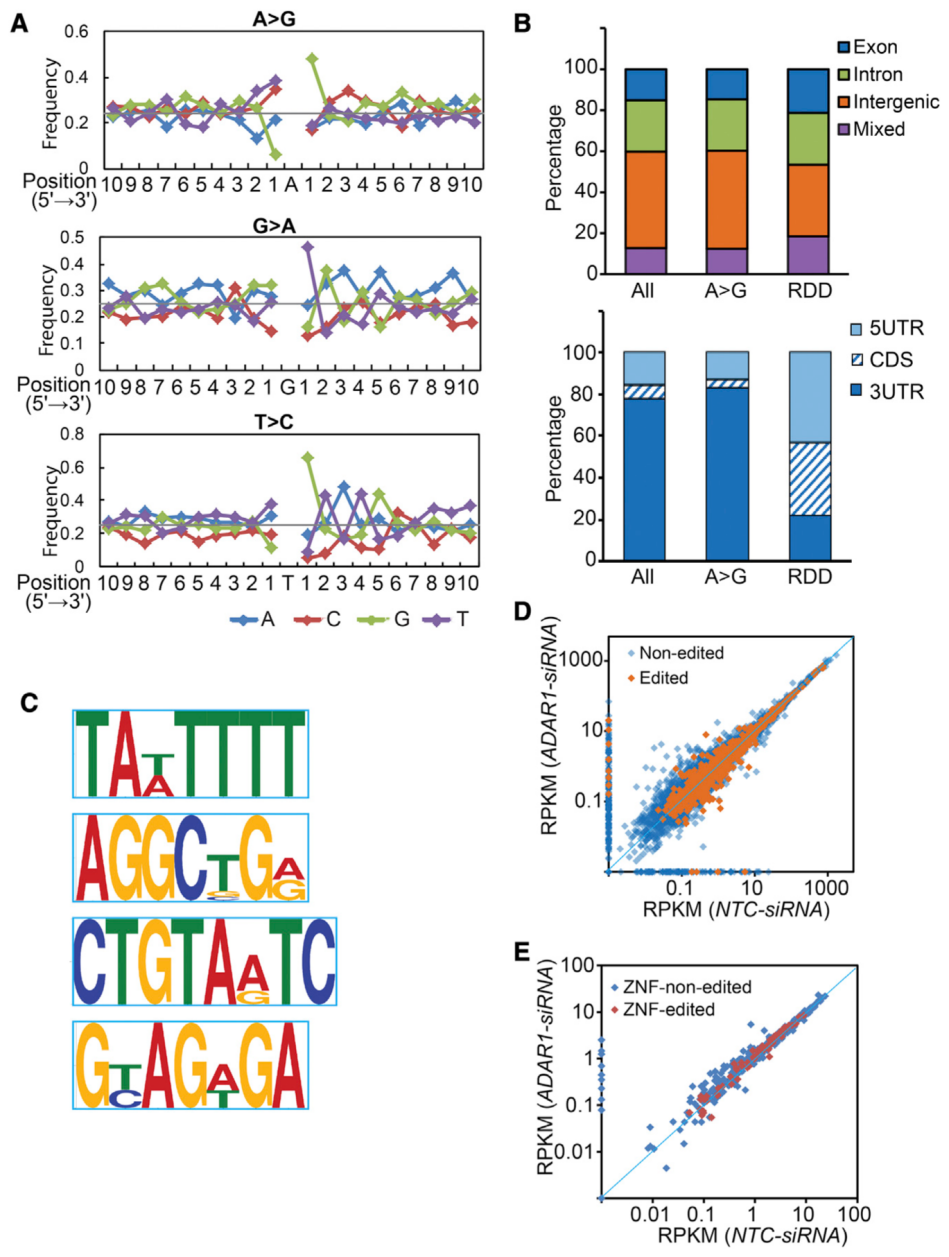
Features of A-to-G and RDD Sites (A) The nucleotide 5′ to A-to-G sites is depleted of G, and the nucleotide 3′ to A-to-G sites is enriched for G. In contrast, the nucleotide 3′ to G-to-A sites is enriched for T, and the nucleotide 3′ to T-to-C sites is enriched for G. Sequences for 10 nt upstream and downstream of A-to-G or RDD sites were analyzed and the frequencies of A, C, T, and G at each position are shown. The horizontal line at a frequency of 0.25 indicates the expected frequency if the four nucleotides are represented equally. (B) A-to-G and other RDD sites are found in different genomic regions. Upper panel: genome-wide distribution (“Mixed” indicates regions with multiple or ambiguous annotation). Lower panel: distribution in exonic regions. (C) Sequence motifs for editing targets pulled down in anti-ADAR RNA-IP assays. The MEME program was used to analyze DNA sequences corresponding to 100 nt upstream and downstream of editing sites. The four motifs that are most significantly enriched in input sequences are shown (p < 10^−10^, Fisher’s exact test). Scrambled sequences were used as negative-control sequences. (D and E) Expression levels of transcripts do not correlate with editing levels. RPKM values of transcripts measured in an *ADAR1* knockdown sample and a negative-control sample (NTC) are plotted. Edited and nonedited transcripts are indicated in different colors. (D) All transcripts. (E) Genes encoding zinc-finger proteins whose expression levels changed by ≥20%. See also Table S8.

The results from *ADAR* knockdown and RNA-IP suggest that although ADARs mediate A-to-G editing, they do not mediate other types of RDDs. The levels of other types of differences were largely unaffected by *ADAR* knockdown, and the transcripts that showed those differences were not bound by ADAR. This prompted us to compare the genomic features surrounding the A-to-G editing sites and other types of RDDs. First, the sequence contexts of A-to-G and non-A-to-G sites are different. The base 5′ adjacent to the adenosine in A-to-G sites is depleted of guanosine (G) and the base 3′ to A-to-G editing sites is enriched for G (Figure 5A), consistent with previous reports (Lehmann and Bass, 2000). This sequence feature is specific to A-to-G editing because it is not present in random adenosines within nonedited Alu repeats (data not shown). This sequence motif was also not found for any of the RDDs. We identified sequence motifs for G-to-A and T-to-C sites, and they differed from the motif around the A-to-G sites (Figure 5A). Second, the A-to-G sites were more clustered than the non-A-to-G sites (67% of A-to-G sites were found within 25 nt of each other, compared with 14% of non-A-to-G RDDs). Third, most of the A-to-G sites were within or near inverted repeats, which form dsRNA and are preferentially recognized and bound by ADAR enzymes. Nearly 45% of the A-to-G sites resided within inverted repeats and another 30% were found near inverted repeats (<1 kb). In contrast, very few (0.9%) of the non-A-to-G sites were found in inverted repeats. Lastly, A-to-G sites and RDD sites were found in different regions of genes. A-to-G sites were found mostly in the 3′ UTRs, whereas RDDs were found mainly in the 5′ UTRs and in coding exons. Only 4% of the A-to-G sites (compared with 35% of RDDs) were in coding exons (Figure 5B). The differences between A-to-G editing sites and the other types of RDDs suggest that they are mediated by different mechanisms. Biochemically, this is expected since some of the RDDs are transversion events that cannot be explained simply by deamination.

### Sequence Motifs near A-to-G Editing Sites

The large number of RNA editing sites in our study gave us an opportunity to uncover characteristics of the editing targets. We expanded our sequence analysis to 100 nt upstream and downstream of A-to-G sites using the motif discovery tool MEME (Bailey et al., 2009). MEME identified four motifs that are significantly enriched in the sequences surrounding the A-to-G editing sites compared with control sequences (p < 10^−10^, Fisher’s exact test; Figure 5C). One of these motifs (TA(T/A)TTTT) corresponds to the binding motif of HuR, an RNA-binding protein that regulates mRNA turnover (Myer et al., 1997). Other studies have also investigated the sequence and structure specificity of targeted sites of ADAR enzymes (Bahn et al., 2012; Daniel et al., 2012; Dawson et al., 2004; Kuttan and Bass, 2012; Lehmann and Bass, 2000; Wong et al., 2001). However, the sequence motifs we described here have not been previously reported in ADAR editing targets. This is likely because we searched more distant sequences surrounding editing targets in a larger number of editing sites of various types of RNAs, whereas most previous studies focused on immediately adjacent sequences on fewer targets.

Finding the HuR motif near ADAR-binding sites led us to reason that ADAR interacts with other RNA-binding proteins. The sequence motifs for RNA-binding proteins in edited transcripts suggest cooperative binding among RNA processing proteins, akin to the coupling seen in regulation of gene expression by multiple transcription factors. This finding prompted us to study the interactions between ADAR1 and HuR proteins (see below).

### ADAR Regulates Gene Expression

After examining how ADAR proteins affect RNA sequences, we turned to study their effects on gene expression and to determine the relationship between RNA editing and gene expression. We found that ADAR1 and ADAR2 affect the expression of thousands of genes and their transcripts in human B cells. We looked for genes that showed changes in the total gene-expression level. Following *ADAR1* knockdown, 635 genes showed significant changes in gene expression in two individuals (p < 0.05; Table S6). The RNA-seq data allowed us to analyze the effect of ADAR on gene expression at single-nucleotide resolution to quantify changes of transcript expression in addition to total gene expression following *ADAR1* knockdown. Many genes demonstrate “isoform switching” under physiological or experimental perturbations (Trapnell et al., 2013). The expression levels of 1,238 transcripts showed significant changes in expression (Table S6). Nearly half of these transcripts (579) belong to the genes that changed the total expression level. However, changes in 659 transcripts were not reflected at the total gene-expression level. For some transcripts, such as *VNN2* and *ARH-GAP19*, two isoforms showed changes in opposite directions, and thus the total gene levels that are the sums of isoforms did not show change (Figure S3C). Gene Ontology analysis (Huang et al., 2009a) showed that these ADAR-regulated genes are enriched in kinase (p < 10^−9^), DNA damage response proteins (p < 10^−10^), and zinc-finger proteins (p < 10^−6^; Table S7).

RNA-seq data provide information on editing and gene expression in the same samples, and thus allow us to assess the connection between the two. We examined the levels of ADAR1-dependent editing and transcript expression, and found that they were not correlated (r < 0.05 for both individuals). Following *ADAR1* knockdown, changes in expression level were independent of the editing status of the target genes (Figure 5D). For example, among the 263 zinc-finger protein genes whose expression levels changed following *ADAR1* knockdown, only 40% (104 genes) were editing targets of ADAR1. ADAR1 regulated the expression of zinc-finger proteins regardless of whether they were editing targets or not (Figure 5E). For instance, the expression levels of *ZNF16* decreased and those of *ZNF432* increased following *ADAR1* knockdown; however, even though they both had multiple Alu repeats, neither gene was edited. Therefore, editing of Alu is not required for ADAR1 to regulate the expression of zinc-finger proteins (Shen et al., 2011).

Another way to investigate the relationship between RNA editing and gene-expression regulation is to study the 106 genes that are both edited and regulated by ADAR1 at the mRNA expression level (Table S8). Among these, following *ADAR1* knockdown, the expression levels of 67 genes increased and those of 39 genes decreased. Changes in editing levels and gene expression following *ADAR1* knockdown were not significantly correlated (r < 0.05). For example, *IKZF3*, a transcription factor that regulates proliferation and differentiation of B lymphocytes, has 68 A-to-G editing sites. Its expression level increased by 1.3-fold, whereas its editing level decreased by >7-fold following *ADAR1* knockdown. In contrast, both the editing and expression levels of *CENPN* (43 A-to-G editing sites) decreased following *ADAR1* knockdown. The positions of edited sites within genes (such as coding exons, 3′ UTRs) and the number of edited sites per transcript also did not correlate with changes in expression following *ADAR1* knockdown. These results further suggest that ADAR1 can affect gene expression independently of its deamination activity.

We also examined the editing and gene-expression regulatory roles of ADAR2. Although ADAR2 has fewer editing targets than ADAR1, it regulates the expression levels of more genes. Following *ADAR2* knockdown, the expression levels of 4,154 transcripts (in 3,379 genes) increased by 2-fold, and those of 872 transcripts (in 734 genes) decreased by 2-fold (Table S9). Thus, ADAR2 has a broader effect on gene expression even though it plays a lesser role in editing compared with ADAR1. This further implies that ADAR proteins affect editing and gene expression independently.

### ADAR1 Interacts with HuR to Regulate Transcript Stability

**Figure 6:**
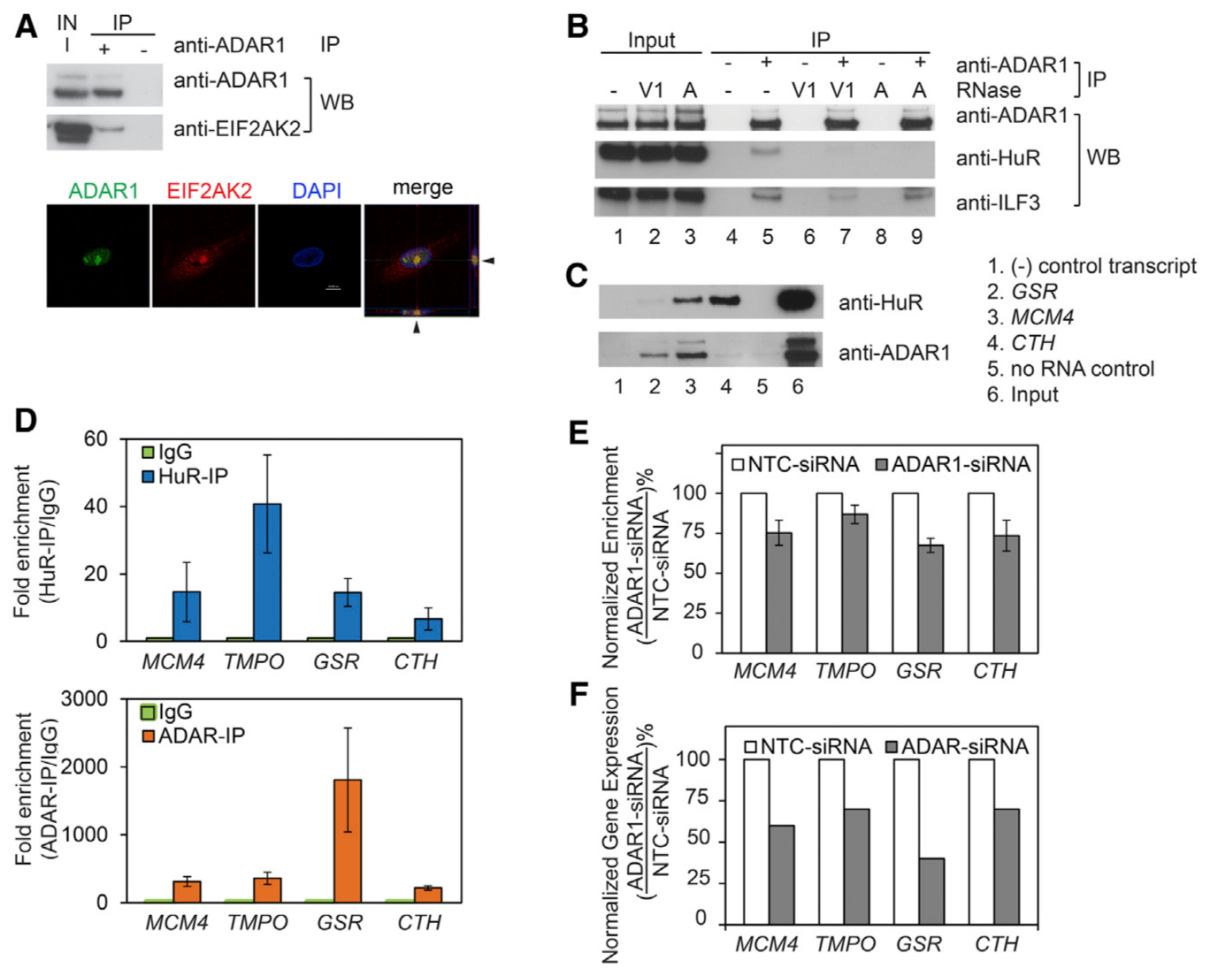
ADAR1 and HuR Proteins Interact in an RNA-Dependent Manner and Coregulate Common Transcripts (A) Anti-ADAR1-IP of ADAR1 and its interacting protein EIF2AK2. Western blot analysis shows that ADAR1 and EIF2AK2 are pulled down by anti-ADAR1, but not by negative controls. Confocal immunofluorescence analysis confirms the interaction between ADAR1 and EIF2AK2 in the nucleus. Arrows indicate orthogonal views of colocalized ADAR1 and EIF2AK2. (B) ADAR1 and HuR interact in vivo in an RNA-dependent manner. RNase A and V1 treatment before IP abolishes the interaction between ADAR1 and HuR. (C) RNA pull-down experiments showed that HuR (top panel) and ADAR (bottom panel) bind to the same target transcripts. A (polyA)_25_ RNA was used as the negative-control transcript. Cell lysate incubated with mock solution before pull-down was included as the no-RNA control. (D) ADAR1 and HuR antibodies, but not control IgG, pulled down the same transcripts. Following anti-ADAR1 and anti-HuR RNA-IP, quantitative RT-PCR was carried out to measure the levels of various transcripts. RNA levels bound by negative-control IgG were normalized to one. (E) *ADAR1* knockdown leads to reduced binding of HuR to their target transcripts. HuR RNA-IP was carried out in cells treated with ADAR1-siRNA or NTC-siRNA, and the HuR-associated transcript level was measured by quantitative RT-PCR. (F) The gene expression of the target transcripts of HuR and ADAR1 was reduced following *ADAR1* knockdown. Gene expression levels from RNA-seq data (RPKM) were normalized to those obtained from NTC-siRNA samples. Error bar indicates SEM. See also Tables S2 and S4.

Our analysis of sequence motifs around editing sites identified an enrichment of HuR-binding motifs. This motivated us to study whether HuR and ADAR1 function cooperatively. HuR binds to single-stranded RNA (ssRNA) and regulates transcript stability and gene expression (Fan and Steitz, 1998). We carried out protein IP using anti-ADAR1 and negative-control IgG. We confirmed specific pull-down of ADAR1 by immunoblotting, and identification of transcripts and protein of EIF2AK2, a known editing target and interacting partner of ADAR, in the immunoprecipitates (Figure 6A; Clerzius et al., 2009). Using antibody against HuR, we found that HuR was pulled down with ADAR1, suggesting these two proteins interact in vivo (Figure 6B, lanes 4 and 5). As a control, ILF3, a protein that is known to interact with ADAR1 in a dsRNA-dependent manner, was also pulled down (Nie et al., 2005). Next, we asked whether the interaction between ADAR1 and HuR is dependent on scaffold RNAs. We carried out ADAR1-IP using RNase A- and RNase V1-treated whole-cell lysates. RNase A treatment, which digests ssRNA, abolished the interactions between HuR and ADAR1, but not the interactions between ILF3 and ADAR1. In contrast, the dsRNA-specific RNase V1 reduced the interactions between HuR and ADAR1, and between ILF3 and ADAR1 (Figure 6B, lanes 6–9). These results show that the interaction between HuR and ADAR1 is dependent on both ssRNA and dsRNA.

To examine how ADAR and HuR interact with their RNA targets, we carried out additional analyses. First, we studied the HuR-binding sites in ADAR-bound transcripts. As mentioned above, we found that sequences of transcripts bound by ADARs were enriched for AU-rich elements (AREs), which are HuR-binding sites. Among the 4,279 ADAR-bound transcripts, 4,198 (98%) had at least one and often many HuR-binding sites. There were 172,000 TA(T/A)TTTT sites in ADAR-bound transcripts, significantly more (χ^2^, p < 0.0001) than in control transcripts (68% of 4,279 random control transcripts contain 79,084 AREs). Similarly, other HuR-binding sequences (including (U/A) UUUA, (U/C)UUUA, and AUUU(U/C); Mukherjee et al., 2011) were also enriched in ADAR-bound transcripts. Second, since the presence of AREs does not mean that HuR binds to them, we confirmed the binding using PAR-CLIP data (Kishore et al., 2011; Lebedeva et al., 2011; Mukherjee et al., 2011). Among the 4,279 transcripts bound by ADAR1, 2,866 (67%) were also bound by HuR in PAR-CLIP, which is significantly more than observed in random transcripts (36%; χ^2^, p < 0.0001), showing that HuR binds to ADAR1 targets in vivo. These common binding targets of HuR and ADAR1 include *MCM4*, which plays a key role in DNA replication; *TMPO*, which encodes a nuclear membrane protein; and *GSR*, which encodes the enzyme glutathione reductase in the antioxidative stress pathway.

The enrichment of HuR-binding sites in ADAR targets and the identification of an RNA-dependent HuR-ADAR complex led us to reason that HuR and ADAR bind to common transcripts and regulate them cooperatively. We confirmed our hypothesis by employing two experimental approaches. First, we carried out RNA pull-down assays. We prepared in vitro synthesized and biotinylated RNA for three transcripts (*MCM4*, *CTH*, and *GSR*) that we previously identified as shared targets of ADAR and HuR. After incubating these transcripts with B cell lysates, we pulled down the transcripts using their biotin tags and immunoblotted for ADAR and HuR proteins. Our results showed that ADAR and HuR are specifically pulled down on *MCM4* and *GSR* transcripts, confirming concurrent ADAR and HuR binding (Figure 6C). Although it binds strongly to HuR, the *CTH* transcript pulled down less ADAR1, suggesting its weaker interaction with ADAR1 compared with *MCM4* and *GSR*. Second, we carried out HuR RNA-IP to confirm that HuR binds to the same transcripts that ADAR1 targets, and then examined the effects of such binding. Using HuR antibody, we pulled down HuR protein and tested whether ADAR1-targeted transcripts were pulled down with HuR in human B cells. The results showed that HuR antibody, but not negative-control IgG, pulled down the same transcripts that immunoprecipitated with ADAR1 antibody, including *MCM4*, *TMPO*, *GSR*, and *CTH* (Figure 6D).

We then asked whether HuR and ADAR1 depend on each other for binding to their common targets. Previous studies have found that HuR and other RNA-binding proteins cooperate by binding to the same RNA substrates (Chang et al., 2010; Lal et al., 2004). We carried out HuR RNA-IP following siRNA knockdown of *ADAR1* and found that following *ADAR1* knockdown, binding of HuR to its target transcripts is greatly reduced (Figure 6E). In cells transfected with *ADAR1* siRNAs, the protein level in HuR is the same as that in controls (Figure S2B), confirming that the decrease in HuR binding is not due to decreased HuR protein expression.

After identifying that ADAR is required for HuR binding to transcripts, we examined the effects of ADAR and HuR on transcript levels. Since HuR regulates gene expression by stabilizing mRNAs (Myer et al., 1997), we examined whether ADAR binding affects transcript stability through HuR. Among the 775 genes whose expression levels decreased following *ADAR1* knockdown, there were significantly more genes containing HuR-binding sites than genes whose expression levels increased following *ADAR1* knockdown (χ^2^, p < 0.01). For example, the expression levels of *MCM4*, *TMPO*, *GSR*, and *CTH* transcripts were reduced in both individuals following *ADAR1* knockdown, consistent with binding of their transcripts by both HuR and ADAR1 (Figure 6F). These results support the notion that in the absence of ADAR1, HuR binding decreased; thus, the target genes were not stabilized, resulting in lower gene expression. Lastly, these data suggest that *ADAR1* and *HuR* expression levels should correlate with the expression levels of their target genes. Using results from another study in our lab (Cheung et al., 2010), we compared the expression levels of the target genes with *ADAR1* and *HuR* in cultured B cells from 41 unrelated individuals and found that they were significantly correlated (p ≪ 0.01). Correlation plots for *MCM4* and *TMPO* with *ADAR1* and *HuR* are shown in Figure S4A.

Our findings suggest that ADAR1 and HuR proteins cooperate to regulate RNA processing through editing and mRNA turnover. These proteins coregulate transcripts by binding to specific sequences and secondary structures that mediate these processing steps.

## DISCUSSION

In this study, we uncovered ~60,000 A-to-G RNA editing sites mediated by ADAR1 and ADAR2 proteins in human B cells. We show that ADAR proteins are involved in gene regulation, particularly in regulating RNA stability and processing.

Prior to our study, many A-to-G editing sites had been identified. Here, we added to the list of such sites by using gene knockdown and RNA-IP, and we validated experimentally that our sites are direct targets of ADAR1 and ADAR2 proteins. Traditionally, editing sites are identified by comparing DNA and RNA sequences. Often the DNA sequences used for comparisons are those from the reference genome. We extracted the DNA and RNA from the same cells and subjected them to deep sequencing, which allowed a direct comparison of RNA sequences and their corresponding DNA. Although NGS provides sequence information with unprecedented coverage, there are hundreds of millions of sequence reads that have to be mapped correctly for proper interpretation. To have confidence in our sequence mapping, we set stringent analysis thresholds that required uniquely mapped reads from two different sequence alignment algorithms (GSNAP and blat) and at least ten sequence reads at each site. However, computational analysis alone may not be adequate. To determine a list of high-confidence ADAR targets, we coupled deep sequencing with *ADAR* gene knockdowns and ADAR RNA-IP. The same analysis method was used to analyze sequence reads from all samples, and thus the sites in which editing is responsive to gene knockdown, or that are bound specifically to ADAR proteins, cannot be artifacts of computational analyses. In a recent study on RNA editing in *Drosophila* (Rodriguez et al., 2012), RNA-seq of nascent RNA from an *ADAR* null strain was compared with that of a wild-type strain. The results were used to estimate an FDR of ~5%. In our study, we used a similar approach and estimated our FDR to be ~4%.

The large number of sites in which RNA sequences differed from the underlying DNA sequences is surprising and requires further attention in genetic studies. Results from this and other studies show that there are likely many thousands of A-to-G editing sites in each individual. Previously, we showed that there are individual differences in the number of RDDs (Li et al., 2011). Here, in our two subjects, we also observed differences in the number of editing sites and the level of editing. These results indicate that genetic variation can extend beyond DNA sequence variation. Even though two individuals may have the same DNA sequences at a site, their RNA sequences may differ. To date, most genetic studies have focused on DNA sequence variation in looking for disease-susceptibility alleles. As it becomes clear that RNA sequence variation extends beyond DNA sequence polymorphism, RNA editing and other types of RDDs will have to be considered in studies to identify the genetic basis of human diseases and traits. Comprehensive lists of editing and RDD sites, such as those presented in this study, are important for facilitating the inclusion of RNA variants in genetic studies.

RNA transcripts are tethered to regulatory factors, and the combinatorial binding of RBPs to transcripts coordinates different steps of RNA processing (Hogan et al., 2008; Licatalosi and Darnell, 2010; Maniatis and Reed, 2002). We found enrichment of binding sequences for HuR in transcripts edited by ADAR. Computational and experimental evidence from HuR RNA-IP in human B cells and cells transfected with ADAR siRNAs showed that HuR binding is facilitated by ADAR binding to RNAs. Our results are consistent with a model in which binding of ADAR to RNA forms secondary structures that are then recognized by HuR proteins. Thus, RNA sequences and structures allow gene regulation by a combination of different RNA processing proteins. Transcription factors cooperate to mediate gene regulation; similarly, RNA processing proteins coordinate to affect gene expression. The complex regulatory codes involve RNA sequences and structures that are facilitated by different combinations of RNA-binding proteins. Therefore, to understand co- and posttranscriptional regulation of gene expression, we need to go beyond studying single proteins. Experimental methods that examine protein complexes and their target RNAs are needed to enhance our understanding of gene regulation.

In summary, in this work we studied ADAR-mediated RNA editing and gene-expression regulation. Our findings uncover editing targets, reveal ADARs’ role in mediating RNA editing and regulation of gene expression, and show that the ADAR protein complex coordinates multiple steps in RNA processing. However, they also raise new questions. Our findings suggest that other mechanisms, such as those that mediate non-A-to-G type RDDs, remain to be identified. In addition, the RNA sequence and structural signatures of the regulatory codes for co- and posttranscriptional processing are largely unknown. Elucidating ADAR’s functions will further our understanding of RNA processing and provide insights into human diseases.

## EXPERIMENTAL PROCEDURES

### Identification of Editing and RDDs

B cell lines from two individuals in the Centre d’Étude du Polymorphisme Humain database were cultured and genomic DNA and RNA were extracted. DNA-seq and RNA-seq libraries were prepared and sequenced on a HiSeq 2000 instrument (Illumina). DNA-seq and RNA-seq data were aligned to the reference genome (HG18) using CASAVA and GSNAP, respectively. To identify RDDs, we compared each RNA sequence with its corresponding DNA sequence. We required an editing site or RDD site to be covered by a minimum of 10 total DNA-seq and RNA-seq reads, 100% concordance in the DNA sequence, an RDD level ≥ 10%, and an RDD event to be found in both individuals. Potential sites were then filtered using stringent thresholds.

### Validation of RDDs using Sanger Sequencing and Droplet Digital PCR

Cultured B cells were transfected with Accell siRNAs (Thermo Scientific) against *ADAR1* and *ADAR2*. Sequences surrounding RDD sites were PCR amplified using genomic DNA or cDNA as the template, and PCR products were sequenced. The 3′ UTR of ATM was amplified from cDNA of B cells and cloned into TOPO vector (Invitrogen).

For droplet digital PCR, DNA probes specific to the DNA and RNA variants at RDD sites were synthesized and labeled by VIC and FAM, respectively (ABI Biosystems). Emulsion PCR was carried out and quantified on a QuantaLIfe Droplet Reader (Bio-Rad Laboratories).

### RNA-IP

Anti-ADAR1 and anti-HuR RNA-IP was carried out with a Magna RNA-Binding Protein Immunoprecipitation Kit (Millipore). Quantitative PCR and RNA-seq of immunoprecipitated transcripts were carried out. RNA-editing sites that were detected in transcripts pulled down by ADAR1 antibody, but not by negative-control IgG, were identified as ADAR1-specific targets.

### RNA-Protein Pull-Down Assays

Transcripts of HuR and ADAR1 targets were synthesized and biotin labeled in vitro, and incubated with whole-cell lysates. RNA-protein complexes were pulled down and analyzed by western blot (Pierce).

### Protein IP of the ADAR-HuR Complex

B cell lysates were incubated with anti-ADAR1 or negative-control rabbit IgG at 4°C overnight. The immunocomplex was pulled down using Protein A agarose (Roche), washed, and finally eluted in 20 mM Tris/7.5, 150 mM NaCl, 2.5 mM MgCl_2_, 0.2% SDS. To examine RNA-dependent interactions, whole-cell lysates were diluted to 1 μg/μl, RNase A or RNase V1 was added, and lysates were incubated at room temperature for 15 min. Protein samples were analyzed by western blot.

A detailed description of the materials and methods used in this work is provided in the Supplemental Experimental Procedures.

## Supplementary Material

1

2

3

4

5

6

7

8

## Figures and Tables

**Table 1:** Hyperedited Transcripts

Hyperedited Region	Gene Symbol	Number of Edited Sites
chr9:131701274-131841654	*FNBP1*	291
chr3:47608175-47795690	*SMARCC1*	218
chr1:1713762-1810015	*GNB1*	214
chr4:39379937-39452078	*UBE2K*	167
chr5:138923557-138985907	*UBE2D2*	162
chr8:98728784-98810463	*MTDH*	154
chr15:42553696-42603223	*CTDSPL2*	148
chr1:149438531-149485085	*PIP5K1A*	141
chr10:70152450-70219274	*CCAR1*	138
chr17:24746313-24892874	*TAOK1*	136
chr16:68968176-69027669	*ST3GAL2*	134
chr5:176497466-176651020	*NSD1*	134
chr2:61559886-61613430	*XPO1*	133
chr3:49046623-49101020	*QRICH1*	131
chr12:49088991-49144511	*LARP4*	128
chr16:15655703-15700735	*NDE1*	128
chr1:149652924-149695116	*POGZ*	127
chr19:17076438-17180447	*MYO9B*	127
chr19:16604467-16625697	*C19orf42*	125
chr16:88337701-88409128	*FANCA*	123
