# Supplementary material for: ADAR Regulates RNA Editing, Transcript Stability, and Gene Expression
Source: Cell Rep. Author manuscript; Available in PMC 2014 Nov 14. (PMC3935819; doi:10.1016/j.celrep.2013.10.002)
Supplement: 1 [file NIHMS543889-supplement-1.pdf]

ADAR Regulates RNA Editing, Transcript Stability and Gene Expression

Isabel X. Wang, Elizabeth So, James L. Devlin, Yue Zhao, Ming Wu, Vivian G. Cheung

Supplemental Figure Legends

Figure S1. Validation of A-to-G editing, related to Figure 2. (A) An example of validation of A-to-G editing by droplet digital PCR. Shown here is the editing site at chr5:49728037 in *EMB*. Genomic DNA or cDNA from GM12750 was used as input for PCR amplified by gene-specific primers and fluorescence-labeled allele-specific probes. (B) Summary of sites that are validated experimentally.

Figure S2. Examined off-target effects of siRNA by testing 4 independent specific siRNAs and pooled siRNAs, related to Figure 3, 4 and 6. (A) mRNA expression levels of *ADAR1* and *ADAR2* are reduced by 4 individual siRNAs and pooled siRNA, shown by real-time RT-PCR. (B) Western blot confirms reduced ADAR1 at protein level. In contrast, HuR expression is not affect by *ADAR1* knockdown. (C) Sanger sequencing confirms that knockdown of *ADAR1* by each siRNA led to reduced editing levels at chr2:37184007. Sequence traces represent the reverse strand of cDNA. Error bar: SEM of triplicate experiments.

Figure S3. Dynamic changes in editing levels and gene expression following *ADAR1* knockdown, related to Figure 3. (A) A 96-hour time course of ADAR1-knockdown combined with RNAseq uncovered dynamic changes in editing levels. As a result of ADAR1-siRNA treatment, mRNA levels of ADAR1 remained low (<50% of control sample) for up to 96 hours post-transfection. (B) Examples of A-to-G sites where the editing levels remained low throughout the time-course. (C) Example of genes that did not change significantly at total gene level but showed “isoform switching” following *ADAR1* knockdown.

Figure S4. Correlation of gene expression between ADAR1 and HuR, and overlap between our study and previous published studies, related to Figure 3 and 6. (A) Gene expression level of HuR and ADAR1 among 41 unrelated individuals are significantly correlated with that of their target genes (P<10-7). Gene expression levels (RPKM) of HuR, ADAR1, MCM4 and TMPO were calculated from RNA-seq data of 41 unrelated individuals. (B)&(C) Editing sites identified in current study (GM12004 and GM12750) are compared to those from (B) all previously published data or (C) Peng et al, 2012. Comparison is based on overlapping (by site) or overlapping genes (by gene).

A

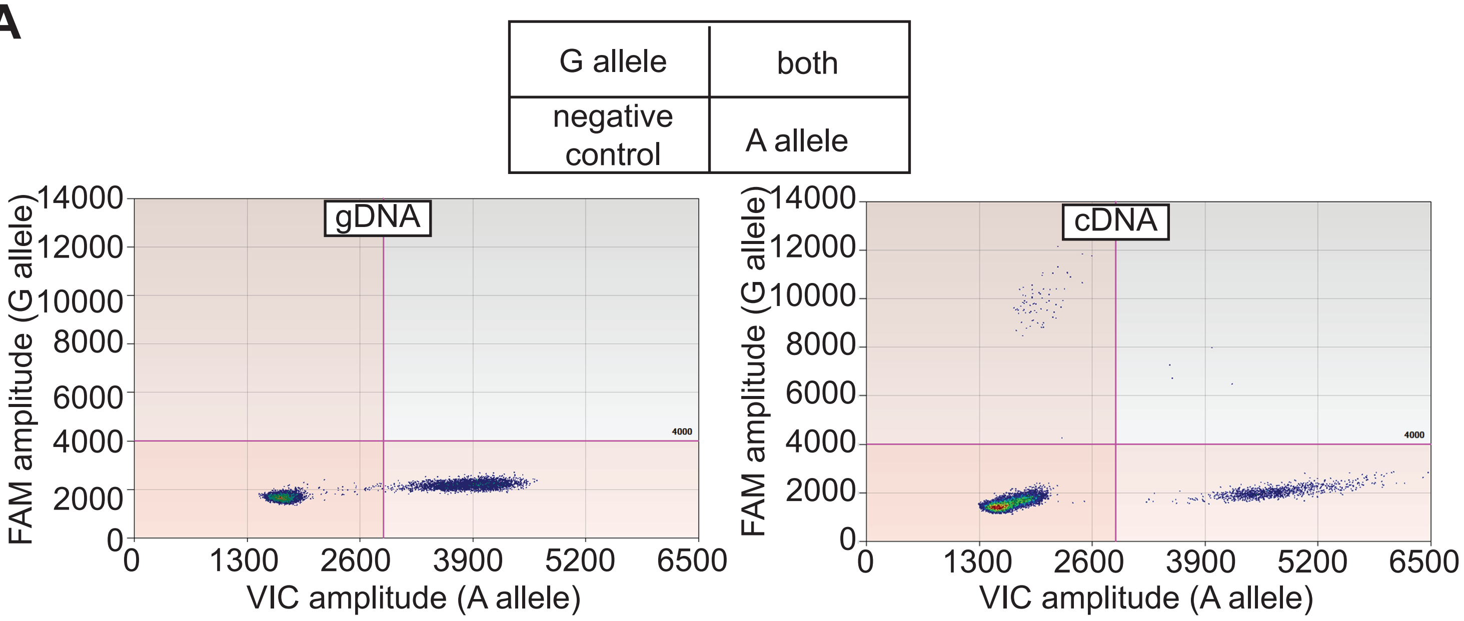

B

Chr	Position	Gene Symbol	Type	Location	Strand	In Alu	Average Level	Validation method	Validated?
11	107741679	*ATM*	A>G	3UTR	+	Yes	0.62	Cloning/Sanger	Yes
11	107741687	*ATM*	A>G	3UTR	+	Yes	0.49	Cloning/Sanger	Yes
11	107741695	*ATM*	A>G	3UTR	+	Yes	0.46	Cloning/Sanger	Yes
11	107741696	*ATM*	A>G	3UTR	+	Yes	0.44	Cloning/Sanger	Yes
11	107741723	*ATM*	A>G	3UTR	+	Yes	0.07	Cloning/Sanger	Yes
11	107741733	*ATM*	A>G	3UTR	+	Yes	0.38	Cloning/Sanger	Yes
11	107741734	*ATM*	A>G	3UTR	+	Yes	0.12	Cloning/Sanger	Yes
11	107741810	*ATM*	A>G	3UTR	+	Yes	0.08	Cloning/Sanger	Yes
11	107741819	*ATM*	A>G	3UTR	+	Yes	0.21	Cloning/Sanger	Yes
11	107741827	*ATM*	A>G	3UTR	+	Yes	0.24	Cloning/Sanger	Yes
11	107741845	*ATM*	A>G	3UTR	+	Yes	0.18	Cloning/Sanger	Yes
11	107741851	*ATM*	A>G	3UTR	+	Yes	0.24	Cloning/Sanger	Yes
6	90487981	*MDN1*	C>A	intron	+	No	1	PCR/Sanger	Yes
7	89857293	*GTPBP10*	A>G	3UTR	+	Yes	0.63	PCR/Sanger	Yes
7	89857303	*GTPBP10*	A>G	3UTR	+	Yes	0.09	PCR/Sanger	Yes
9	32446365	*DDX58*	A>G	3UTR	-	No	0.07	PCR/Sanger	Yes
9	32446368	*DDX58*	A>G	3UTR	-	No	0.12	PCR/Sanger	Yes
9	32446371	*DDX58*	A>G	3UTR	-	No	0.1	PCR/Sanger	No
10	74678737	*MRPS16*	A>G	3UTR,stop_codon	-	Yes	0.35	PCR/Sanger	Yes
10	114199337	*VTI1A*	A>G	mixed, exon	+	Yes	0.38	PCR/Sanger	Yes
10	114199343	*VTI1A*	A>G	mixed, exon	+	Yes	0.27	PCR/Sanger	Yes
14	19905006	*TEP1*	A>G	3UTR	-	No	0.11	PCR/Sanger	Yes
14	52179482	*ERO1L*	A>G	3UTR	-	No	0.05	PCR/Sanger	Yes
14	52179560	*ERO1L*	A>G	3UTR	-	No	0.15	PCR/Sanger	Yes
1	159227224	*F11R*	A>G	intergenic	-	No	0.2	droplet digital PCR	Yes
2	20314300	*PUM2*	A>G	3UTR	-	No	0.21	droplet digital PCR	Yes
3	151819253	*SELT*	A>G	mixed, exon	+	Yes	0.22	droplet digital PCR	No
5	49728037	*EMB*	A>G	3UTR	-	No	0.08	droplet digital PCR	Yes
5	67632909	*PIK3R1*	A>G	3UTR	+	No	0.1	droplet digital PCR	Yes
21	33845189	*SON*	A>G	coding exon	+	No	0.15	droplet digital PCR	Yes

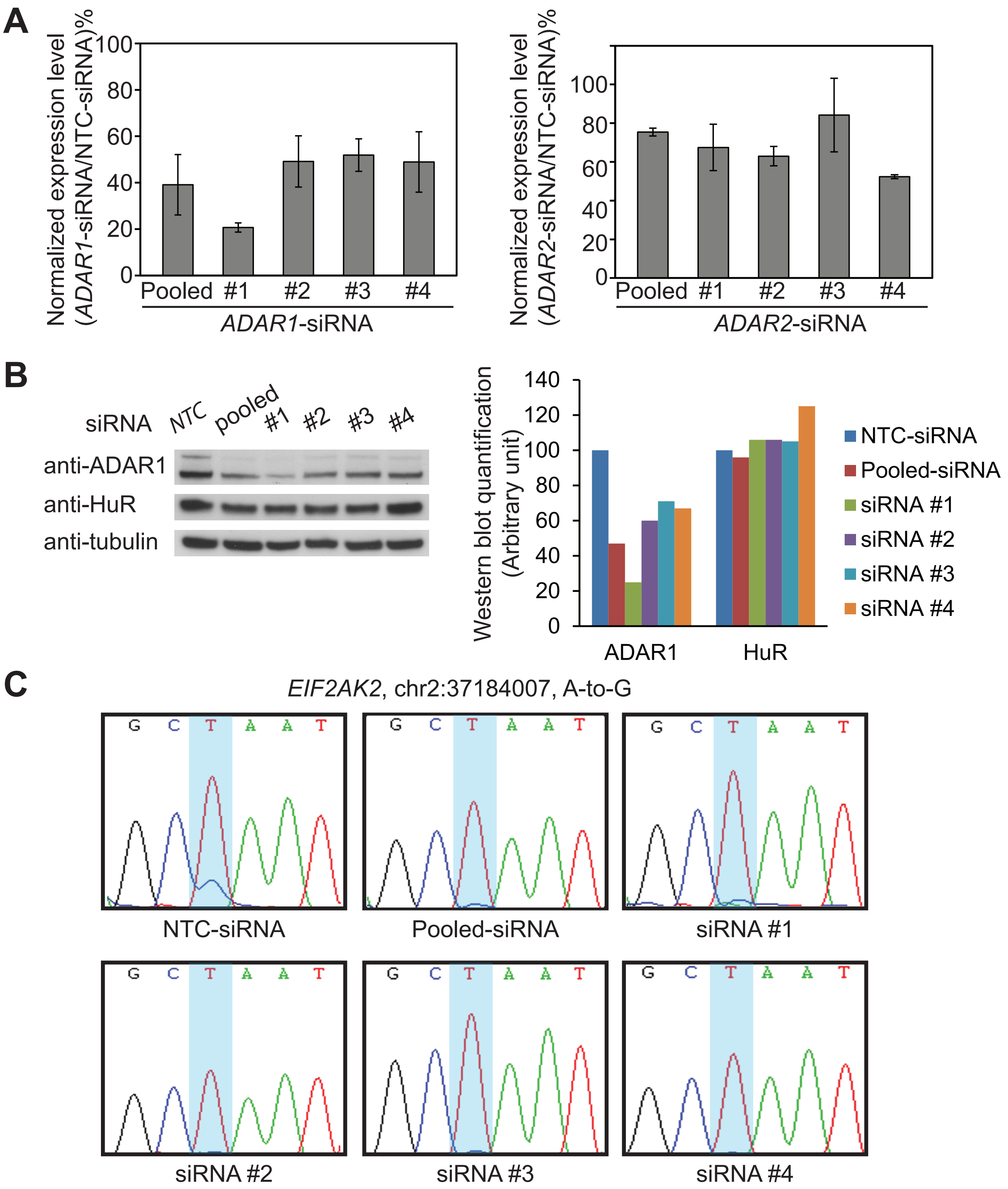
A
Normalized expression level (ADAR1-siRNA/NTC-siRNA)%
0
20
40
60
80
100
Pooled
#1
#2
#3
#4
ADAR1-siRNA
Normalized expression level (ADAR2-siRNA/NTC-siRNA)%
0
20
40
60
80
100
Pooled
#1
#2
#3
#4
ADAR2-siRNA
B
siRNA
NTC
pooled
#1
#2
#3
#4
anti-ADAR1
anti-HuR
anti-tubulin
Western blot quantification (Arbitrary unit)
0
20
40
60
80
100
120
140
ADAR1
HuR
NTC-siRNA
Pooled-siRNA
siRNA #1
siRNA #2
siRNA #3
siRNA #4
C
EIF2AK2, chr2:37184007, A-to-G
G C T A A T
NTC-siRNA
Pooled-siRNA
siRNA #1
siRNA #2
siRNA #3
siRNA #4

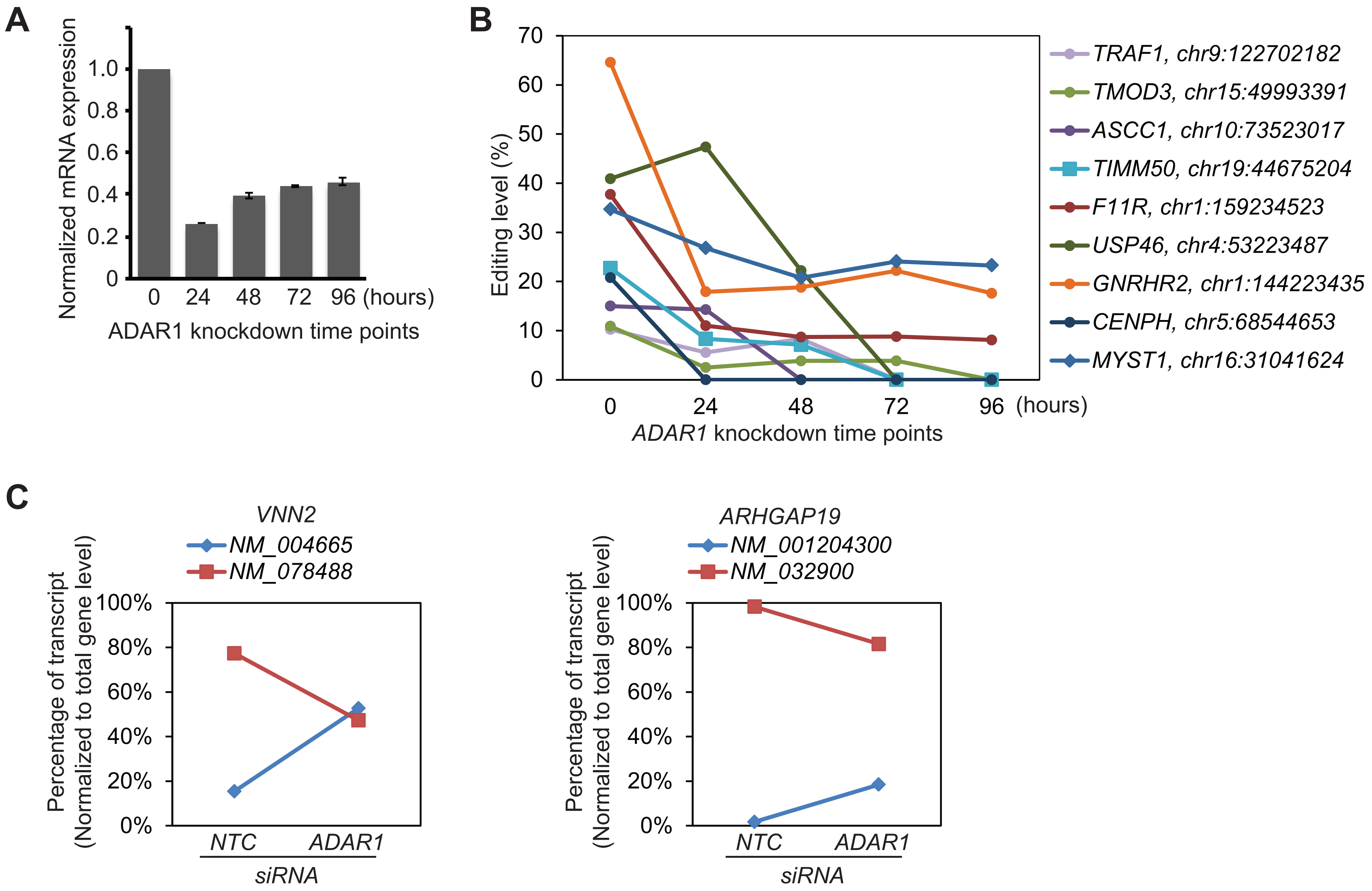
A
Normalized mRNA expression
1.0
0.8
0.6
0.4
0.2
0
0 24 48 72 96 (hours)
ADAR1 knockdown time points
B
Editing level (%)
70
60
50
40
30
20
10
0
0 24 48 72 96 (hours)
ADAR1 knockdown time points
TRAF1, chr9:122702182
TMOD3, chr15:49993391
ASCC1, chr10:73523017
TIMM50, chr19:44675204
F11R, chr1:159234523
USP46, chr4:53223487
GNRHR2, chr1:144223435
CENPH, chr5:68544653
MYST1, chr16:31041624
C
VNN2
NM_004665
NM_078488
Percentage of transcript (Normalized to total gene level)
100%
80%
60%
40%
20%
0%
NTC ADAR1
siRNA
ARHGAP19
NM_001204300
NM_032900
Percentage of transcript (Normalized to total gene level)
100%
80%
60%
40%
20%
0%
NTC ADAR1
siRNA

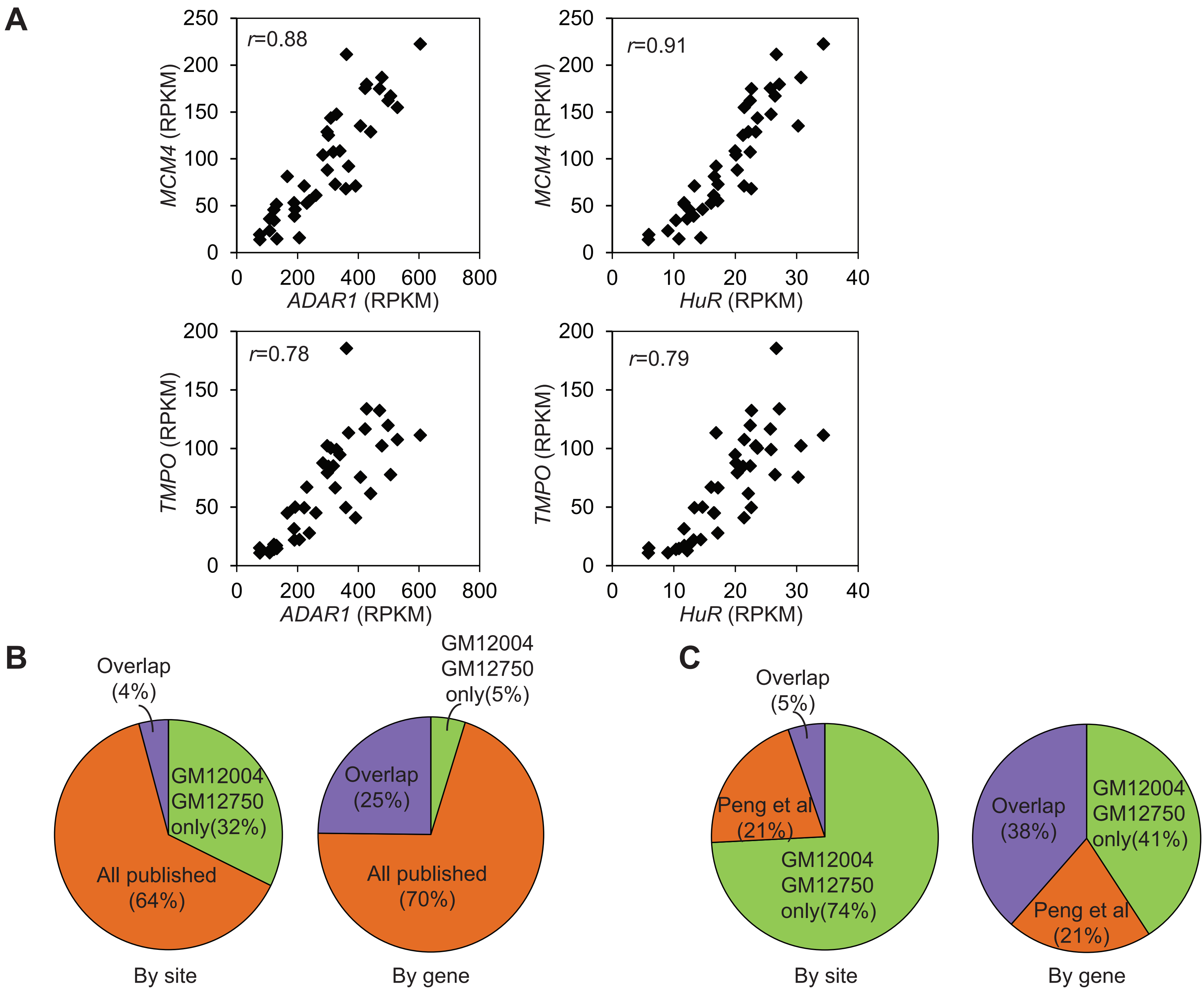
A
r=0.88
MCM4 (RPKM)
ADAR1 (RPKM)
r=0.91
MCM4 (RPKM)
HuR (RPKM)
r=0.78
TMPO (RPKM)
ADAR1 (RPKM)
r=0.79
TMPO (RPKM)
HuR (RPKM)
B
Overlap (4%)
GM12004 GM12750 only(32%)
All published (64%)
By site
GM12004 GM12750 only(5%)
Overlap (25%)
All published (70%)
By gene
C
Overlap (5%)
Peng et al (21%)
GM12004 GM12750 only(74%)
By site
Overlap (38%)
GM12004 GM12750 only(41%)
Peng et al (21%)
By gene

Supplemental Table Titles

Table S1 Summary of RNA-seq data, related to Figure 1.

Table S2 RNA-DNA differences identified from analysis of RNA-seq and DNA-seq of B-cells from two individuals, related to Figure 1.

Table S3 ADAR1 knockdown leads to changes in editing levels of target genes, related to Figure 3.

Table S4 ADAR2 knockdown leads to changes in editing levels of target genes, related to Figure 4.

Table S5 Editing sites identified from anti-ADAR1 RNA-immunoprecipitation experiment, related to Figure 4.

Table S6 Genes whose expression levels changed significantly following *ADAR1* knockdown in both individuals, related to Figure 3.

Table S7 Gene ontology analysis of genes whose expression levels changed following ADAR1 knockdown, related to Figure 3.

Table S8 Genes whose expression levels and editing levels both changed at least 20% following ADAR1 knockdown, related to Figure 5.

Table S9 Genes whose expression levels changed at least 2 fold following ADAR2 knockdown, related to Figure 4.

Table S10 Primers used in this study, related to experimental procedures.

Table S1. Summary of RNA-seq data, related to Figure 1.

**Sample ID in GEO(GSE38233)**	**Sample name in GEO**	**Individual**	**Experiment**	**Library insert size**	**Sequencing**	**Average Read length**	**Total # of reads**	**#aligned reads**	**# uniquely aligned reads**	**% of aligned reads**	**% of unquely aligned reads**
Sample 1	Sample_GM12750_baseline	GM12750	baseline	200-350 bp	Single-end	97 nt	142,255,567	135,660,583	112,735,386	95.4%	79.2%
Sample 2	Sample_GM12004_baseline	GM12004	baseline	200-350 bp	Single-end	98 nt	175,654,256	171,103,388	139,411,120	97.4%	79.4%
Sample 3	Sample_GM12750_Directional	GM12750	baseline, directional	180-200 bp	Single-end	97 nt	206,154,747	172,813,413	139,340,950	83.8%	67.6%
Sample 4	Sample_GM12004_Directional	GM12004	baseline, directional	180-200 bp	Single-end	97 nt	201,366,748	170,030,478	135,316,699	84.4%	67.2%
Sample 5	Sample_GM12750_Directional_ADAR_KD	GM12750	ADAR1-siRNA, directional	180-200 bp	Single-end	96 nt	186,855,107	136,709,952	111,042,298	73.2%	59.4%
Sample 6	Sample_GM12750_Directional_NT	GM12750	NTC siRNA, directional	180-200 bp	Single-end	95 nt	149,184,629	80,370,194	64,979,507	53.9%	43.6%
Sample 7	Sample_GM12750_NT	GM12750	NTC siRNA	200-350 bp	Single-end	97 nt	82,666,415	80,465,767	67,257,829	97.3%	81.4%
Sample 8	Sample_GM12750_ADAR_KD	GM12750	ADAR1 siRNA	200-350 bp	Single-end	96 nt	114,729,241	111,689,921	93,568,018	97.4%	81.6%
Sample 9	Sample_GM12004_NT	GM12004	NTC siRNA	200-350 bp	Single-end	98 nt	188,841,363	183,119,621	150,857,634	97.0%	79.9%
Sample 10	Sample_GM12004_ADAR_KD	GM12004	ADAR1 siRNA	200-350 bp	Single-end	98 nt	196,982,179	191,523,523	156,668,557	97.2%	79.5%
Sample 11	Sample_GM12004_ADAR2_kd	GM12004	ADAR2 siRNA	200-350 bp	Single-end	98 nt	188,282,790	183,311,559	152,896,261	97.4%	81.2%
Sample 12	Sample_GM12750_ADARkd_ATime	GM12750	ADAR1-siRNA, time point 24 hour	200-350 bp	Single-end	98 nt	171,412,869	166,839,294	141,015,077	97.3%	82.3%
Sample 13	Sample_GM12750_ADARkd_BTime	GM12750	ADAR1-siRNA, time point 48 hour	200-350 bp	Single-end	98 nt	192,108,845	186,522,411	156,761,432	97.1%	81.6%
Sample 14	Sample_GM12750_ADARkd_CTime	GM12750	ADAR1-siRNA, time point 72 hour	200-350 bp	Single-end	98 nt	192,043,296	186,020,035	152,778,114	96.9%	79.6%
Sample 15	Sample_GM12750_ADARkd_DTime	GM12750	ADAR1-siRNA, time point 96 hour	200-350 bp	Single-end	98 nt	188,777,483	182,741,246	149,791,979	96.8%	79.3%
Sample 16	Sample_GM12750_nt_Dtime	GM12750	NTC siRNA, time point 96 hour	200-350 bp	Single-end	98 nt	185,192,178	177,125,378	146,721,291	95.6%	79.2%
Sample 17	Sample_RNA-IP_ADAR_GM12004	GM12004	ADAR1 RNA-IP	200-350 bp	Single-end	98 nt	186,593,821	119,606,085	91,717,555	64.1%	49.2%
Sample 18	Sample_RNA-IP_IgG_GM12004	GM12004	control IgG RNA-IP	200-350 bp	Single-end	98 nt	204,394,300	130,890,544	98,581,153	64.0%	48.2%
Sample 19	Sample_RNA-IP_ADAR_KD_GM12750	GM12750	ADAR1 RNA-IP	200-350 bp	Single-end	98 nt	213,942,446	145,198,134	110,025,519	67.9%	51.4%
Sample 20	Sample_RNA-IP_IgG_GM12750	GM12750	control IgG RNA-IP	200-350 bp	Single-end	98 nt	215,323,408	146,778,885	111,075,019	68.2%	51.6%

Table S2 RNA-DNA differences identified from analysis of RNA-seq and DNA-seq of B-cells from two individuals, related to Figure 1.

Table S3 ADAR1 knockdown leads to changes in editing levels of target genes, related to Figure 3.

Table S4 ADAR2 knockdown leads to changes in editing levels of target genes, related to Figure 4.

Table S5 Editing sites identified from anti-ADAR1 RNA-immunoprecipitation experiment, related to Figure 4.

Table S6 Genes whose expression levels changed significantly following *ADAR1* knockdown in both individuals, related to Figure 3.

Table S7. Gene ontology analysis of genes whose expression levels changed following *ADAR1* knockdown, related to Figure 3.

Gene Ontology Term	# Gene	P-Value	Examples
Cell cycle	111	$8.5X10^{-18}$	*JUB, AURKA, SEPT8, CENPE, CDC25C, CDKN3*
ATP binding	133	$6.6X10^{-11}$	*MCM4, ATP8A1, KIFC1, ZAK, DTYMK, DSTYK*
Kinase	81	$5.4X10^{-10}$	*ZAK, PHKB, DTYMK, DSTYK, TTK, AURKA*
DNA damage response	57	$2.2X10^{-10}$	*MLH1, NEIL1, RAD52, RAD51, ATRIP, EXO1*
Chromosome segregation	22	$8.8X10^{-9}$	*NEK2, CENPE, BIRC5, SMC2, SMC4, CENPH*
Ubiquitin-like protein conjugation	64	$7.3X10^{-7}$	*UBE2C, UBE2S, USP1, ATG10, RNF14, USP1*
Zinc-finger	143	$1.8X10^{-6}$	*THRA, PLEKHM1, ZNF580, ZNF451, MCM10, ZNF251*
Chromatin	24	0.0004	*TMPO, HMGN1, HIST1H2AC, HMGN2, ARID4B, HDAC2*

Table S8. Genes whose expression levels and editing levels both changed at least 20% following *ADAR1* knockdown, related to Figure 5.

Gene Symbol	# Edited sites	Type	Chromosome	Average change in editing level (%)	Average change in FPKM (%)
AMD1	4	A>G	6	-99	-60
ANKDD1A	1	A>G	15	-100	30
AP3S2	3	A>G	15	-86	60
APC2	4	A>G	19	-47	60
APOL1	13	A>G	22	-76	-30
ARRDC2	1	A>G	19	-12	90
ATG12	3	A>G	5	-86	50
ATP5S	30	A>G	14	-86	40
AURKA	1	A>G	20	-93	-40
B4GALT2	1	A>G	1	-16	-40
BLCAP	1	A>G	20	-52	-70
C14orf118	5	A>G	14	-76	40
C15orf23	1	A>G	15	-66	60
C15orf40	3	A>G	15	-45	70
C16orf5	1	A>G	16	-100	70
C17orf85	1	A>G	17	-65	90
C19orf12	1	A>G	19	-100	30
CALR3	1	A>G	19	-13	-40
CARD8	7	A>G	19	-88	270
CBFA2T2	3	A>G	20	-100	-60
CBFB	1	A>G	16	-100	-20
CBX5	2	A>G	12	-86	50
CCDC84	6	A>G	11	-96	30
CD302	1	A>G	2	-100	50
CENPN	35	A>G	16	-78	-50
CFLAR	9	A>G	2	-86	-30
CHST12	1	A>G	7	-84	-50
CLTA	4	A>G	9	-42	-40
CPNE1	4	A>G	20	-100	40
CRCP	32	A>G	7	-71	70
CTH	4	A>G	1	-97	-30
DAP3	1	A>G	1	-100	90
DCLRE1C	3	A>G	10	-73	40
DCTN5	5	A>G	16	-76	-30
DHFRL1	2	A>G	3	-96	-50
DMTF1	1	A>G	7	-82	-30
ECHDC2	1	A>G	1	-100	30
EEF1D	1	A>G	8	9	120
EGR1	1	A>G	5	100	30
EIF2AK2	54	A>G	2	-87	120
EIF3L	2	A>G	22	-100	180
EPB41	1	A>G	1	-1	-70
FAM184B	1	A>G	4	-100	20
FAM55C	5	A>G	3	-78	50
FCER2	24	A>G	19	-86	30
FDPS	2	A>G	1	-70	40
FLNB	1	A>G	3	-88	-50
GSDMB	2	A>G	17	-93	-40
GSR	13	A>G	8	-91	-60
HAUS2	24	A>G	15	-89	-50
HM13	2	A>G	20	-100	60
IFNAR2	15	A>G	21	-84	-50
IKZF3	62	A>G	17	-83	40
IL15	1	A>G	4	-100	-30
ILDR1	1	A>G	3	-100	40
IQCB1	2	A>G	3	-72	-30

Gene Symbol	# Edited sites	Type	Chromosome	Average change in editing level (%)	Average change in FPKM (%)
LYRM2	3	A>G	6	-97	-30
MAVS	69	A>G	20	-70	330
MCART1	3	A>G	9	-90	-30
MCM4	5	A>G	8	-94	-40
MDM4	9	A>G	1	-50	30
NARF	3	A>G	17	-94	190
NASP	1	A>G	1	-45	-40
NDUFS1	31	A>G	2	-86	230
NIF3L1	1	A>G	2	-25	250
PAIP1	3	A>G	5	-47	60
PARVB	2	A>G	22	-73	130
PARVG	5	A>G	22	-86	-60
PEX26	17	A>G	22	-86	-30
PHF7	2	A>G	3	-91	-60
PILRA	1	A>G	7	-85	40
PML	2	A>G	15	-18	259770
PPHLN1	4	A>G	12	-66	340
PPP2R1B	4	A>G	11	-90	-60
PRKRA	27	A>G	2	-83	100
PUS1	2	A>G	12	-100	-60
RASSF1	1	A>G	3	-100	-40
RBBP4	2	A>G	1	-66	14510
RCC1	6	A>G	1	-85	30
RFFL	1	A>G	17	-94	30
RPL28	3	A>G	19	-67	50
RPL36A	2	A>G	X	-50	30
RSRC2	1	A>G	12	-100	70
SERBP1	5	A>G	1	-96	90
SH3BP2	6	A>G	4	-43	-30
SLC3A2	1	A>G	11	-34	70
SNX1	1	A>G	15	-69	60
TAPBP	1	A>G	6	-73	30
TCP11L1	7	A>G	11	-84	-40
TFDP2	12	A>G	3	-74	30
TGOLN2	2	A>G	2	-63	9490
TMEM116	2	A>G	12	0	-40
TMPO	16	A>G	12	-85	-30
TRIM5	3	A>G	11	-100	120
TRIM66	1	A>G	11	-38	30
UBE2H	1	A>G	7	-66	70
VNN2	1	A>G	6	-100	170
XIAP	42	A>G	X	-87	180
ZNF276	2	A>G	16	11	60
ZNF397	5	A>G	18	-97	30
ZNF573	1	A>G	19	-100	110
ZNF611	5	A>G	19	-86	60
ZNF615	2	A>G	19	-61	20
ZNF621	7	A>G	3	-52	-40
ZNF841	4	A>G	19	-86	30
ZNF844	3	A>G	19	-100	30

Table S9 Genes whose expression levels changed at least 2 fold following ADAR2 knockdown, related to Figure 4.

Table 10. Primers used in this study, related to experimental procedures.

**Primers using in Sanger Sequencing**						
**Chr**	**Position**	**Gene Symbol**	**Forward primer (external)**	**Reverse primer (external)**	**Forward primer (internal)**	**Reverse primer (internal)**
11	107741679 - 107741851	ATM	N/A	N/A	5'-CATACAGCAGGCCATAGACC-3'	5'-GGCCCACAGCAACCTTCACCTCCCAG-3'
6	90487981	MDN1	N/A	N/A	5'-TGGCTTGACTACCCTGTGTG-3'	5'-CATGCTTGCTTGTTGTGGTC-3'
7	89857293, 89857303	GTPBP10	5'-ACAGTGGACAAAAAGTGTTGGCAGA-3'	5'-AGCTGGCTACTGACACCCACTCA-3'	5'-CAGGGTCTCT CTGTGGAGTG-3'	5'-CTAATACATTTCCCTCTGCT-3'
9	32446365, 32446368,32446371	DDX58	N/A	N/A	5'-TGGCTACACAGAGAACATGAGAA-3'	5'-GAGAACATATTAATAGGGCAAGATG-3'
10	74678737	MRPS16	5'-GGGAGCTTTGACAACCACAG-3'	5'-AAGATACCATTGGGGCAGTG-3'	5'-CTAGCCAAATTATGTAATGT-3'	5'-ACAGCAAGACCCCGTCTCTA-3'
10	114199337, 114199343	VTI1A	5'-TCCACTGCCGGAGATCGTCTTGA-3'	5'-TGAAGCTCCTGTGTTTGGGCCT-3'	5'-TGAGGTCGGGAGTTTGAGAC-3'	5'-AATCTCAGCTCATCGCAACC-3'
14	19905006	TEP1	N/A	N/A	5'-CCCGGCCCATAAAAGAACT-3'	5'-AAAGAAAGGGCCTGAGGAAA-3'
14	52179482, 52179560	ERO1L	5'-GGAAAAATTTGTGTGTGTGTGTG-3'	5'-CTTTAATAATGTGGTACAAAT-3'	5'-TAGAAATCTCCTGTGCCTTT-3'	5'-AAAATGCTAAGGAGGCCTCA-3'
**Primers and oligos used for droplet digital PCR**						
**Chr**	**Position**	**Gene Symbol**	**Forward primer (external)**	**Reverse primer (external)**	**VIC probe**	**FAM probe**
1	159227224	F11R	5'-TATTTGGAAATCCCTAACAGAATTGAGTTT-3'	5'-CCTTGACTGATGGCTTCATTAGCAT-3'	5'-TTCTTTTTGGATCCTTAATAGA-3'	5'-CTTTTTGGATCCCTAATAGA-3'
2	20314300	PUM2	5'-AACAGATTAACAATCAACTGCATAAATATT-3'	5'-CTCGTTATTTGCATGATAGTTTGTGAATT-3'	5'-ATCAAAATACAACTTAACTCTT-3'	5'-AAAATACAACTCAACTCTT-3'
3	151819253	SELT	5'-ACTCCTGGGATCAAGTGAACCT-3'	5'-TCACATCTGTAATCCCAGCACTTTG-3'	5'-CTGCCTCAGCCTCC-3'	5'-TGCCTCGGCCTCC-3'
5	49728037	EMB	5'-GAGAGACCAACAAATGTATATTTATAACACAGAGT-3'	5'-CCCATACCTGGTAGAGCATGTAC-3'	5'-AACTCCACATTTATTTGTG-3'	5'-CTCCACATTTGTTTGTG-3'
5	67632909	PIK3R1	5'-TCCAACTTAACATGAAACTTGTCACCAT-3'	5'-ACACACACACACACACACACATATA-3'	5'-AGATAGCATTAGCTGCCC-3'	5'-ATAGCATTGGCTGCCC-3'
21	33845189	SON	5'-GCAGCCTGTGGCAACTG-3'	5'-GCCCCAGCTGCCATGA-3'	5'-CAGGCAACTCTAGTGCC-3'	5'-AGGCAACTCCAGTGCC-3'
**Primers used for RNA pull-down experiments**						
**Gene Symbol**	**Forward primer (with T7 promoter)**			**Reverse primer**		
MCM4	5'-TAATACGACTCACTATAGGAGCCTTGTGAGCAAGGAAGGCTCCC-3'			5'-TTAAAGGTTTCAGAAATTATTTAT-3'		
GSR	5'-TAATACGACTCACTATAGGCTTGAGCCCAGGAGTTCAAGAC-3'			5'-CTTTTCCCAGGTGGTTGGGAT-3'		
CTH	5'-TAATACGACTCACTATAGGTATTCCAGAGCTGCTATTAGAAGCT-3'			5'-CCTCAGCCTCCCAAGTAGCTGGGAC-3'		

Extended Experimental Procedures

Cell culture. Cultured B-cell lines from two CEPH individuals (Centre d'Etude du Polymorphisme Humain), GM12004 and GM12750, were obtained from Coriell Cell Repositories (Camden, NJ, USA). The B-cells were grown to a density of $5x10^5$ cells/mL in RPMI 1640 supplemented with 15% fetal bovine serum, 100 units/mL penicillin and 100 μg/mL streptomycin, and 2 mmol/L L-glutamine. For downstream experiments, cells were harvested 24 hours after addition of fresh medium.

siRNA knockdown and quantitative RT-PCR. $5X10^5$ cultured B cells were transfected with 2.5 nmol Accell siRNAs (Thermo Scientific) against ADAR1 and ADAR2 according to the manufacturer's protocols. 2.5 nmol of non-targeting control siRNA (NTC-siRNA) was transfected as negative controls. The cells transfected with siRNAs were incubated at 37°C in Accell transfection media for 96 hours. In time course experiment, cells were treated with another 2.5 nmol of siRNAs 24 hours following the first treatment. Accell media was then replaced with regular growth media. Cells were allowed to recover for 24 hours before being harvested. DNA and RNA were extracted using Qiagen DNeasy blood and tissue kit and RNeasy Mini kit with DNase treatment, respectively (Qiagen). Reverse transcription was carried out using Taqman reverse transcription reagent kit (Applied Biosystems). Gene expression was analyzed by quantitative PCR on ABI 7900HT system following manufacturer's protocol (Applied Biosystems).

Off-target effects of siRNA knockdown. In order to assess the off-target effects of siRNA knockdown, we transfected B-cells with four individual siRNA targeting different regions of *ADAR1* or pooled siRNA. Cells were harvested 96 hours post transfection, and real time RT-PCR and western blot were carried out to measure changes of *ADAR1*

at mRNA and protein levels. We found the pooled and individual siRNA reduced expression of ADAR1 similarly (Figure S2). In addition, pooling of siRNA is known to reduce off-target effects(Grimson et al., 2007). Therefore we used pooled siRNA throughout the study.

Next-generation sequencing and data analysis. Genomic DNA was extracted from cultured B-cells of GM12004 and GM12750 using DNeasy blood and tissue kit (Qiagen, Valencia, CA, USA). DNA-seq libraries were prepared and sequenced on HiSeq 2000 instrument (Illumina, San Diego, USA). Paired-end 100-nt reads were generated in order to achieve 60X and 30X coverage for GM12004 and GM12750, respectively. DNA-seq data were aligned to human genome (hg18) using CASAVA and BAM files generated. To select sites for further consideration: we identified those that 1) are covered by 10 or more reads, 2) have only one type of nucleotide in all reads (homozygous sites).

For transcriptome sequencing, total RNA was extracted using the RNeasy Mini kit with DNase treatment (Qiagen). RNA-seq libraries were prepared following Illumina TruSeq RNA sample preparation protocol or directional mRNA-Seq sample preparation protocol, respectively. The samples were sequenced using HiSeq 2000 instrument and 100-200 million 100-nt reads per sample were generated. Low quality bases at the 3' ends of reads defined by Illumina were trimmed, and the resulting reads were aligned to the human reference genome (hg18) using GSNAP (version 2011-03-28.v3) (Wu and Nacu, 2010) using the following parameters: Mismatches ≤[(read length+2)/12-2]; Mapping score ≥20; Soft-clipping on (-trim-mismatch-score=-3); Known exon-exon junctions (defined by RefSeq (downloaded March 7, 2011) and Gencode (version 3c))

and novel junctions (defined by GSNAP) were used. SNP sites in the CEU population from Hapmap (release #28) and 1000 Genomes (pilot project) were included for SNP-tolerant alignments. Only reads that aligned to one genomic location (uniquely mapped reads) were used in further analyses.

Expression levels of RNA transcripts were analyzed using Cufflinks version 2.1.1. using default prarmeters (Trapnell et al., 2010). We require RPKM>0.1 in at least 3 out of 4 samples (ADAR1-siRNA or negative control siRNA in B-cells from two individuals). Gene ontology analysis was carried out on DAVID Bioinformatics Resources 6.7 platform, based on a modified fisher exact test (Huang et al., 2009a, 2009b)

RNA-DNA differences. To identify RDDs, we compared RNA sequence to its corresponding DNA sequence. To be included as RDD sites in the final lists, the following criteria have to be met: 1) a minimum of 10 total RNA-seq reads covering that site in both individuals; 2) a minimum of 10 total DNA-seq reads covering that site in both individuals; 3) DNA sequence at this site is 100% concordant, without any DNA-seq reads containing alternate alleles; 4) level of RDD (# of RNA-seq reads containing non-DNA allele/# all RNA-seq reads covering a given site) is ≥10% (a minimum of two RNA-seq reads containing RDD) in at least one individual; 4) the RDD site is covered by stranded RNA-seq reads in directional RNA sequencing data, which defines strand specificity of RDD at this site; 5) RDD event is found in both individuals.

To ensure the accuracy of the RDD sites, additional filtering steps were performed using two additional mapping algorithms. First, we removed all the sites that reside in genome regions annotated as “pseudogene” in RefSeq. Second, local sequences around each

RDD site were aligned to the human reference genome to rule out misalignments to paralogous sequences or remaining pseudogenes. Specifically, for each RDD event, genomic sequences comprising sequences of length 25 bp, 50 bp, and 75 bp upstream and downstream of each site along with either the DNA variant or RNA variant were aligned to the human reference genome (hg18) using BLAT (v. 34x11). The settings '-stepSize=5' and 'repMatch=2253' were used to increase sensitivity. RDD events were removed if any of the 6 corresponding sequences aligned to another genomic location with ≤3 mismatches and with sequences that explain the RDD call (that is, if the genomic sequences match the RNA sequence). Lastly, to avoid potential misalignment of spliced reads in GSNAP due to its high gap penalty algorithm, we re-aligned all the RNA-seq reads that contain putative RDD alleles using BLAT. Human genome sequences in hg19 were included in our index in addition to sequences in hg18. Here, a low gap penalty was applied during BLAT alignment in order to compensate for high gap penalty of GSNAP alignment of spliced reads. Only RDD sites that are supported by both GSNAP and BLAT are retained for downstream analysis.

Validation of RDD using Sanger Sequencing and droplet digital PCR. To validate RDD using Sanger Sequencing, sequences surrounding RDD sites were PCR amplified using genomic DNA or cDNA as template, respectively. PCR products were sequenced using BigDye Terminator Cycle Sequencing on a 3730 DNA Analyzer (Applied Biosystems) and the results were analyzed by visual inspection. The 3'UTR region of ATM was amplified from cDNA of B-cells and cloned into TOPO vector (Invitrogen). Individual clones were sequenced, and 137 clones provided informative sequence information for downstream analysis. For sites within repetitive Alu regions, we carried

out nested-PCR with external primer annealing to non-repetitive sequences. Primers used for PCR were listed in Table S10.

For droplet digital PCR, DNA probes specific to the DNA and RNA variants at RDD sites were synthesized and labeled by VIC and FAM, respectively (ABI Biosystems, USA). PCR reaction was prepared using genomic DNA or cDNA from our subjects, VIC- and FAM- probes and Taqman reagents. Emulsion PCR was carried out following manufacture's protocol (Bio-Rad Laboratories, USA). Fluorescent signal representing each variant was quantified utilizing QuantaLife Droplet Reader (Bio-Rad Laboratories, USA). Primers and probes used in this assay are listed in Table S10.

Immunoprecipitation and western blot. Cells were lysed in Lysis buffer (20 mM Tris HCl (pH 8), 137 mM NaCl, 10% glycerol, 1% Nonidet P-40 (NP-40) and 2 mM EDTA) supplemented with 1XComplete protease inhibitors (Roche) and 1Xphosphatase inhibitors II and III (Sigma). Cell lysates containing 150 mg of total protein were incubated with 1 µg of anti-ADAR1 (#HPA003890, Sigma), or negative control rabbit IgG (ab46540, ABCAM) at 4°C overnight. Immuno-complex was pulled down using Protein A agarose (Roche), and washed 4 times with lysis buffer. Immunoprecipitate was eluted in 20 mM Tris/7.5, 150 mM NaCl, 2.5 mM MgCl2, 0.2% SDS at 30°C for 1 hour. In order to examine RNA-dependent interactions, whole cell lysates were diluted to 1 ug/ul, RNase A (Epicenter) was added to a final concentration of 0.1 ug/ul or RNase V1 (Ambion) was added to a final concentration of 0.002U/ul, and lysates were incubated at room temperature for 15 minutes. Protein samples were analyzed by western blot using anti-ADAR antibody or anti-tubulin (#05-661, Millipore), anti-EIF2AK2 (#sc-136038, Santa Cruz), anti-ILF3 (#SAB1406034, Sigma), anti-HuR (#ab54987,

ABCAM) antibodies. RNA pull-down experiment was carried out following manufactures' manual (Pierce, #20164). Primers used for amplifying DNA regions for *in vitro* synthesis of RNA transcripts are listed in Table S10.

Immunofluoresence. Primary fibroblasts were cultured at 37°C, 5% CO_2 on 1.5-thickness glass coverslips in 6-well culture plates to ~40% confluence. Cells were washed in 1xPBS and fixed in 4% paraformaldehyde in PBS for 15 minutes at room temperature. Cells were permeabilized and blocked in blocking buffer (1xPBS, 5% normal goat serum, and 0.3% Triton X-100) with gentle shaking for one hour at room temperature. Cells were then incubated with primary antibodies in antibody dilution buffer (1xPBS, 10% bovine serum albumin, 0.3% Triton X-100) at 4°C with gentle shaking overnight. Mouse anti-ADAR antibody (#ab88574, Abcam) was diluted to 10ug/ml and rabbit anti-EIF2AK2 (#3073, Cell Signaling) was diluted to 5ug/ml. Following overnight incubation, cells were washed in 1xPBS and incubated with secondary antibodies for 2 hours at room temperature with gentle shaking. The secondary antibody cocktail comprised of 2ug/ml of anti-mouse IgG with Alexa Fluor 488 conjugate (#4408, Cell Signaling) and 2ug/ml of anti-rabbit IgG with Alexa Fluor 555 conjugate (#4413, Cell Signaling). Cells were then immersed in 10ug/ml of Hoechst 33342 (#H3570, Life Technologies) in water for 30 seconds for nuclear staining. Cells were washed in 1xPBS, mounted with 12ul of VECTAshield mounting medium (#H-1000, Vector Labs), and affixed to glass microscope slides with clear nail polish. Standard epifluorescence visualization was performed on a Leica Microsystems DMI6000B with a DFC360FX camera using an HCX PL APO 40X / 0.75 Dry objective with a 1.6X magnification changer and analyzed with Leica Microsystems LAS AF6000

software. Confocal fluorescence visualization was performed on a Zeiss LSM 710 AxioObserver using a Plan-Apochromat 63X/1.4 Oil DIC M27 objective and analyzed with ZEN lite 2011 software. Shown is [z-position 7 out of 15] or [orthogonal view].

RNA-immunoprecipitation. Anti-ADAR1 and anti-HuR RNA-immunoprecipitation was carried out using Magna RNA-Binding Protein Immunoprecipitation Kit (Millipore) following manufacturer's protocol. Briefly, for each immunoprecipitation reaction, $2X10^7$ cultured B-cells were harvested and lysed in 100 µl of Lysis Buffer with protease and RNase inhibitors. 1 µg of anti-ADAR antibody (#HPA003890, Sigma), anti-HuR antibody (#sc-20694 AC, Santa Cruz), or negative control rabbit IgG (ab46540, ABCAM) were conjugated to Protein A/G beads. 100 µl of cell lysate was added into 900µl Immunoprecipitation Buffer with RNase inhibitor and incubated with 50 µl beads-antibody complex at 4°C overnight. Beads-bound immunoprecipitates were then washed six times using cold Wash Buffer with RNase inhibitor, and incubated with protease K in presence of 1% SDS at 55°C for 30 minutes. RNA was then extracted from supernatants using phenol:chloroform:isoamyl alcohol and precipitated using ethanol, followed by DNase digestion (DNA-free kit, Ambion). cDNA was synthesized using random hexamer primer by Taqman Reverse Transcription Reagent kit (Applied Biosystems). PCR and RNA-seq were carried out as described above. RNA-editing sites that were detected in transcripts pulled down by ADAR1 antibody but not by negative control IgG were considered as ADAR1-specific targets and retained for further analysis.

Overlap between editing sites identified in this and previous studies

In this study, we identified 64,931 A-to-G editing sites, from mRNA and other RNA species. We compared our results to the editing sites catalogued in the DARNED database and to those from recent studies (Bahn et al., 2011; Carmi et al., 2011; Kiran and Baranov, 2010; Li et al., 2009; Peng et al., 2012). There are 7,452 sites (11.5% of all editing sites identified in this study) that overlap between this and other studies (Figure S4B). Fifty-seven percent of the overlaps are those between the editing sites identified here and the study by Peng and colleagues. The larger overlap between these two studies is likely because the same cell type (cultured B-cells) was used for analyses. Since most of our sites were identified from analyzing products of ADAR RNA-IP which include different types of RNA (including immature and unprocessed RNA) whereas other studies mostly focused on processed mRNA, the small overlap between our and other studies is not unexpected. In addition to comparing the editing sites, we compared the genes that were edited and found that 3,306 genes (24.8% of all edited genes identified in this study) overlap between ours and previous studies. Others also found only modest overlap of editing sites among studies (Osenberg et al., 2009). This trend suggests that there are many editing sites and for most sites the editing levels are low; thus, each study samples only a different subset of these sites. A comprehensive catalogue of editing sites will require very deep nucleic acid sequencing.

Supplemental References

Bahn, J.H., Lee, J.-H., Li, G., Greer, C., Peng, G., and Xiao, X. (2011). Accurate identification of A-to-I RNA editing in human by transcriptome sequencing. Genome Research.

Carmi, S., Borukhov, I., and Levanon, E.Y. (2011). Identification of Widespread Ultra-Edited Human RNAs. PLoS Genet *7*, e1002317.

Grimson, A., Farh, K.K.-H., Johnston, W.K., Garrett-Engele, P., Lim, L.P., and Bartel, D.P. (2007). MicroRNA targeting specificity in mammals: determinants beyond seed pairing. Mol. Cell *27*, 91–105.

Huang, D.W., Sherman, B.T., and Lempicki, R.A. (2009a). Systematic and integrative analysis of large gene lists using DAVID bioinformatics resources. Nat Protoc *4*, 44–57.

Huang, D.W., Sherman, B.T., and Lempicki, R.A. (2009b). Bioinformatics enrichment tools: paths toward the comprehensive functional analysis of large gene lists. Nucleic Acids Res. *37*, 1–13.

Kiran, A., and Baranov, P.V. (2010). DARNED: a DAtabase of RNa EDiting in humans. Bioinformatics *26*, 1772 –1776.

Li, J.B., Levanon, E.Y., Yoon, J.-K., Aach, J., Xie, B., Leproust, E., Zhang, K., Gao, Y., and Church, G.M. (2009). Genome-wide identification of human RNA editing sites by parallel DNA capturing and sequencing. Science *324*, 1210–1213.

Osenberg, S., Dominissini, D., Rechavi, G., and Eisenberg, E. (2009). Widespread cleavage of A-to-I hyperediting substrates. RNA *15*, 1632–1639.

Peng, Z., Cheng, Y., Tan, B.C.-M., Kang, L., Tian, Z., Zhu, Y., Zhang, W., Liang, Y., Hu, X., Tan, X., et al. (2012). Comprehensive analysis of RNA-Seq data reveals extensive RNA editing in a human transcriptome. Nature Biotechnology.

Trapnell, C., Williams, B.A., Pertea, G., Mortazavi, A., Kwan, G., van Baren, M.J., Salzberg, S.L., Wold, B.J., and Pachter, L. (2010). Transcript assembly and quantification by RNA-Seq reveals unannotated transcripts and isoform switching during cell differentiation. Nature Biotechnology *28*, 511–515.

Wu, T.D., and Nacu, S. (2010). Fast and SNP-tolerant detection of complex variants and splicing in short reads. Bioinformatics *26*, 873–881.
