# Supplementary material for: ADAR Regulates RNA Editing, Transcript Stability, and Gene Expression
Source: Cell Rep. Author manuscript; Available in PMC 2014 Nov 14. (PMC3935819; doi:10.1016/j.celrep.2013.10.002)
Supplement: 8 [file NIHMS543889-supplement-8.pdf]

Cell Reports
Resource

Cell PRESS
Open ACCESS

ADAR Regulates RNA Editing, Transcript Stability, and Gene Expression

Isabel X. Wang,[2,4,*] Elizabeth So,[2] James L. Devlin,[2] Yue Zhao,[1] Ming Wu,[1,4] and Vivian G. Cheung[1,2,3,4,*]
[1]Howard Hughes Medical Institute, Chevy Chase, MD 20815, USA
[2]Department of Genetics, University of Pennsylvania School of Medicine, Philadelphia, PA 19104, USA
[3]Department of Pediatrics, University of Pennsylvania School of Medicine, Philadelphia, PA 19104, USA
[4]Present address: Life Sciences Institute, University of Michigan, Ann Arbor, MI 48109, USA
*Correspondence: ixwang@umich.edu (I.X.W.), vgcheung@umich.edu (V.G.C.)
http://dx.doi.org/10.1016/j.celrep.2013.10.002
This is an open-access article distributed under the terms of the Creative Commons Attribution-NonCommercial-No Derivative Works License, which permits non-commercial use, distribution, and reproduction in any medium, provided the original author and source are credited.

SUMMARY

Adenosine deaminases acting on RNA (ADARs) convert adenosine to inosine, which is then recognized as guanosine. To study the role of ADAR proteins in RNA editing and gene regulation, we sequenced and compared the DNA and RNA of human B cells. Then, we followed up the findings experimentally with siRNA knockdown and RNA and protein immunoprecipitations. The results uncovered over 60,000 A-to-G editing sites and several thousand genes whose expression levels are influenced by ADARs. Of these ADAR targets, 90% were identified. Our results also reveal that ADAR regulates transcript stability and gene expression through interaction with HuR (ELAVL1). These findings extend the role of ADAR and show that it cooperates with other RNA-processing proteins to regulate the sequence and expression of transcripts in human cells.

INTRODUCTION

Molecular studies and, more recently, genome and transcriptome sequencing have uncovered the complexity of RNA processing. From the same DNA templates, events such as RNA editing generate different forms of transcripts. In this study, we focused on ADAR-mediated RNA editing and its interactions with other RNA processing steps to regulate gene expression. In human cells, two classes of proteins are known to be involved in RNA editing: the ADAR and APOBEC families. ADARs, which are expressed in a wide variety of cell types, deaminate adenosine to inosine, which is then recognized by the translation and splicing machineries as guanosine (Bass and Weintraub, 1988; Kim et al., 1994; Rueter et al., 1995; Yang et al., 1995). APOBEC1 is expressed predominantly in human liver and converts cytidine to uridine (C-to-U) (Chen et al., 1987; Powell et al., 1987). There are only a few characterized targets of human APOBEC1, the *APOB* and *NF1* genes.

Recent work has uncovered many more RNA-editing events mediated by ADAR proteins. These findings led to new questions. Most of the A-to-G editing sites were identified by computational analysis of sequence data without experimental validation. Some of the findings were based on a comparison of RNA sequences with reference DNA sequences that were not derived from the same cells. In addition, it has been suggested that ADAR plays a role in other biological processes in an editing-independent manner (Clerzius et al., 2009; Heale et al., 2009), but the extent of these processes is not known. Lastly, it is not clear whether ADAR1 and ADAR2 play the same role or different roles in human cells. To address these issues, we sought to answer three main questions: (1) What sites do ADAR proteins edit? (2) Do ADAR proteins regulate gene expression, and if so, is this regulation dependent on editing? (3) What other proteins interact with ADARs in RNA processing?

We compared DNA and RNA sequences in human B cells from two individuals to identify RNA-DNA sequence differences (RDDs). We validated the findings by RNAi and RNA immunoprecipitation (RNA-IP). The results uncovered ~10,000 known and ~50,000 unknown ADAR-mediated A-to-G editing sites in premature and mature mRNAs and long noncoding RNAs (lncRNAs). We also found that ADAR proteins have an editing-independent effect on gene expression. Our results showed that ADAR1 interacts with HuR (ELAVL1) to regulate transcript stability. Together, these results provided us with a deeper understanding of ADAR proteins in RNA editing and gene regulation.

RESULTS

DNA and RNA Sequencing

We sequenced the DNA and mRNA from cultured B cells of two individuals using Illumina-based next-generation sequencing (NGS) (Bentley et al., 2008). We conducted DNA sequencing (DNA-seq) to >30× coverage and obtained >140 million RNA sequencing (RNA-seq) reads for each sample. At least 80% of the sequence reads mapped to the reference genome sequence (Table S1). For each individual, we compared their DNA and mRNA sequences to identify editing and other types of RDDs

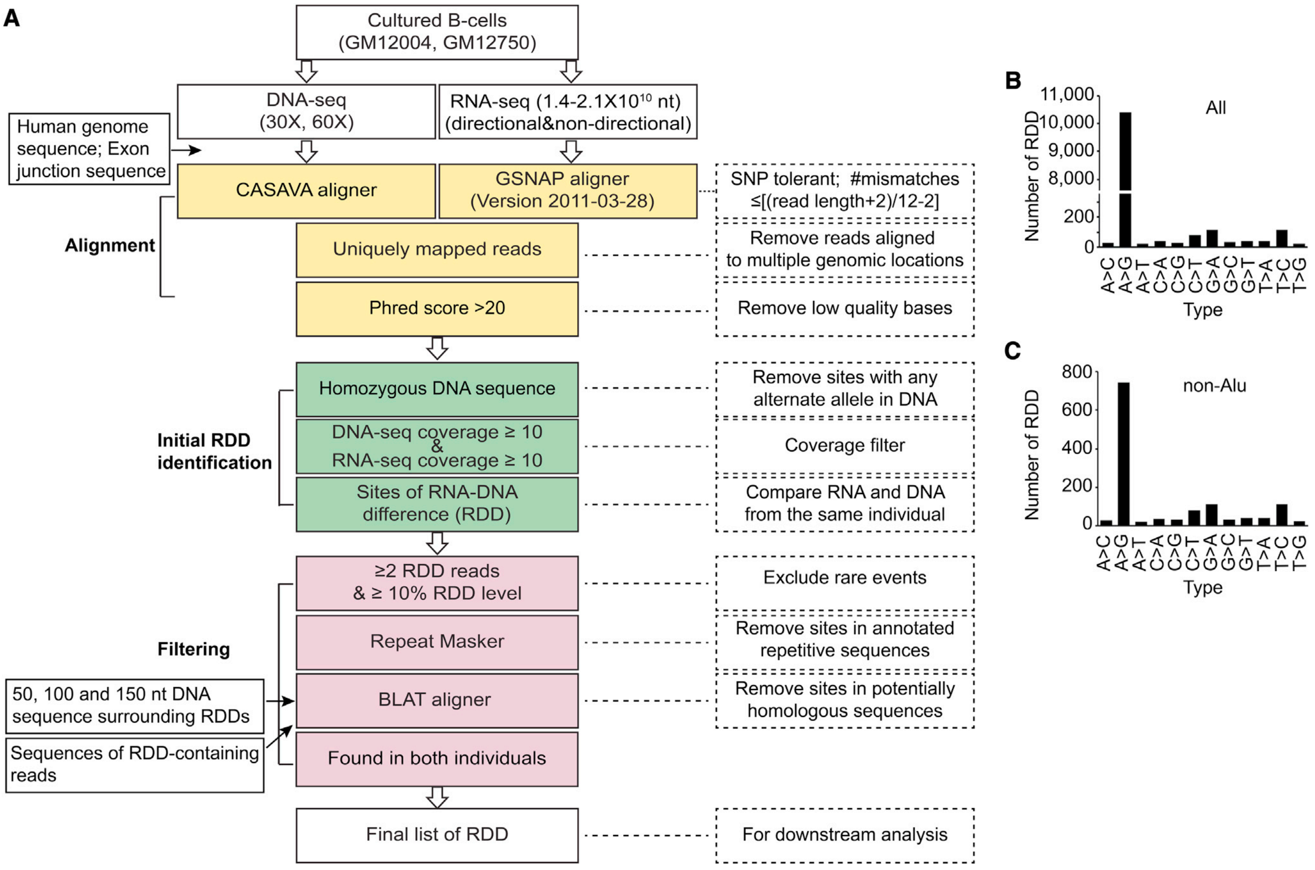

Figure 1. Identification of RDDs

(A) Analysis steps to identify RDDs (see also the Supplemental Experimental Procedures). All 12 types of RDDs were found.
(B) Sites detected genome wide.
(C) Sites detected in non-Alu regions.
See also Tables S1 and S2.

(Chen et al., 2012; Ju et al., 2011; Li et al., 2011). Data from strand-specific (directional) sequencing allowed us to annotate all 12 types of possible mismatches between DNA and RNA sequences. To simplify the mapping of the sequence reads, repetitive sequences are often excluded. However, since most of the ADAR-mediated A-to-G editing sites were found in Alu repeats (Athanasiadis et al., 2004; Kim et al., 2004; Levanon et al., 2004; Peng et al., 2012), we retained Alu sequences (but excluded other sequence repeats) in our analysis. Using stringent thresholds, we identified 10,992 sites where the RNA sequences were discordant from the corresponding DNA sequences in both individuals (Figure 1A; Table S2). All 12 types of RDDs (A-to-C, A-to-G, etc.) were found (Figure 1B). These included 9,675 sites in Alu-containing regions and 1,317 sites in nonrepetitive regions of the genome. The distributions of the 12 types of RDDs were very different for Alu-containing and Alu-free regions of the genome. Most (99%) of the sites in Alu regions were A-to-G editing sites, whereas in regions without Alu repeats, only 57% were A-to-G sites (Figure 1C). We then validated the results by Sanger sequencing and emulsion-based droplet digital PCR (Figures 2 and S1). Twenty-four out of 25 sites were validated by Sanger sequencing, and five out of six sites were validated by droplet digital PCR. Thus, the false discovery rate (FDR) is approximately 6.5% (Figure S1).

ADAR1 Plays a Major Role in A-to-G RNA Editing in Human B Cells

To assess the extent to which the ADAR family of deaminases contributes to mismatches between RNA and corresponding DNA sequences, we carried out RNAi-mediated gene knockdowns and deep sequencing of the resulting cells. Human B cells possess three members of the ADAR family: ADAR1, ADAR2, and ADAR3. ADAR1 and ADAR2 are functional deaminases (Bass and Weintraub, 1988; Kim et al., 1994), whereas ADAR3 does not have a known enzymatic function (Chen et al., 2000). The expression level of *ADAR1* is >20 times higher than that of *ADAR2* and *ADAR3* (reads per kilobase of transcript per million mapped reads [RPKM] of *ADAR1* = 7 compared with RPKM of *ADAR2* and *ADAR3* < 0.3), suggesting that ADAR1 is the predominant form of ADARs in human B cells. Following gene knockdown with four independent siRNAs and a pool comprising the four siRNAs, *ADAR1* was reduced by >50% at

Cell PRESS
Open ACCESS

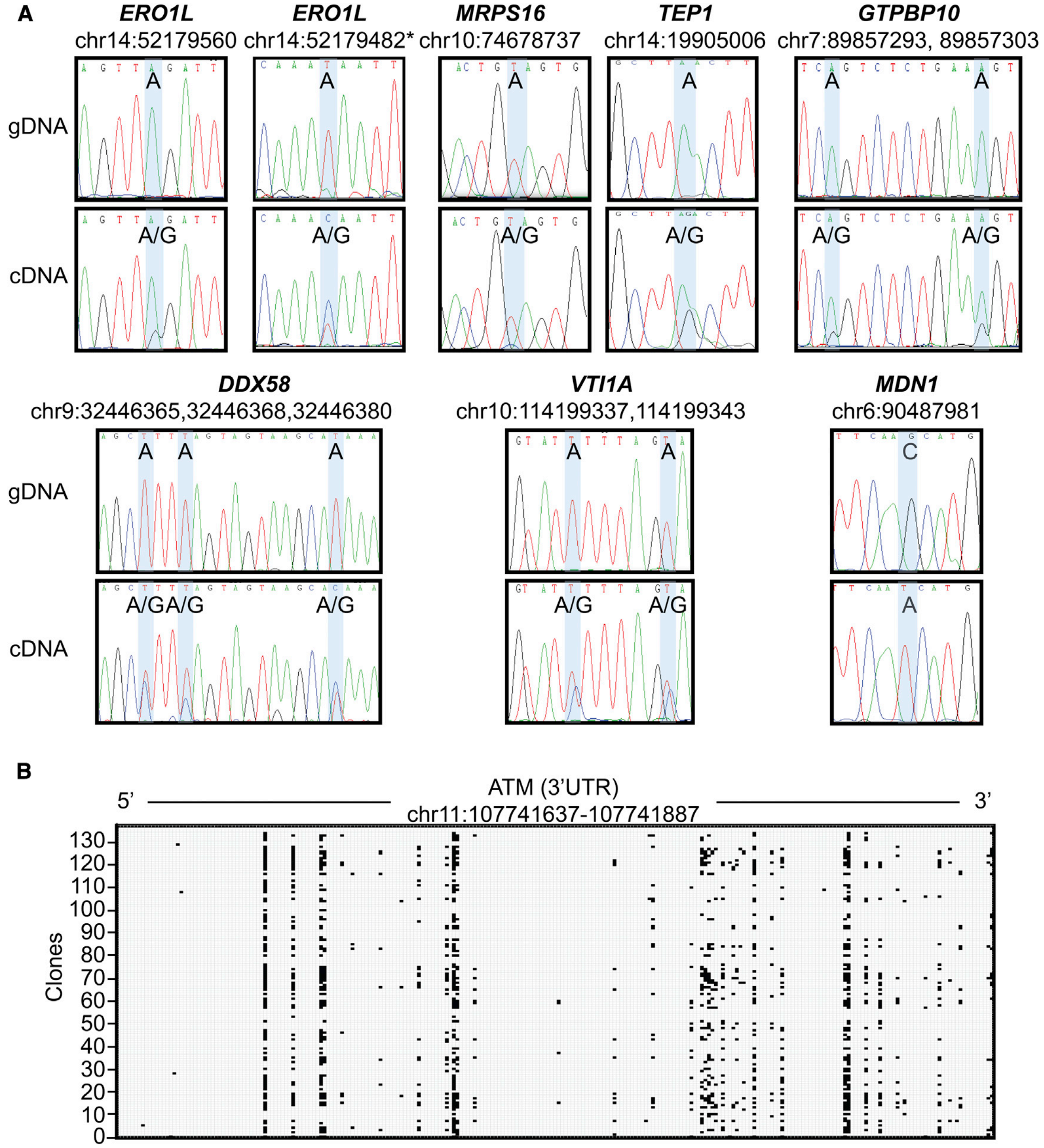

Figure 2. Validation of A-to-G Editing and RDD Sites by Sanger Sequencing

(A) Sequences surrounding editing or RDD sites were amplified by PCR using genomic DNA or cDNA from the same two individuals as templates. The sites validated by Sanger sequencing are highlighted in blue and the corresponding nucleotide changes are labeled. Some samples were sequenced from the reverse strand, and the nucleotides are labeled according to the forward strand. *An example of an editing site in *ERO1L* that did not meet our inclusion criteria but nonetheless was validated by Sanger sequencing.

(B) Hyperedited region in *ATM* transcript. 3′ UTR of *ATM* was PCR amplified from cDNA and cloned. Sequences from 137 individual clones are illustrated. Each black dot represents an A-to-G site detected in a clone by Sanger sequencing.

See also Figure S1.

mRNA and protein levels (Figures 3A, 3B, S2A, and S2B). The editing activities were also reduced, as A-to-G editing in *EIF2AK2* mRNA, a known target of ADAR1 (Blow et al., 2004), was abolished following *ADAR1* knockdown (Figures 3C and S2C). Similar results were obtained from the different siRNAs; for subsequent experiments, we used the pooled siRNAs to minimize off-target effects (Supplemental Experimental Procedures; Figure S2; Grimson et al., 2007).

Next, we sequenced and compared the DNA and RNA of the siRNA-treated B cells. This allowed us to experimentally validate the editing sites and determine the effect of ADAR1 on editing. False-positive results due to misalignment of

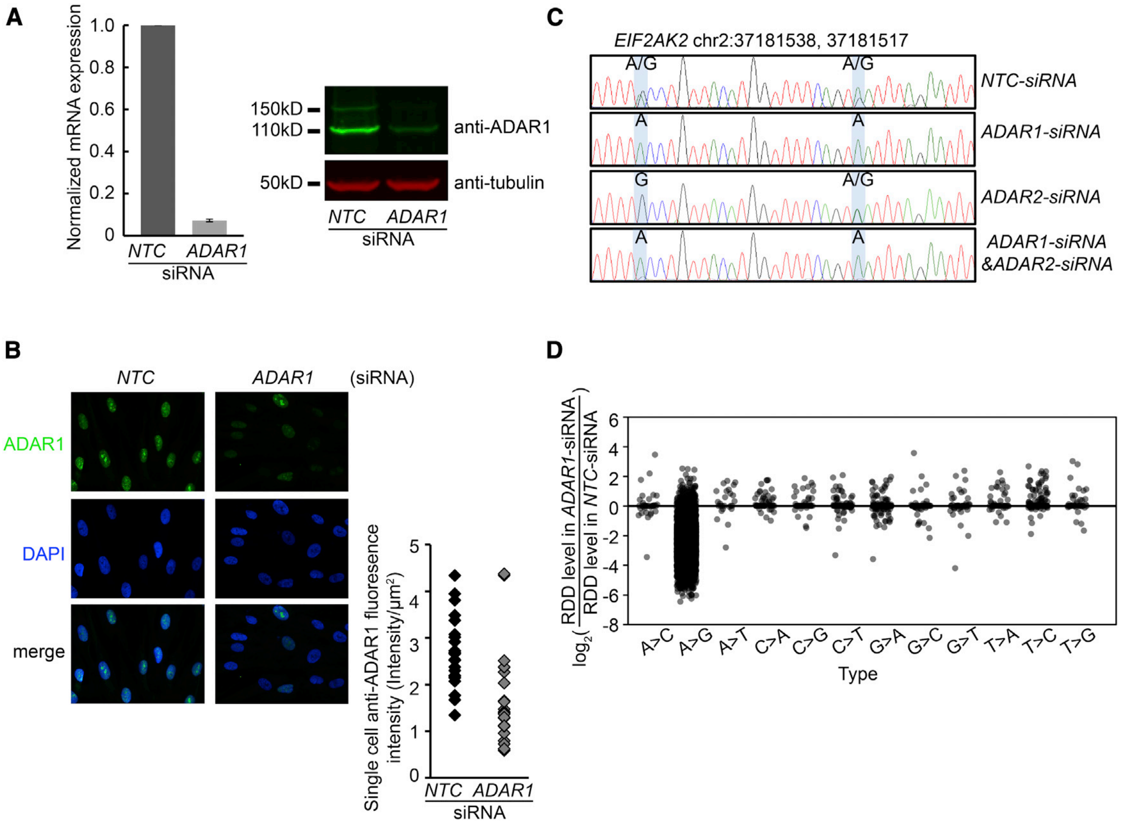

Figure 3. siRNA Knockdown of *ADAR1* Resulted in Reduced A-to-G Levels

(A) Left panel: real-time RT-PCR shows the decrease in the *ADAR1* mRNA level following knockdown using pooled siRNA. The average fold change from triplicates is shown. Error bar indicates SEM. Right panel: western blot shows the decrease of ADAR1 protein following knockdown.

(B) Immunofluorescence staining of primary fibroblast confirmed that siRNA knockdown results in a decrease of ADAR1 expression. Left panel: representative immunofluorescence image of primary fibroblasts treated with nontargeting control siRNA (NTC) or *ADAR1*-siRNA. Right panel: fluorescence quantification of ADAR1 expression in 24 cells treated with NTC-siRNA or *ADAR1*-siRNA, respectively.

(C) Editing levels at two A-to-G sites in *EIF2AK2* were reduced following *ADAR1* knockdown, but the levels increased following *ADAR2* knockdown and were abolished following double knockdown.

(D) *ADAR1* knockdown led to reduced levels in 96% A-to-G sites, but had a minimal effect on other types of RDDs.

See also Figures S2–S4 and Tables S3, S6, and S7.

sequence reads or other artifacts would not “respond” to siRNA treatments.

ADAR and RNAi pathways work cooperatively (Scadden and Smith, 2001; Wu et al., 2011; Yang et al., 2005), so the double-stranded RNAs (dsRNAs) used in gene knockdown likely have effects on ADAR function other than knockdown of its expression level. To study the specific effects of *ADAR1* knockdown, we compared the sequences of cells transfected with control siRNAs with those of cells treated with pooled *ADAR1*-specific siRNAs. In the cells treated with control siRNA, we found 6,996 sites where the RNA and DNA sequences were discordant, including 6,524 A-to-G editing sites. In the *ADAR1* knockdown cells, the editing level of 6,258 (96%) sites decreased by 20% or more in samples from both individuals, whereas only 43 sites of the other 11 types of RDDs decreased by the same extent (Figure 3D; Table S3). A small number of sites (91 of the A-to-G sites and 125 of the other RDDs) showed increased levels following *ADAR1* knockdown. The editing levels of >2,000 A-to-G sites were reduced to zero following *ADAR1* knockdown. These included sites in genes that encode caspases (*CASP8* and *CASP10*) and the von Hippel-Lindau (*VHL*) tumor suppressor, which have been implicated in various cancers. In contrast, the levels of the other types of RDDs did not change or decreased very modestly. This suggests that ADAR1 mediates the majority of A-to-G editing in B cells and does not contribute to the other types of RDDs. In addition, these results show that the FDR of A-to-G editing is no more than 4%.

The above data were obtained at one time point. In order to study the kinetics of A-to-G editing, we carried out RNA-seq on the cells at several time points after siRNA transfection. The expression level of *ADAR1* and the editing levels of its many targets remained low throughout the time course (Figures S3A and S3B). For instance, the A-to-G editing levels in *TRAF1*, *CENPH*, and *USP46* were less than 5% of those in control samples 96 hr after siRNA transfection. Gene Ontology analysis (Ashburner et al., 2000; Huang et al., 2009a, 2009b) showed that

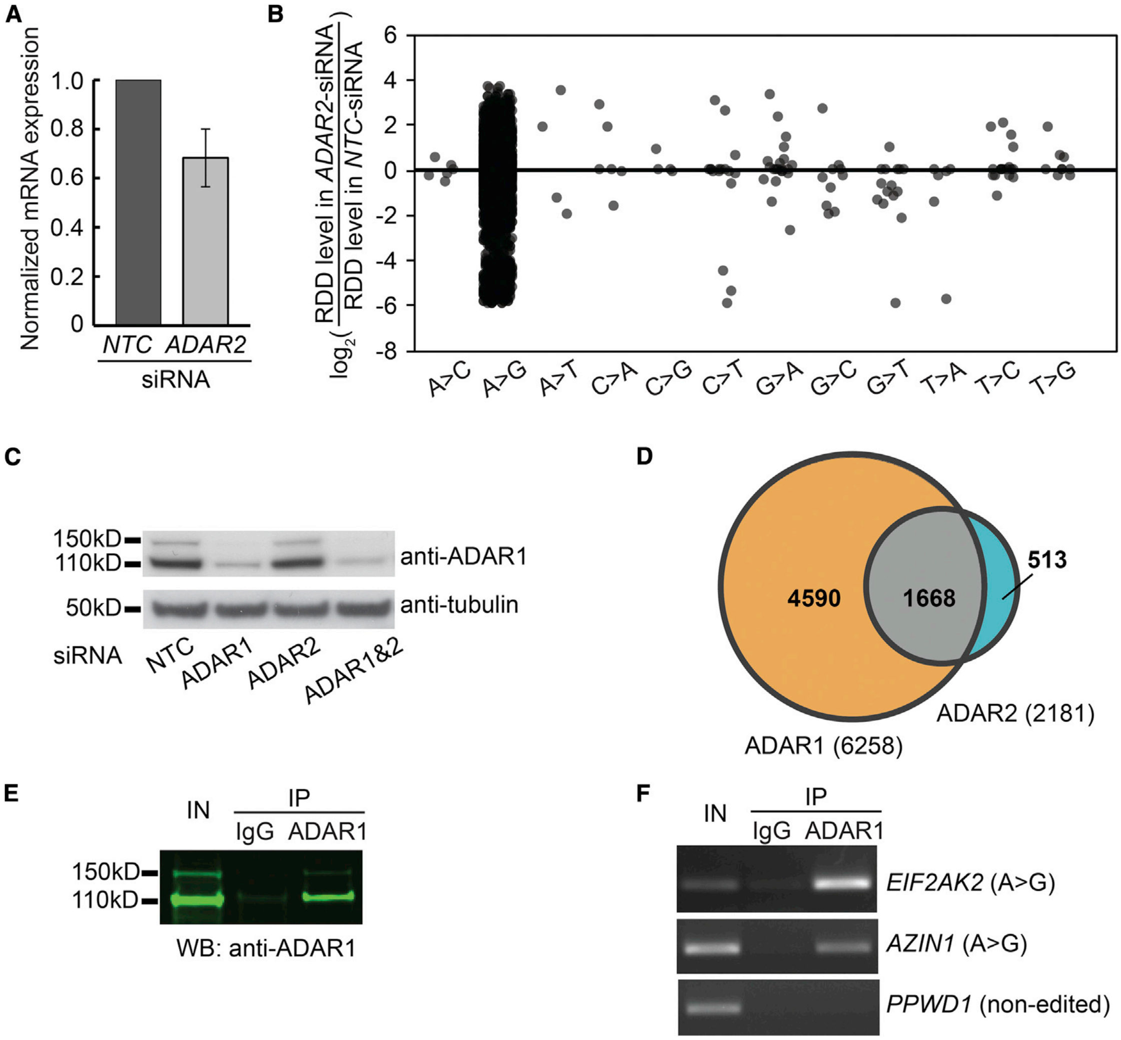

Figure 4. Role of ADAR2 in RNA Editing

(A) Real-time RT-PCR shows that the *ADAR2* mRNA level is downregulated following siRNA knockdown. We were unable to assess changes in the ADAR2 protein level because none of the antibodies we tested gave a specific ADAR2 signal in western blot. Error bar indicates SEM.

(B) *ADAR2* knockdown led to changes of editing levels in ~2,000 A-to-G sites. See also Table S4.

(C) Western blot shows that the ADAR1 protein level is not upregulated following *ADAR2* knockdown.

(D) ADAR1 targets more editing sites than ADAR2. The Venn diagram shows shared and unique editing sites targeted by ADAR1 and ADAR2.

(E) Anti-ADAR1 RNA-IP pulled down ADAR1 protein and its associated editing targets specifically. Western blot shows that anti-ADAR1 pulled down ADAR1 protein.

(F) RT-PCR shows that ADAR1 antibody pulled down transcripts of the editing targets, *EIF2AK2* and *AZIN1*, but not the negative control transcript, *PPWD1*. See also Figure S2 and Table S5.

editing targets are enriched for genes that encode zinc-finger proteins ($p < 0.05$), as well as proteins that are involved in chromosomal organization ($p < 10^{-5}$) and antiviral defense ($p < 10^{-3}$).

Role of ADAR2 in RNA Editing in Human B Cells

Next, we carried out siRNA knockdown of *ADAR2* (*ADARB1*) followed by nucleic acid sequencing. The *ADAR2* mRNA level was reduced by 25% (Figures 4A and S2A). The lack of specific antibodies prevented us from measuring ADAR2 protein expression. Following *ADAR2* knockdown, we observed a decrease in its activity: the editing levels of 2,181 of 6,084 A-to-G sites (Table S4), and 32 of the other types of RDDs decreased by at least 20%. In contrast to *ADAR1* knockdown, after *ADAR2* knockdown, the levels of 2,240 A-to-G sites increased by 20% or more (Figure 4B). We reasoned that these sites (e.g., those in *EIF2AK2*) are mainly targeted by ADAR1; therefore, following *ADAR2* knockdown, a compensatory increase in ADAR1 binding or activity would lead to higher editing levels, which would be abolished by the simultaneous silencing of *ADAR1* and *ADAR2*. This hypothesis was confirmed by a decrease in EIF2AK2 editing following double knockdown of *ADAR1* and *ADAR2* (Figure 3C). The compensation is not due to higher

Cell PRESS
Open ACCESS

Table 1. Hyperedited Transcripts

Hyperedited Region	Gene Symbol	Number of Edited Sites
chr9:131701274-131841654	*FNBP1*	291
chr3:47608175-47795690	*SMARCC1*	218
chr1:1713762-1810015	*GNB1*	214
chr4:39379937-39452078	*UBE2K*	167
chr5:138923557-138985907	*UBE2D2*	162
chr8:98728784-98810463	*MTDH*	154
chr15:42553696-42603223	*CTDSPL2*	148
chr1:149438531-149485085	*PIP5K1A*	141
chr10:70152450-70219274	*CCAR1*	138
chr17:24746313-24892874	*TAOK1*	136
chr16:68968176-69027669	*ST3GAL2*	134
chr5:176497466-176651020	*NSD1*	134
chr2:61559886-61613430	*XPO1*	133
chr3:49046623-49101020	*QRICH1*	131
chr12:49088991-49144511	*LARP4*	128
chr16:15655703-15700735	*NDE1*	128
chr1:149652924-149695116	*POGZ*	127
chr19:17076438-17180447	*MYO9B*	127
chr19:16604467-16625697	*C19orf42*	125
chr16:88337701-88409128	*FANCA*	123

ADAR1 protein expression, since it increased only minimally following *ADAR2* knockdown (Figure 4C). These results suggest that the increase in editing levels following *ADAR2* knockdown could be due to increased availability of the sites to ADAR1 and/or homodimerization of ADAR1, a more active form of ADAR1 (Chilibeck et al., 2006; Lehmann and Bass, 2000).

Shared Editing Targets of ADAR1 and ADAR2

Next, we examined the specificity of ADAR1 and ADAR2 by comparing editing sites identified from the knockdown experiments described above. We found that the editing levels of 6,771 sites decreased after at least one of the ADAR proteins was silenced. Of these, 1,668 sites showed a reduction in editing levels by $\geq$20% following knockdown of *ADAR1* and *ADAR2*, suggesting they are targets of both enzymes (Figure 4D; Tables S3 and S4). These included sites in genes that encode the DNA damage repair protein ERCC4 and the telomerase-associated protein TEP1. Other targets appeared to be specific to ADAR1 or ADAR2: 4,590 sites showed a decrease in levels following only *ADAR1* silencing, and 513 sites showed a decrease only in *ADAR2* knockdown (Figure 4D). The extent of *ADAR2* knockdown is smaller than that of *ADAR1* knockdown, which could account for the more modest decrease in A-to-G editing following *ADAR2* knockdown.

RNA-IP Uncovered Many Additional A-to-G Editing Sites

ADAR deaminases are RNA-binding proteins that interact directly with their substrates (Klaue et al., 2003). To understand the RNA-binding activity of ADAR1, we carried out native IP of ADAR1 in B cells and sequenced the RNA that coprecipitated with the ADAR1 protein (Figure 4E). Previously, we selected polyadenylated mRNAs for analysis in order to obtain adequate sequence coverage. Here, we targeted the IP to RNAs that are specifically bound to ADAR1 in vivo without selecting for polyadenylated mRNAs. This allowed us to study the effects of ADAR1 on a broader set of RNAs, including immature transcripts whose introns have yet to be spliced out. To test the quality of the ADAR RNA-IP, we showed that known ADAR1 substrates, such as *EIF2AK2* and *AZIN1*, were bound by ADAR1 protein, in contrast to the control transcript *PPWD1*, which is not edited (Figure 4F). We next carried out RNA-seq analysis and identified edited transcripts that were pulled down by ADAR1 antibody but not by negative-control immunoglobulin G (IgG). Using the same thresholds as above, we identified 55,719 A-to-G sites in the two individuals, which is far more than the 10,412 editing sites identified from the mRNA samples of the same individuals (Table S5). Transcripts that are bound and edited by ADAR1 protein include those that encode WEE1, a protein kinase that plays a role in DNA replication, and COPB1, a member of the coatomer protein complex that is involved in trafficking between the Golgi and the endoplasmic reticulum.

Of these 55,719 sites, fewer than 4,500 sites have been previously reported (Bahn et al., 2012; Carmi et al., 2011; Kiran and Baranov, 2010; Li et al., 2009; Peng et al., 2012). The majority (81%) of the sites were found in introns and some were found in lncRNAs, including *LINC00265* and *LINC00476*. The transcripts from the RNA-IP were hyperedited: >30% of the editing sites clustered in 224 transcripts, each of which had >50 A-to-G editing sites (Table 1). More than 97% of the 55,719 editing sites were in Alu repeats that promote dsRNA formation and therefore binding and hyperediting by ADAR proteins (Osenberg et al., 2009). When we examined a hyperedited region of *ATM* more closely, we found that each adenosine was deaminated. However, the editing level at a given site ranged from 1% to 99%, and within a given transcript there was no obvious pattern as to which adenosine was edited (Figure 2B).

Features Differ between A-to-G Editing Sites and Other Types of RDDs

The results from *ADAR* knockdown and RNA-IP suggest that although ADARs mediate A-to-G editing, they do not mediate other types of RDDs. The levels of other types of differences were largely unaffected by *ADAR* knockdown, and the transcripts that showed those differences were not bound by ADAR. This prompted us to compare the genomic features surrounding the A-to-G editing sites and other types of RDDs. First, the sequence contexts of A-to-G and non-A-to-G sites are different. The base 5′ adjacent to the adenosine in A-to-G sites is depleted of guanosine (G) and the base 3′ to A-to-G editing sites is enriched for G (Figure 5A), consistent with previous reports (Lehmann and Bass, 2000). This sequence feature is specific to A-to-G editing because it is not present in random adenosines within nonedited Alu repeats (data not shown). This sequence motif was also not found for any of the RDDs. We identified sequence motifs for G-to-A and T-to-C sites, and they differed from the motif around the A-to-G sites (Figure 5A). Second, the A-to-G sites were more clustered than the non-A-to-G sites (67% of A-to-G sites were found within 25 nt of each

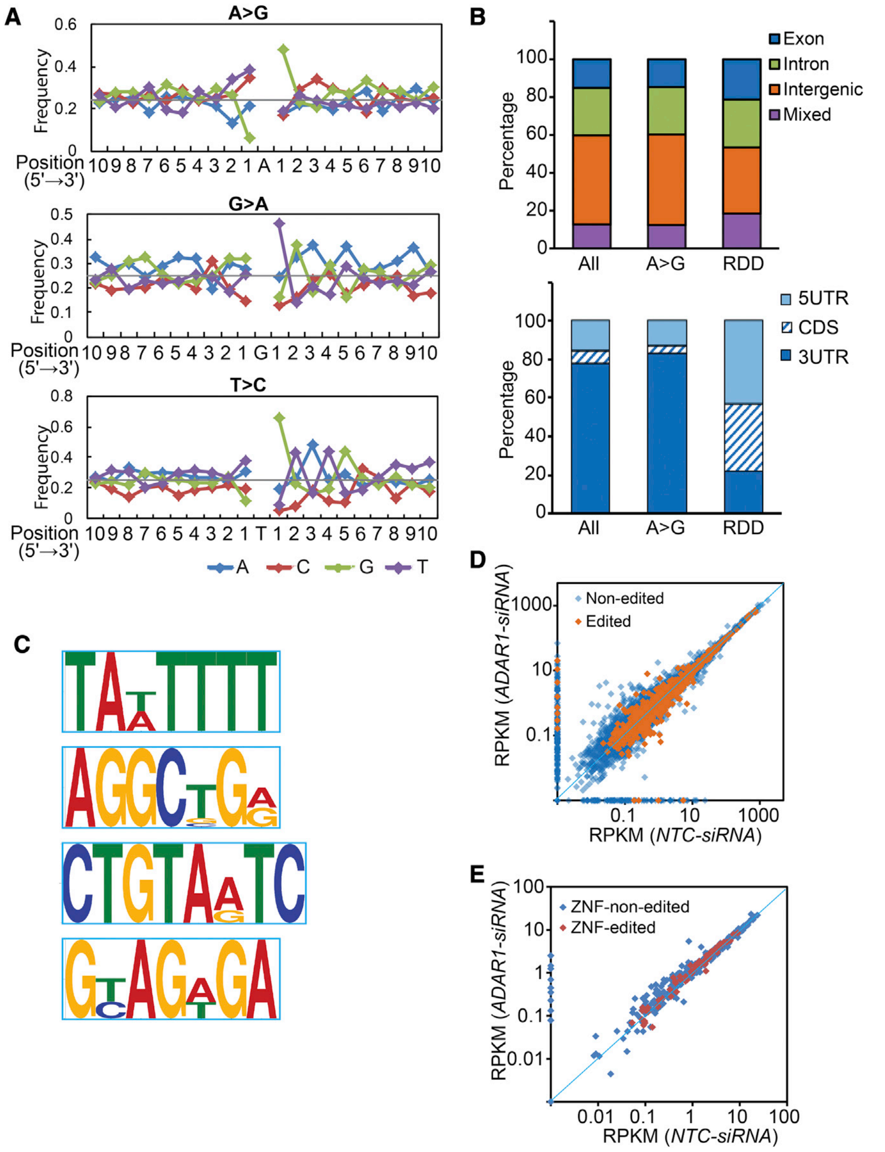

Figure 5. Features of A-to-G and RDD Sites

(A) The nucleotide 5′ to A-to-G sites is depleted of G, and the nucleotide 3′ to A-to-G sites is enriched for G. In contrast, the nucleotide 3′ to G-to-A sites is enriched for T, and the nucleotide 3′ to T-to-C sites is enriched for G. Sequences for 10 nt upstream and downstream of A-to-G or RDD sites were analyzed and the frequencies of A, C, T, and G at each position are shown. The horizontal line at a frequency of 0.25 indicates the expected frequency if the four nucleotides are represented equally.

(B) A-to-G and other RDD sites are found in different genomic regions. Upper panel: genome-wide distribution ("Mixed" indicates regions with multiple or ambiguous annotation). Lower panel: distribution in exonic regions.

(C) Sequence motifs for editing targets pulled down in anti-ADAR RNA-IP assays. The MEME program was used to analyze DNA sequences corresponding to 100 nt upstream and downstream of editing sites. The four motifs that are most significantly enriched in input sequences are shown ($p < 10^{-10}$, Fisher's exact test). Scrambled sequences were used as negative-control sequences.

(D and E) Expression levels of transcripts do not correlate with editing levels. RPKM values of transcripts measured in an *ADAR1* knockdown sample and a negative-control sample (NTC) are plotted. Edited and nonedited transcripts are indicated in different colors.

(D) All transcripts.

(E) Genes encoding zinc-finger proteins whose expression levels changed by ≥20%.

See also Table S8.

other, compared with 14% of non-A-to-G RDDs). Third, most of the A-to-G sites were within or near inverted repeats, which form dsRNA and are preferentially recognized and bound by ADAR enzymes. Nearly 45% of the A-to-G sites resided within inverted repeats and another 30% were found near inverted repeats (<1 kb). In contrast, very few (0.9%) of the non-A-to-G sites were found in inverted repeats. Lastly, A-to-G sites and RDD sites were found in different regions of genes. A-to-G sites were found mostly in the 3′ UTRs, whereas RDDs were found mainly in the 5′ UTRs and in coding exons. Only 4% of the A-to-G sites (compared with 35% of RDDs) were in coding exons (Figure 5B). The differences between A-to-G editing sites and the other types of RDDs suggest that they are mediated by different mechanisms. Biochemically, this is expected since some of the RDDs are transversion events that cannot be explained simply by deamination.

Sequence Motifs near A-to-G Editing Sites

The large number of RNA editing sites in our study gave us an opportunity to uncover characteristics of the editing targets. We expanded our sequence analysis to 100 nt upstream and downstream of A-to-G sites using the motif discovery tool MEME (Bailey et al., 2009). MEME identified four motifs that are significantly enriched in the sequences surrounding the A-to-G editing sites compared with control sequences ($p < 10^{-10}$, Fisher's exact test; Figure 5C). One of these motifs (TA(T/A)TTTT) corresponds to the binding motif of HuR, an RNA-binding protein that regulates mRNA turnover (Myer et al., 1997). Other studies have also investigated the sequence and structure specificity of targeted sites of ADAR enzymes (Bahn et al., 2012; Daniel et al., 2012; Dawson et al., 2004; Kuttan and Bass, 2012; Lehmann and Bass, 2000; Wong et al., 2001). However, the sequence motifs we described here have not been previously reported in ADAR editing targets. This is likely because we searched more distant sequences surrounding editing targets in a larger number of editing sites of various types of RNAs, whereas most previous studies focused on immediately adjacent sequences on fewer targets.

Finding the HuR motif near ADAR-binding sites led us to reason that ADAR interacts with other RNA-binding proteins. The sequence motifs for RNA-binding proteins in edited transcripts suggest cooperative binding among RNA processing proteins, akin to the coupling seen in regulation of gene expression by multiple transcription factors. This finding prompted us to study the interactions between ADAR1 and HuR proteins (see below).

ADAR Regulates Gene Expression

After examining how ADAR proteins affect RNA sequences, we turned to study their effects on gene expression and to determine the relationship between RNA editing and gene expression. We found that ADAR1 and ADAR2 affect the expression of thousands of genes and their transcripts in human B cells. We looked for genes that showed changes in the total gene-expression level. Following *ADAR1* knockdown, 635 genes showed significant changes in gene expression in two individuals ($p < 0.05$; Table S6). The RNA-seq data allowed us to analyze the effect of ADAR on gene expression at single-nucleotide resolution to quantify changes of transcript expression in addition to total gene expression following *ADAR1* knockdown. Many genes demonstrate "isoform switching" under physiological or experimental perturbations (Trapnell et al., 2013). The expression levels of 1,238 transcripts showed significant changes in expression (Table S6). Nearly half of these transcripts (579) belong to the genes that changed the total expression level. However, changes in 659 transcripts were not reflected at the total gene-expression level. For some transcripts, such as *VNN2* and *ARHGAP19*, two isoforms showed changes in opposite directions, and thus the total gene levels that are the sums of isoforms did not show change (Figure S3C). Gene Ontology analysis (Huang et al., 2009a) showed that these ADAR-regulated genes are enriched in kinase ($p < 10^{-9}$), DNA damage response proteins ($p < 10^{-10}$), and zinc-finger proteins ($p < 10^{-6}$; Table S7).

RNA-seq data provide information on editing and gene expression in the same samples, and thus allow us to assess the connection between the two. We examined the levels of ADAR1-dependent editing and transcript expression, and found that they were not correlated ($r < 0.05$ for both individuals). Following *ADAR1* knockdown, changes in expression level were independent of the editing status of the target genes (Figure 5D). For example, among the 263 zinc-finger protein genes whose expression levels changed following *ADAR1* knockdown, only 40% (104 genes) were editing targets of ADAR1. ADAR1 regulated the expression of zinc-finger proteins regardless of whether they were editing targets or not (Figure 5E). For instance, the expression levels of *ZNF16* decreased and those of *ZNF432* increased following *ADAR1* knockdown; however, even though they both had multiple Alu repeats, neither gene was edited. Therefore, editing of Alu is not required for ADAR1 to regulate the expression of zinc-finger proteins (Shen et al., 2011).

Another way to investigate the relationship between RNA editing and gene-expression regulation is to study the 106 genes that are both edited and regulated by ADAR1 at the mRNA expression level (Table S8). Among these, following *ADAR1* knockdown, the expression levels of 67 genes increased and those of 39 genes decreased. Changes in editing levels and gene expression following *ADAR1* knockdown were not significantly correlated ($r < 0.05$). For example, *IKZF3*, a transcription factor that regulates proliferation and differentiation of B lymphocytes, has 68 A-to-G editing sites. Its expression level increased by 1.3-fold, whereas its editing level decreased by >7-fold following *ADAR1* knockdown. In contrast, both the editing and expression levels of *CENPN* (43 A-to-G editing sites) decreased following *ADAR1* knockdown. The positions of edited sites within genes (such as coding exons, 3′ UTRs) and the number of edited sites per transcript also did not correlate with changes in expression following *ADAR1* knockdown. These results further suggest that ADAR1 can affect gene expression independently of its deamination activity.

We also examined the editing and gene-expression regulatory roles of ADAR2. Although ADAR2 has fewer editing targets than ADAR1, it regulates the expression levels of more genes. Following *ADAR2* knockdown, the expression levels of 4,154 transcripts (in 3,379 genes) increased by 2-fold, and those of 872 transcripts (in 734 genes) decreased by 2-fold (Table S9). Thus, ADAR2 has a broader effect on gene expression even though it plays a lesser role in editing compared with ADAR1. This further implies that ADAR proteins affect editing and gene expression independently.

ADAR1 Interacts with HuR to Regulate Transcript Stability

Our analysis of sequence motifs around editing sites identified an enrichment of HuR-binding motifs. This motivated us to study whether HuR and ADAR1 function cooperatively. HuR binds to single-stranded RNA (ssRNA) and regulates transcript stability and gene expression (Fan and Steitz, 1998). We carried out protein IP using anti-ADAR1 and negative-control IgG. We confirmed specific pull-down of ADAR1 by immunoblotting, and identification of transcripts and protein of EIF2AK2, a known editing target and interacting partner of ADAR, in the immunoprecipitates (Figure 6A; Clerzius et al., 2009). Using antibody against HuR, we found that HuR was pulled down with ADAR1, suggesting these two proteins interact in vivo (Figure 6B, lanes 4 and 5). As a control, ILF3, a protein that is known to interact with ADAR1 in a dsRNA-dependent manner, was also pulled down (Nie et al., 2005). Next, we asked whether the interaction between ADAR1 and HuR is dependent on scaffold RNAs. We carried out ADAR1-IP using RNase A- and RNase V1-treated whole-cell lysates. RNase A treatment, which digests ssRNA, abolished the interactions between HuR and ADAR1, but not the interactions between ILF3 and ADAR1. In contrast, the dsRNA-specific RNase V1 reduced the interactions between HuR and ADAR1, and between ILF3 and ADAR1 (Figure 6B, lanes 6–9). These results show that the interaction between HuR and ADAR1 is dependent on both ssRNA and dsRNA.

To examine how ADAR and HuR interact with their RNA targets, we carried out additional analyses. First, we studied the HuR-binding sites in ADAR-bound transcripts. As mentioned above, we found that sequences of transcripts bound by ADARs were enriched for AU-rich elements (AREs), which are HuR-binding sites. Among the 4,279 ADAR-bound transcripts, 4,198 (98%) had at least one and often many HuR-binding sites. There were 172,000 TA(T/A)TTTT sites in ADAR-bound transcripts,

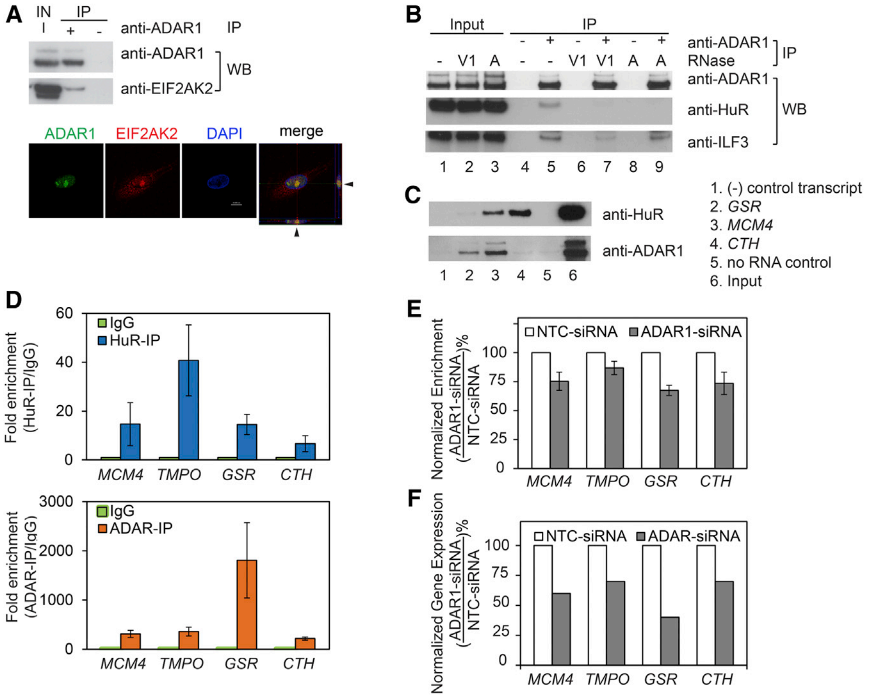

Figure 6. ADAR1 and HuR Proteins Interact in an RNA-Dependent Manner and Coregulate Common Transcripts

(A) Anti-ADAR1-IP of ADAR1 and its interacting protein EIF2AK2. Western blot analysis shows that ADAR1 and EIF2AK2 are pulled down by anti-ADAR1, but not by negative controls. Confocal immunofluorescence analysis confirms the interaction between ADAR1 and EIF2AK2 in the nucleus. Arrows indicate orthogonal views of colocalized ADAR1 and EIF2AK2.

(B) ADAR1 and HuR interact in vivo in an RNA-dependent manner. RNase A and V1 treatment before IP abolishes the interaction between ADAR1 and HuR.

(C) RNA pull-down experiments showed that HuR (top panel) and ADAR (bottom panel) bind to the same target transcripts. A $(polyA)_{25}$ RNA was used as the negative-control transcript. Cell lysate incubated with mock solution before pull-down was included as the no-RNA control.

(D) ADAR1 and HuR antibodies, but not control IgG, pulled down the same transcripts. Following anti-ADAR1 and anti-HuR RNA-IP, quantitative RT-PCR was carried out to measure the levels of various transcripts. RNA levels bound by negative-control IgG were normalized to one.

(E) *ADAR1* knockdown leads to reduced binding of HuR to their target transcripts. HuR RNA-IP was carried out in cells treated with ADAR1-siRNA or NTC-siRNA, and the HuR-associated transcript level was measured by quantitative RT-PCR.

(F) The gene expression of the target transcripts of HuR and ADAR1 was reduced following *ADAR1* knockdown. Gene expression levels from RNA-seq data (RPKM) were normalized to those obtained from NTC-siRNA samples.

Error bar indicates SEM. See also Tables S2 and S4.

significantly more (χ^2, p < 0.0001) than in control transcripts (68% of 4,279 random control transcripts contain 79,084 AREs). Similarly, other HuR-binding sequences (including (U/A) UUUA, (U/C)UUUA, and AUUU(U/C); Mukherjee et al., 2011) were also enriched in ADAR-bound transcripts. Second, since the presence of AREs does not mean that HuR binds to them, we confirmed the binding using PAR-CLIP data (Kishore et al., 2011; Lebedeva et al., 2011; Mukherjee et al., 2011). Among the 4,279 transcripts bound by ADAR1, 2,866 (67%) were also bound by HuR in PAR-CLIP, which is significantly more than observed in random transcripts (36%; χ^2, p < 0.0001), showing that HuR binds to ADAR1 targets in vivo. These common binding targets of HuR and ADAR1 include *MCM4*, which plays a key role in DNA replication; *TMPO*, which encodes a nuclear membrane protein; and *GSR*, which encodes the enzyme glutathione reductase in the antioxidative stress pathway.

The enrichment of HuR-binding sites in ADAR targets and the identification of an RNA-dependent HuR-ADAR complex led us to reason that HuR and ADAR bind to common transcripts and regulate them cooperatively. We confirmed our hypothesis by employing two experimental approaches. First, we carried out RNA pull-down assays. We prepared in vitro synthesized and biotinylated RNA for three transcripts (*MCM4*, *CTH*, and *GSR*) that we previously identified as shared targets of ADAR and HuR. After incubating these transcripts with B cell lysates, we pulled down the transcripts using their biotin tags and immunoblotted for ADAR and HuR proteins. Our results showed that ADAR and HuR are specifically pulled down on *MCM4* and *GSR* transcripts, confirming concurrent ADAR and HuR binding (Figure 6C). Although it binds strongly to HuR, the *CTH* transcript pulled down less ADAR1, suggesting its weaker interaction with ADAR1 compared with *MCM4* and *GSR*. Second, we carried out HuR RNA-IP to confirm that HuR binds to the same transcripts that ADAR1 targets, and then examined the effects of such binding. Using HuR antibody, we pulled down HuR protein and tested whether ADAR1-targeted transcripts were pulled down with HuR in human B cells. The results showed that HuR antibody, but not negative-control IgG, pulled down the same transcripts that immunoprecipitated with ADAR1 antibody, including *MCM4*, *TMPO*, *GSR*, and *CTH* (Figure 6D).

We then asked whether HuR and ADAR1 depend on each other for binding to their common targets. Previous studies have found that HuR and other RNA-binding proteins cooperate by binding to the same RNA substrates (Chang et al., 2010; Lal et al., 2004). We carried out HuR RNA-IP following siRNA knockdown of *ADAR1* and found that following *ADAR1* knockdown, binding of HuR to its target transcripts is greatly reduced (Figure 6E). In cells transfected with *ADAR1* siRNAs, the protein level in HuR is the same as that in controls (Figure S2B), confirming that the decrease in HuR binding is not due to decreased HuR protein expression.

After identifying that ADAR is required for HuR binding to transcripts, we examined the effects of ADAR and HuR on

transcript levels. Since HuR regulates gene expression by stabilizing mRNAs (Myer et al., 1997), we examined whether ADAR binding affects transcript stability through HuR. Among the 775 genes whose expression levels decreased following *ADAR1* knockdown, there were significantly more genes containing HuR-binding sites than genes whose expression levels increased following *ADAR1* knockdown (χ^2, $p < 0.01$). For example, the expression levels of *MCM4*, *TMPO*, *GSR*, and *CTH* transcripts were reduced in both individuals following *ADAR1* knockdown, consistent with binding of their transcripts by both HuR and ADAR1 (Figure 6F). These results support the notion that in the absence of ADAR1, HuR binding decreased; thus, the target genes were not stabilized, resulting in lower gene expression. Lastly, these data suggest that *ADAR1* and *HuR* expression levels should correlate with the expression levels of their target genes. Using results from another study in our lab (Cheung et al., 2010), we compared the expression levels of the target genes with *ADAR1* and *HuR* in cultured B cells from 41 unrelated individuals and found that they were significantly correlated ($p << 0.01$). Correlation plots for *MCM4* and *TMPO* with *ADAR1* and *HuR* are shown in Figure S4A.

Our findings suggest that ADAR1 and HuR proteins cooperate to regulate RNA processing through editing and mRNA turnover. These proteins coregulate transcripts by binding to specific sequences and secondary structures that mediate these processing steps.

DISCUSSION

In this study, we uncovered ~60,000 A-to-G RNA editing sites mediated by ADAR1 and ADAR2 proteins in human B cells. We show that ADAR proteins are involved in gene regulation, particularly in regulating RNA stability and processing.

Prior to our study, many A-to-G editing sites had been identified. Here, we added to the list of such sites by using gene knockdown and RNA-IP, and we validated experimentally that our sites are direct targets of ADAR1 and ADAR2 proteins. Traditionally, editing sites are identified by comparing DNA and RNA sequences. Often the DNA sequences used for comparisons are those from the reference genome. We extracted the DNA and RNA from the same cells and subjected them to deep sequencing, which allowed a direct comparison of RNA sequences and their corresponding DNA. Although NGS provides sequence information with unprecedented coverage, there are hundreds of millions of sequence reads that have to be mapped correctly for proper interpretation. To have confidence in our sequence mapping, we set stringent analysis thresholds that required uniquely mapped reads from two different sequence alignment algorithms (GSNAP and blat) and at least ten sequence reads at each site. However, computational analysis alone may not be adequate. To determine a list of high-confidence ADAR targets, we coupled deep sequencing with *ADAR* gene knockdowns and ADAR RNA-IP. The same analysis method was used to analyze sequence reads from all samples, and thus the sites in which editing is responsive to gene knockdown, or that are bound specifically to ADAR proteins, cannot be artifacts of computational analyses. In a recent study on RNA editing in *Drosophila* (Rodriguez et al., 2012), RNA-seq of nascent RNA from an *ADAR* null strain was compared with that of a wild-type strain. The results were used to estimate an FDR of ~5%. In our study, we used a similar approach and estimated our FDR to be ~4%.

The large number of sites in which RNA sequences differed from the underlying DNA sequences is surprising and requires further attention in genetic studies. Results from this and other studies show that there are likely many thousands of A-to-G editing sites in each individual. Previously, we showed that there are individual differences in the number of RDDs (Li et al., 2011). Here, in our two subjects, we also observed differences in the number of editing sites and the level of editing. These results indicate that genetic variation can extend beyond DNA sequence variation. Even though two individuals may have the same DNA sequences at a site, their RNA sequences may differ. To date, most genetic studies have focused on DNA sequence variation in looking for disease-susceptibility alleles. As it becomes clear that RNA sequence variation extends beyond DNA sequence polymorphism, RNA editing and other types of RDDs will have to be considered in studies to identify the genetic basis of human diseases and traits. Comprehensive lists of editing and RDD sites, such as those presented in this study, are important for facilitating the inclusion of RNA variants in genetic studies.

RNA transcripts are tethered to regulatory factors, and the combinatorial binding of RBPs to transcripts coordinates different steps of RNA processing (Hogan et al., 2008; Licatalosi and Darnell, 2010; Maniatis and Reed, 2002). We found enrichment of binding sequences for HuR in transcripts edited by ADAR. Computational and experimental evidence from HuR RNA-IP in human B cells and cells transfected with ADAR siRNAs showed that HuR binding is facilitated by ADAR binding to RNAs. Our results are consistent with a model in which binding of ADAR to RNA forms secondary structures that are then recognized by HuR proteins. Thus, RNA sequences and structures allow gene regulation by a combination of different RNA processing proteins. Transcription factors cooperate to mediate gene regulation; similarly, RNA processing proteins coordinate to affect gene expression. The complex regulatory codes involve RNA sequences and structures that are facilitated by different combinations of RNA-binding proteins. Therefore, to understand co- and posttranscriptional regulation of gene expression, we need to go beyond studying single proteins. Experimental methods that examine protein complexes and their target RNAs are needed to enhance our understanding of gene regulation.

In summary, in this work we studied ADAR-mediated RNA editing and gene-expression regulation. Our findings uncover editing targets, reveal ADARs' role in mediating RNA editing and regulation of gene expression, and show that the ADAR protein complex coordinates multiple steps in RNA processing. However, they also raise new questions. Our findings suggest that other mechanisms, such as those that mediate non-A-to-G type RDDs, remain to be identified. In addition, the RNA sequence and structural signatures of the regulatory codes for co- and posttranscriptional processing are largely unknown. Elucidating ADAR's functions will further our

understanding of RNA processing and provide insights into human diseases.

EXPERIMENTAL PROCEDURES

Identification of Editing and RDDs

B cell lines from two individuals in the Centre d'Étude du Polymorphisme Humain database were cultured and genomic DNA and RNA were extracted. DNA-seq and RNA-seq libraries were prepared and sequenced on a HiSeq 2000 instrument (Illumina). DNA-seq and RNA-seq data were aligned to the reference genome (HG18) using CASAVA and GSNAP, respectively. To identify RDDs, we compared each RNA sequence with its corresponding DNA sequence. We required an editing site or RDD site to be covered by a minimum of 10 total DNA-seq and RNA-seq reads, 100% concordance in the DNA sequence, an RDD level $\geq$ 10%, and an RDD event to be found in both individuals. Potential sites were then filtered using stringent thresholds.

Validation of RDDs using Sanger Sequencing and Droplet Digital PCR

Cultured B cells were transfected with Accell siRNAs (Thermo Scientific) against *ADAR1* and *ADAR2*. Sequences surrounding RDD sites were PCR amplified using genomic DNA or cDNA as the template, and PCR products were sequenced. The 3′ UTR of ATM was amplified from cDNA of B cells and cloned into TOPO vector (Invitrogen).

For droplet digital PCR, DNA probes specific to the DNA and RNA variants at RDD sites were synthesized and labeled by VIC and FAM, respectively (ABI Biosystems). Emulsion PCR was carried out and quantified on a QuantaLIfe Droplet Reader (Bio-Rad Laboratories).

RNA-IP

Anti-ADAR1 and anti-HuR RNA-IP was carried out with a Magna RNA-Binding Protein Immunoprecipitation Kit (Millipore). Quantitative PCR and RNA-seq of immunoprecipitated transcripts were carried out. RNA-editing sites that were detected in transcripts pulled down by ADAR1 antibody, but not by negative-control IgG, were identified as ADAR1-specific targets.

RNA-Protein Pull-Down Assays

Transcripts of HuR and ADAR1 targets were synthesized and biotin labeled in vitro, and incubated with whole-cell lysates. RNA-protein complexes were pulled down and analyzed by western blot (Pierce).

Protein IP of the ADAR-HuR Complex

B cell lysates were incubated with anti-ADAR1 or negative-control rabbit IgG at 4°C overnight. The immunocomplex was pulled down using Protein A agarose (Roche), washed, and finally eluted in 20 mM Tris/7.5, 150 mM NaCl, 2.5 mM $MgCl_2$, 0.2% SDS. To examine RNA-dependent interactions, whole-cell lysates were diluted to 1 μg/μl, RNase A or RNase V1 was added, and lysates were incubated at room temperature for 15 min. Protein samples were analyzed by western blot.

A detailed description of the materials and methods used in this work is provided in the Supplemental Experimental Procedures.

ACCESSION NUMBERS

Sequencing data have been deposited in the NCBI Gene Expression Omnibus under accession numbers ERP001478 (DNA-seq) and GSE38233 (RNA-seq).

SUPPLEMENTAL INFORMATION

Supplemental Information includes Supplemental Experimental Procedures, four figures, and ten tables and can be found with this article online at http://dx.doi.org/10.1016/j.celrep.2013.10.002.

ACKNOWLEDGMENTS

We thank Dr. Alan Bruzel, Susannah Elwyn, Zhengwei Zhu, Allison Richards, and Jonathan Toung for discussions and technical support and Colleen McGarry for manuscript preparation. This work was supported by grants from the National Institutes of Health (to V.G.C.) and by the Howard Hughes Medical Institute.

Received: March 26, 2013
Revised: July 26, 2013
Accepted: October 1, 2013
Published: October 31, 2013

REFERENCES

Ashburner, M., Ball, C.A., Blake, J.A., Botstein, D., Butler, H., Cherry, J.M., Davis, A.P., Dolinski, K., Dwight, S.S., Eppig, J.T., et al.; The Gene Ontology Consortium. (2000). Gene ontology: tool for the unification of biology. Nat. Genet. *25*, 25–29.

Athanasiadis, A., Rich, A., and Maas, S. (2004). Widespread A-to-I RNA editing of Alu-containing mRNAs in the human transcriptome. PLoS Biol. *2*, e391.

Bahn, J.H., Lee, J.-H., Li, G., Greer, C., Peng, G., and Xiao, X. (2012). Accurate identification of A-to-I RNA editing in human by transcriptome sequencing. Genome Res. *22*, 142–150.

Bailey, T.L., Boden, M., Buske, F.A., Frith, M., Grant, C.E., Clementi, L., Ren, J., Li, W.W., and Noble, W.S. (2009). MEME SUITE: tools for motif discovery and searching. Nucleic Acids Res. *37*(Web Server issue), W202–W208.

Bass, B.L., and Weintraub, H. (1988). An unwinding activity that covalently modifies its double-stranded RNA substrate. Cell *55*, 1089–1098.

Bentley, D.R., Balasubramanian, S., Swerdlow, H.P., Smith, G.P., Milton, J., Brown, C.G., Hall, K.P., Evers, D.J., Barnes, C.L., Bignell, H.R., et al. (2008). Accurate whole human genome sequencing using reversible terminator chemistry. Nature *456*, 53–59.

Blow, M., Futreal, P.A., Wooster, R., and Stratton, M.R. (2004). A survey of RNA editing in human brain. Genome Res. *14*, 2379–2387.

Carmi, S., Borukhov, I., and Levanon, E.Y. (2011). Identification of widespread ultra-edited human RNAs. PLoS Genet. *7*, e1002317.

Chang, N., Yi, J., Guo, G., Liu, X., Shang, Y., Tong, T., Cui, Q., Zhan, M., Gorospe, M., and Wang, W. (2010). HuR uses AUF1 as a cofactor to promote p16INK4 mRNA decay. Mol. Cell. Biol. *30*, 3875–3886.

Chen, S.H., Habib, G., Yang, C.Y., Gu, Z.W., Lee, B.R., Weng, S.A., Silberman, S.R., Cai, S.J., Deslypere, J.P., Rosseneu, M., et al. (1987). Apolipoprotein B-48 is the product of a messenger RNA with an organ-specific in-frame stop codon. Science *238*, 363–366.

Chen, C.X., Cho, D.S., Wang, Q., Lai, F., Carter, K.C., and Nishikura, K. (2000). A third member of the RNA-specific adenosine deaminase gene family, ADAR3, contains both single- and double-stranded RNA binding domains. RNA *6*, 755–767.

Chen, R., Mias, G.I., Li-Pook-Than, J., Jiang, L., Lam, H.Y.K., Chen, R., Miriami, E., Karczewski, K.J., Hariharan, M., Dewey, F.E., et al. (2012). Personal omics profiling reveals dynamic molecular and medical phenotypes. Cell *148*, 1293–1307.

Cheung, V.G., Nayak, R.R., Wang, I.X., Elwyn, S., Cousins, S.M., Morley, M., and Spielman, R.S. (2010). Polymorphic cis- and trans-regulation of human gene expression. PLoS Biol. *8*, e1000480.

Chilibeck, K.A., Wu, T., Liang, C., Schellenberg, M.J., Gesner, E.M., Lynch, J.M., and MacMillan, A.M. (2006). FRET analysis of in vivo dimerization by RNA-editing enzymes. J. Biol. Chem. *281*, 16530–16535.

Clerzius, G., Gélinas, J.-F., Daher, A., Bonnet, M., Meurs, E.F., and Gatignol, A. (2009). ADAR1 interacts with PKR during human immunodeficiency virus infection of lymphocytes and contributes to viral replication. J. Virol. *83*, 10119–10128.

Daniel, C., Venø, M.T., Ekdahl, Y., Kjems, J., and Öhman, M. (2012). A distant cis acting intronic element induces site-selective RNA editing. Nucleic Acids Res. *40*, 9876–9886.

Dawson, T.R., Sansam, C.L., and Emeson, R.B. (2004). Structure and sequence determinants required for the RNA editing of ADAR2 substrates. J. Biol. Chem. *279*, 4941–4951.

Fan, X.C., and Steitz, J.A. (1998). Overexpression of HuR, a nuclear-cytoplasmic shuttling protein, increases the in vivo stability of ARE-containing mRNAs. EMBO J. *17*, 3448–3460.

Grimson, A., Farh, K.K.-H., Johnston, W.K., Garrett-Engele, P., Lim, L.P., and Bartel, D.P. (2007). MicroRNA targeting specificity in mammals: determinants beyond seed pairing. Mol. Cell *27*, 91–105.

Heale, B.S.E., Keegan, L.P., McGurk, L., Michlewski, G., Brindle, J., Stanton, C.M., Caceres, J.F., and O'Connell, M.A. (2009). Editing independent effects of ADARs on the miRNA/siRNA pathways. EMBO J. *28*, 3145–3156.

Hogan, D.J., Riordan, D.P., Gerber, A.P., Herschlag, D., and Brown, P.O. (2008). Diverse RNA-binding proteins interact with functionally related sets of RNAs, suggesting an extensive regulatory system. PLoS Biol. *6*, e255.

Huang, W., Sherman, B.T., and Lempicki, R.A. (2009a). Systematic and integrative analysis of large gene lists using DAVID bioinformatics resources. Nat. Protoc. *4*, 44–57.

Huang, W., Sherman, B.T., and Lempicki, R.A. (2009b). Bioinformatics enrichment tools: paths toward the comprehensive functional analysis of large gene lists. Nucleic Acids Res. *37*, 1–13.

Ju, Y.S., Kim, J.-I., Kim, S., Hong, D., Park, H., Shin, J.-Y., Lee, S., Lee, W.-C., Kim, S., Yu, S.-B., et al. (2011). Extensive genomic and transcriptional diversity identified through massively parallel DNA and RNA sequencing of eighteen Korean individuals. Nat. Genet. *43*, 745–752.

Kim, U., Wang, Y., Sanford, T., Zeng, Y., and Nishikura, K. (1994). Molecular cloning of cDNA for double-stranded RNA adenosine deaminase, a candidate enzyme for nuclear RNA editing. Proc. Natl. Acad. Sci. USA *91*, 11457–11461.

Kim, D.D.Y., Kim, T.T.Y., Walsh, T., Kobayashi, Y., Matise, T.C., Buyske, S., and Gabriel, A. (2004). Widespread RNA editing of embedded alu elements in the human transcriptome. Genome Res. *14*, 1719–1725.

Kiran, A., and Baranov, P.V. (2010). DARNED: a DAtabase of RNa EDiting in humans. Bioinformatics *26*, 1772–1776.

Kishore, S., Jaskiewicz, L., Burger, L., Hausser, J., Khorshid, M., and Zavolan, M. (2011). A quantitative analysis of CLIP methods for identifying binding sites of RNA-binding proteins. Nat. Methods *8*, 559–564.

Klaue, Y., Källman, A.M., Bonin, M., Nellen, W., and Ohman, M. (2003). Biochemical analysis and scanning force microscopy reveal productive and nonproductive ADAR2 binding to RNA substrates. RNA *9*, 839–846.

Kuttan, A., and Bass, B.L. (2012). Mechanistic insights into editing-site specificity of ADARs. Proc. Natl. Acad. Sci. USA *109*, E3295–E3304.

Lal, A., Mazan-Mamczarz, K., Kawai, T., Yang, X., Martindale, J.L., and Gorospe, M. (2004). Concurrent versus individual binding of HuR and AUF1 to common labile target mRNAs. EMBO J. *23*, 3092–3102.

Lebedeva, S., Jens, M., Theil, K., Schwanhäusser, B., Selbach, M., Landthaler, M., and Rajewsky, N. (2011). Transcriptome-wide analysis of regulatory interactions of the RNA-binding protein HuR. Mol. Cell *43*, 340–352.

Lehmann, K.A., and Bass, B.L. (2000). Double-stranded RNA adenosine deaminases ADAR1 and ADAR2 have overlapping specificities. Biochemistry *39*, 12875–12884.

Levanon, E.Y., Eisenberg, E., Yelin, R., Nemzer, S., Hallegger, M., Shemesh, R., Fligelman, Z.Y., Shoshan, A., Pollock, S.R., Sztybel, D., et al. (2004). Systematic identification of abundant A-to-I editing sites in the human transcriptome. Nat. Biotechnol. *22*, 1001–1005.

Li, J.B., Levanon, E.Y., Yoon, J.-K., Aach, J., Xie, B., Leproust, E., Zhang, K., Gao, Y., and Church, G.M. (2009). Genome-wide identification of human RNA editing sites by parallel DNA capturing and sequencing. Science *324*, 1210–1213.

Li, M., Wang, I.X., Li, Y., Bruzel, A., Richards, A.L., Toung, J.M., and Cheung, V.G. (2011). Widespread RNA and DNA sequence differences in the human transcriptome. Science *333*, 53–58.

Licatalosi, D.D., and Darnell, R.B. (2010). RNA processing and its regulation: global insights into biological networks. Nat. Rev. Genet. *11*, 75–87.

Maniatis, T., and Reed, R. (2002). An extensive network of coupling among gene expression machines. Nature *416*, 499–506.

Mukherjee, N., Corcoran, D.L., Nusbaum, J.D., Reid, D.W., Georgiev, S., Hafner, M., Ascano, M., Jr., Tuschl, T., Ohler, U., and Keene, J.D. (2011). Integrative regulatory mapping indicates that the RNA-binding protein HuR couples pre-mRNA processing and mRNA stability. Mol. Cell *43*, 327–339.

Myer, V.E., Fan, X.C., and Steitz, J.A. (1997). Identification of HuR as a protein implicated in AUUUA-mediated mRNA decay. EMBO J. *16*, 2130–2139.

Nie, Y., Ding, L., Kao, P.N., Braun, R., and Yang, J.-H. (2005). ADAR1 interacts with NF90 through double-stranded RNA and regulates NF90-mediated gene expression independently of RNA editing. Mol. Cell. Biol. *25*, 6956–6963.

Osenberg, S., Dominissini, D., Rechavi, G., and Eisenberg, E. (2009). Widespread cleavage of A-to-I hyperediting substrates. RNA *15*, 1632–1639.

Peng, Z., Cheng, Y., Tan, B.C.-M., Kang, L., Tian, Z., Zhu, Y., Zhang, W., Liang, Y., Hu, X., Tan, X., et al. (2012). Comprehensive analysis of RNA-seq data reveals extensive RNA editing in a human transcriptome. Nat. Biotechnol. *30*, 253–260.

Powell, L.M., Wallis, S.C., Pease, R.J., Edwards, Y.H., Knott, T.J., and Scott, J. (1987). A novel form of tissue-specific RNA processing produces apolipoprotein-B48 in intestine. Cell *50*, 831–840.

Rodriguez, J., Menet, J.S., and Rosbash, M. (2012). Nascent-seq indicates widespread cotranscriptional RNA editing in Drosophila. Mol. Cell *47*, 27–37.

Rueter, S.M., Burns, C.M., Coode, S.A., Mookherjee, P., and Emeson, R.B. (1995). Glutamate receptor RNA editing in vitro by enzymatic conversion of adenosine to inosine. Science *267*, 1491–1494.

Scadden, A.D., and Smith, C.W. (2001). Specific cleavage of hyper-edited dsRNAs. EMBO J. *20*, 4243–4252.

Shen, S., Lin, L., Cai, J.J., Jiang, P., Kenkel, E.J., Stroik, M.R., Sato, S., Davidson, B.L., and Xing, Y. (2011). Widespread establishment and regulatory impact of Alu exons in human genes. Proc. Natl. Acad. Sci. USA *108*, 2837–2842.

Trapnell, C., Hendrickson, D.G., Sauvageau, M., Goff, L., Rinn, J.L., and Pachter, L. (2013). Differential analysis of gene regulation at transcript resolution with RNA-seq. Nat. Biotechnol. *31*, 46–53.

Wong, S.K., Sato, S., and Lazinski, D.W. (2001). Substrate recognition by ADAR1 and ADAR2. RNA *7*, 846–858.

Wu, D., Lamm, A.T., and Fire, A.Z. (2011). Competition between ADAR and RNAi pathways for an extensive class of RNA targets. Nat. Struct. Mol. Biol. *18*, 1094–1101.

Yang, J.H., Sklar, P., Axel, R., and Maniatis, T. (1995). Editing of glutamate receptor subunit B pre-mRNA in vitro by site-specific deamination of adenosine. Nature *374*, 77–81.

Yang, W., Wang, Q., Howell, K.L., Lee, J.T., Cho, D.-S.C., Murray, J.M., and Nishikura, K. (2005). ADAR1 RNA deaminase limits short interfering RNA efficacy in mammalian cells. J. Biol. Chem. *280*, 3946–3953.

ADAR Regulates RNA Editing, Transcript Stability and Gene Expression

Isabel X. Wang, Elizabeth So, James L. Devlin, Yue Zhao, Ming Wu, Vivian G. Cheung

Supplemental Figure Legends

Figure S1. Validation of A-to-G editing, related to Figure 2. (A) An example of validation of A-to-G editing by droplet digital PCR. Shown here is the editing site at chr5:49728037 in *EMB*. Genomic DNA or cDNA from GM12750 was used as input for PCR amplified by gene-specific primers and fluorescence-labeled allele-specific probes. (B) Summary of sites that are validated experimentally.

Figure S2. Examined off-target effects of siRNA by testing 4 independent specific siRNAs and pooled siRNAs, related to Figure 3, 4 and 6. (A) mRNA expression levels of *ADAR1* and *ADAR2* are reduced by 4 individual siRNAs and pooled siRNA, shown by real-time RT-PCR. (B) Western blot confirms reduced ADAR1 at protein level. In contrast, HuR expression is not affect by *ADAR1* knockdown. (C) Sanger sequencing confirms that knockdown of *ADAR1* by each siRNA led to reduced editing levels at chr2:37184007. Sequence traces represent the reverse strand of cDNA. Error bar: SEM of triplicate experiments.

Figure S3. Dynamic changes in editing levels and gene expression following *ADAR1* knockdown, related to Figure 3. (A) A 96-hour time course of ADAR1-knockdown combined with RNAseq uncovered dynamic changes in editing levels. As a result of ADAR1-siRNA treatment, mRNA levels of ADAR1 remained low (<50% of control sample) for up to 96 hours post-transfection. (B) Examples of A-to-G sites where the editing levels remained low throughout the time-course. (C) Example of genes that did not change significantly at total gene level but showed “isoform switching” following *ADAR1* knockdown.

Figure S4. Correlation of gene expression between ADAR1 and HuR, and overlap between our study and previous published studies, related to Figure 3 and 6. (A) Gene expression level of HuR and ADAR1 among 41 unrelated individuals are significantly correlated with that of their target genes (P<10-7). Gene expression levels (RPKM) of HuR, ADAR1, MCM4 and TMPO were calculated from RNA-seq data of 41 unrelated individuals. (B)&(C) Editing sites identified in current study (GM12004 and GM12750) are compared to those from (B) all previously published data or (C) Peng et al, 2012. Comparison is based on overlapping (by site) or overlapping genes (by gene).

A

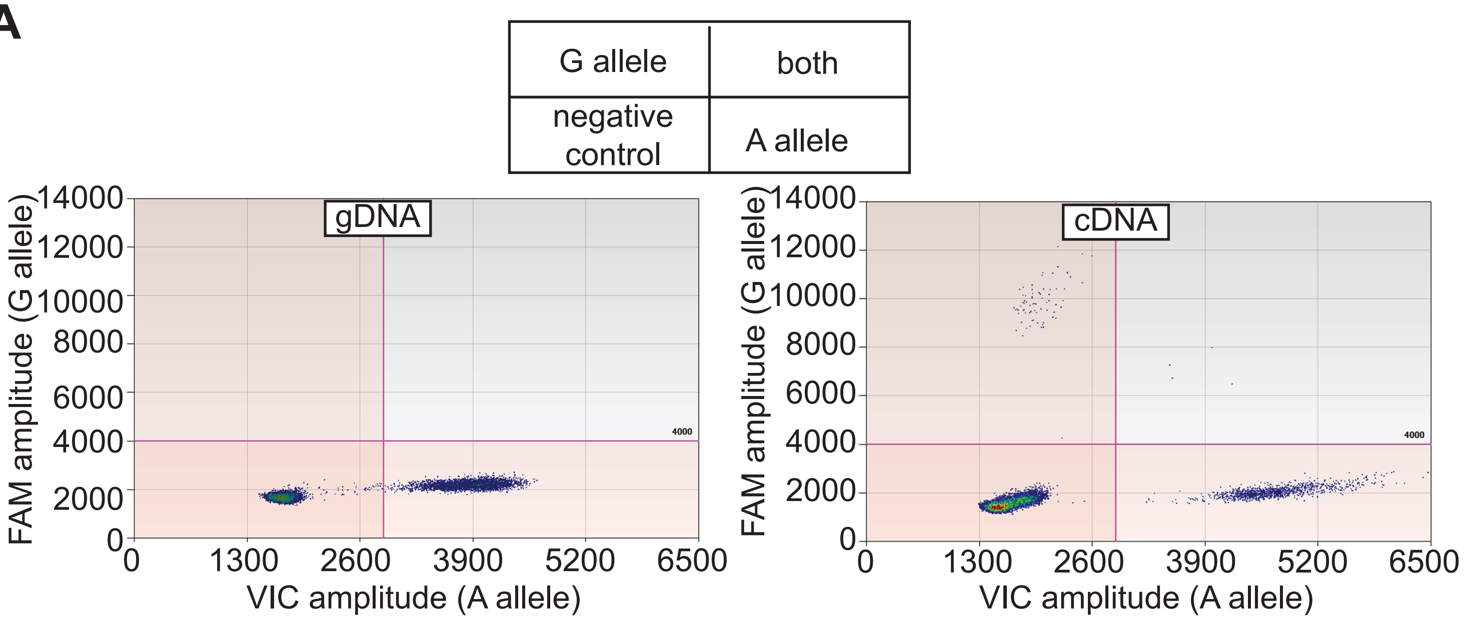

B

Chr	Position	Gene Symbol	Type	Location	Strand	In Alu	Average Level	Validation method	Validated?
11	107741679	*ATM*	A>G	3UTR	+	Yes	0.62	Cloning/Sanger	Yes
11	107741687	*ATM*	A>G	3UTR	+	Yes	0.49	Cloning/Sanger	Yes
11	107741695	*ATM*	A>G	3UTR	+	Yes	0.46	Cloning/Sanger	Yes
11	107741696	*ATM*	A>G	3UTR	+	Yes	0.44	Cloning/Sanger	Yes
11	107741723	*ATM*	A>G	3UTR	+	Yes	0.07	Cloning/Sanger	Yes
11	107741733	*ATM*	A>G	3UTR	+	Yes	0.38	Cloning/Sanger	Yes
11	107741734	*ATM*	A>G	3UTR	+	Yes	0.12	Cloning/Sanger	Yes
11	107741810	*ATM*	A>G	3UTR	+	Yes	0.08	Cloning/Sanger	Yes
11	107741819	*ATM*	A>G	3UTR	+	Yes	0.21	Cloning/Sanger	Yes
11	107741827	*ATM*	A>G	3UTR	+	Yes	0.24	Cloning/Sanger	Yes
11	107741845	*ATM*	A>G	3UTR	+	Yes	0.18	Cloning/Sanger	Yes
11	107741851	*ATM*	A>G	3UTR	+	Yes	0.24	Cloning/Sanger	Yes
6	90487981	*MDN1*	C>A	intron	+	No	1	PCR/Sanger	Yes
7	89857293	*GTPBP10*	A>G	3UTR	+	Yes	0.63	PCR/Sanger	Yes
7	89857303	*GTPBP10*	A>G	3UTR	+	Yes	0.09	PCR/Sanger	Yes
9	32446365	*DDX58*	A>G	3UTR	-	No	0.07	PCR/Sanger	Yes
9	32446368	*DDX58*	A>G	3UTR	-	No	0.12	PCR/Sanger	Yes
9	32446371	*DDX58*	A>G	3UTR	-	No	0.1	PCR/Sanger	No
10	74678737	*MRPS16*	A>G	3UTR,stop_codon	-	Yes	0.35	PCR/Sanger	Yes
10	114199337	*VTI1A*	A>G	mixed, exon	+	Yes	0.38	PCR/Sanger	Yes
10	114199343	*VTI1A*	A>G	mixed, exon	+	Yes	0.27	PCR/Sanger	Yes
14	19905006	*TEP1*	A>G	3UTR	-	No	0.11	PCR/Sanger	Yes
14	52179482	*ERO1L*	A>G	3UTR	-	No	0.05	PCR/Sanger	Yes
14	52179560	*ERO1L*	A>G	3UTR	-	No	0.15	PCR/Sanger	Yes
1	159227224	*F11R*	A>G	intergenic	-	No	0.2	droplet digital PCR	Yes
2	20314300	*PUM2*	A>G	3UTR	-	No	0.21	droplet digital PCR	Yes
3	151819253	*SELT*	A>G	mixed, exon	+	Yes	0.22	droplet digital PCR	No
5	49728037	*EMB*	A>G	3UTR	-	No	0.08	droplet digital PCR	Yes
5	67632909	*PIK3R1*	A>G	3UTR	+	No	0.1	droplet digital PCR	Yes
21	33845189	*SON*	A>G	coding exon	+	No	0.15	droplet digital PCR	Yes

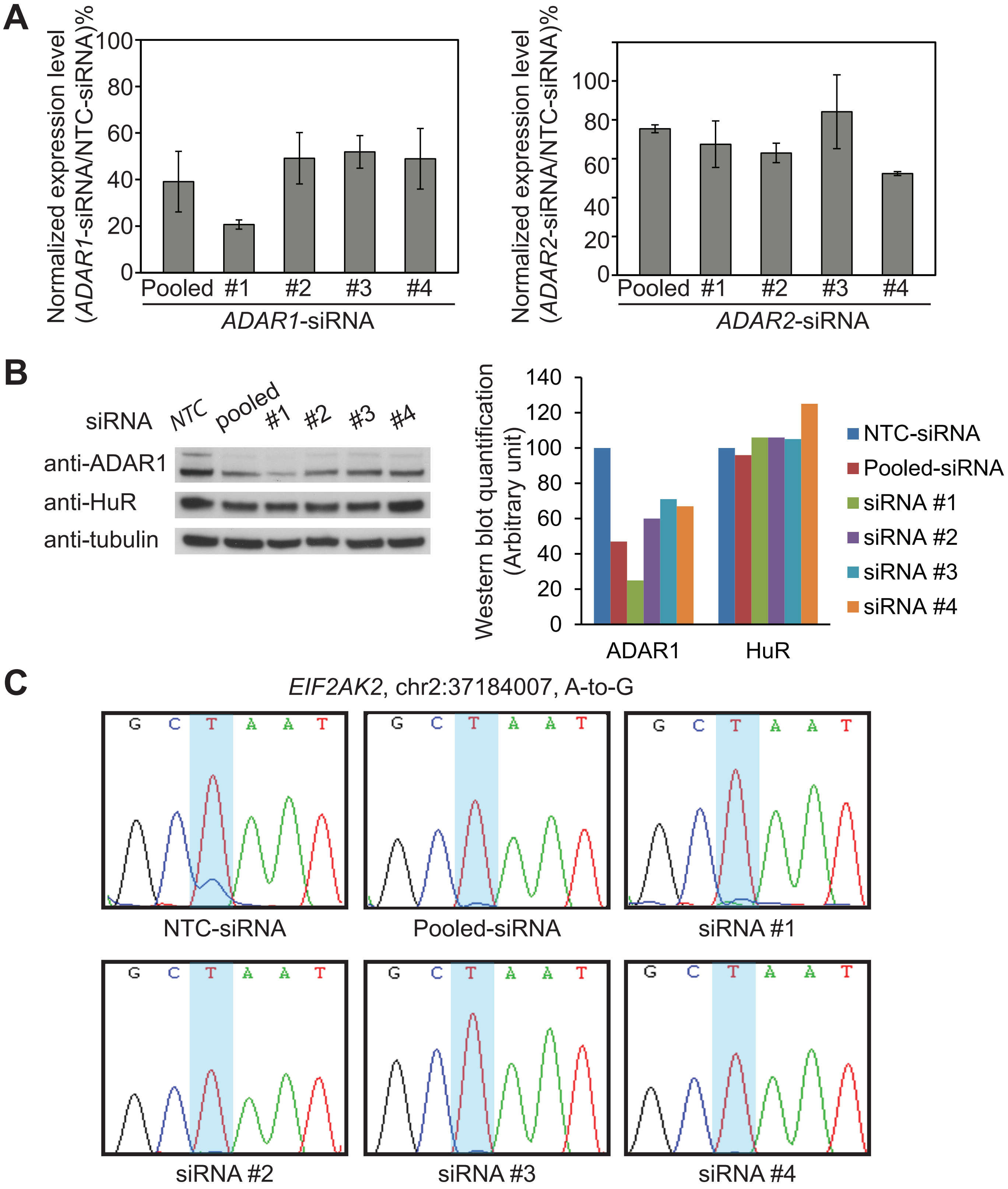

A
Normalized expression level
(ADAR1-siRNA/NTC-siRNA)%
100
80
60
40
20
0
Pooled
#1
#2
#3
#4
ADAR1-siRNA
Normalized expression level
(ADAR2-siRNA/NTC-siRNA)%
100
80
60
40
20
0
Pooled
#1
#2
#3
#4
ADAR2-siRNA
B
siRNA
NTC
pooled
#1
#2
#3
#4
anti-ADAR1
anti-HuR
anti-tubulin
Western blot quantification
(Arbitrary unit)
140
120
100
80
60
40
20
0
ADAR1
HuR
NTC-siRNA
Pooled-siRNA
siRNA #1
siRNA #2
siRNA #3
siRNA #4
C
EIF2AK2, chr2:37184007, A-to-G
G C T A A T
NTC-siRNA
Pooled-siRNA
siRNA #1
siRNA #2
siRNA #3
siRNA #4

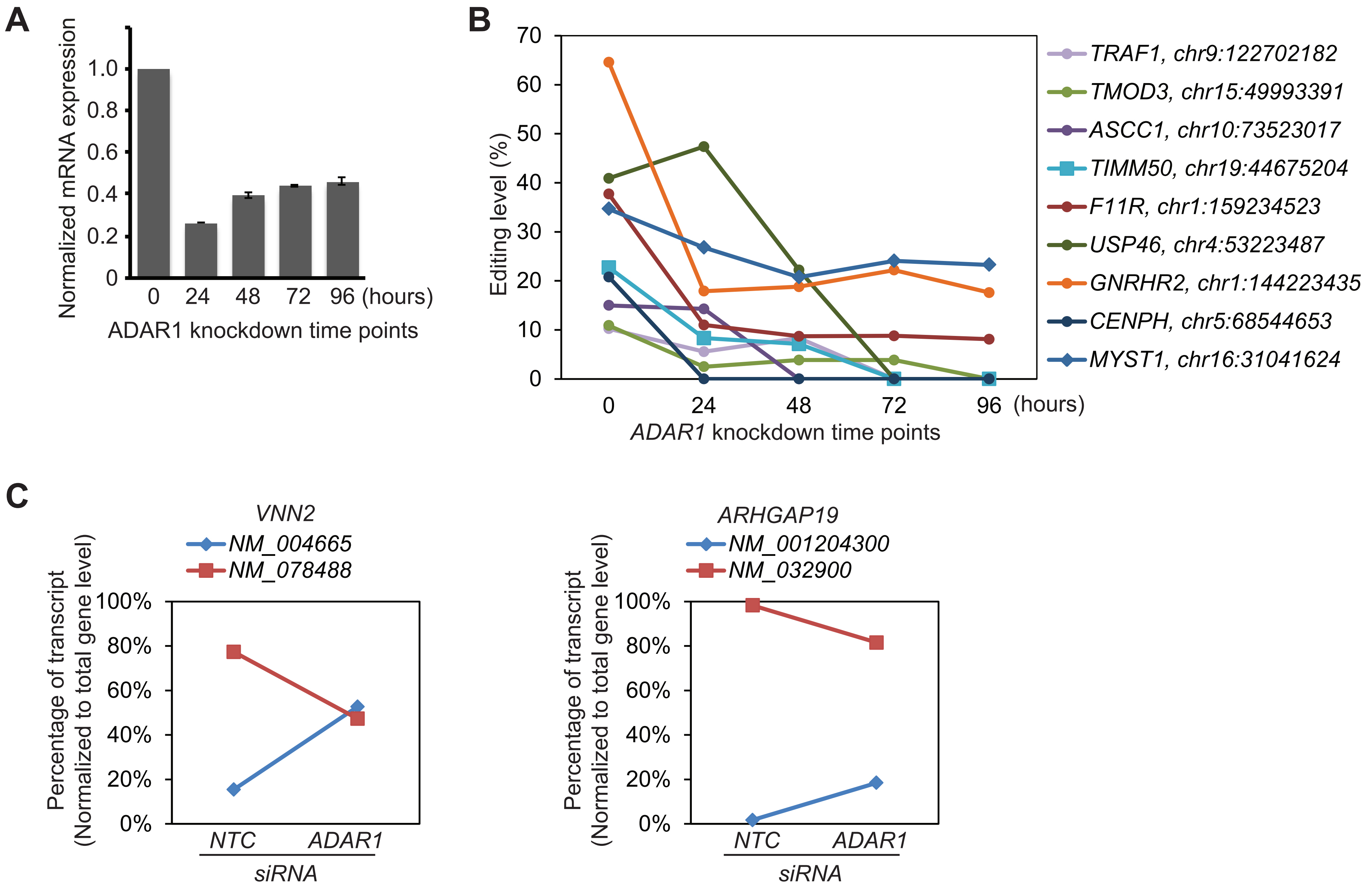
A
Normalized mRNA expression
1.0
0.8
0.6
0.4
0.2
0
0 24 48 72 96 (hours)
ADAR1 knockdown time points
B
Editing level (%)
70
60
50
40
30
20
10
0
0 24 48 72 96 (hours)
ADAR1 knockdown time points
TRAF1, chr9:122702182
TMOD3, chr15:49993391
ASCC1, chr10:73523017
TIMM50, chr19:44675204
F11R, chr1:159234523
USP46, chr4:53223487
GNRHR2, chr1:144223435
CENPH, chr5:68544653
MYST1, chr16:31041624
C
VNN2
NM_004665
NM_078488
Percentage of transcript (Normalized to total gene level)
100%
80%
60%
40%
20%
0%
NTC ADAR1
siRNA
ARHGAP19
NM_001204300
NM_032900
Percentage of transcript (Normalized to total gene level)
100%
80%
60%
40%
20%
0%
NTC ADAR1
siRNA

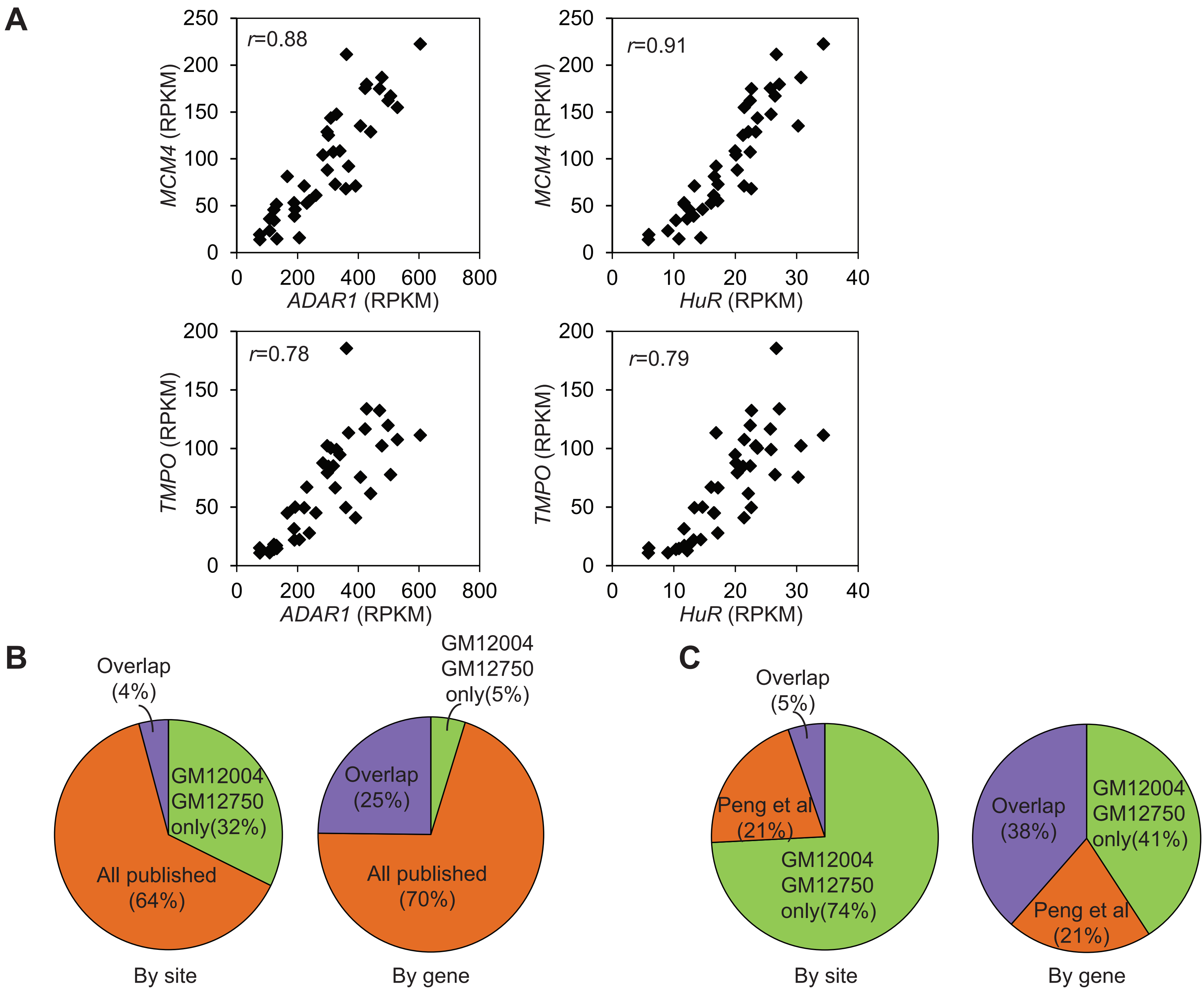
A
r=0.88
MCM4 (RPKM)
ADAR1 (RPKM)
r=0.91
MCM4 (RPKM)
HuR (RPKM)
r=0.78
TMPO (RPKM)
ADAR1 (RPKM)
r=0.79
TMPO (RPKM)
HuR (RPKM)
B
Overlap (4%)
GM12004 GM12750 only(32%)
All published (64%)
By site
GM12004 GM12750 only(5%)
Overlap (25%)
All published (70%)
By gene
C
Overlap (5%)
Peng et al (21%)
GM12004 GM12750 only(74%)
By site
Overlap (38%)
GM12004 GM12750 only(41%)
Peng et al (21%)
By gene

Supplemental Table Titles

Table S1 Summary of RNA-seq data, related to Figure 1.

Table S2 RNA-DNA differences identified from analysis of RNA-seq and DNA-seq of B-cells from two individuals, related to Figure 1.

Table S3 ADAR1 knockdown leads to changes in editing levels of target genes, related to Figure 3.

Table S4 ADAR2 knockdown leads to changes in editing levels of target genes, related to Figure 4.

Table S5 Editing sites identified from anti-ADAR1 RNA-immunoprecipitation experiment, related to Figure 4.

Table S6 Genes whose expression levels changed significantly following *ADAR1* knockdown in both individuals, related to Figure 3.

Table S7 Gene ontology analysis of genes whose expression levels changed following ADAR1 knockdown, related to Figure 3.

Table S8 Genes whose expression levels and editing levels both changed at least 20% following ADAR1 knockdown, related to Figure 5.

Table S9 Genes whose expression levels changed at least 2 fold following ADAR2 knockdown, related to Figure 4.

Table S10 Primers used in this study, related to experimental procedures.

Table S1. Summary of RNA-seq data, related to Figure 1.

Sample ID in GEO(GSE38233)	Sample name in GEO	Individual	Experiment	Library insert size	Sequencing	Average Read length	Total # of reads	#aligned reads	# uniquely aligned reads	% of aligned reads	% of unquely aligned reads
Sample 1	Sample_GM12750_baseline	GM12750	baseline	200-350 bp	Single-end	97 nt	142,255,567	135,660,583	112,735,386	95.4%	79.2%
Sample 2	Sample_GM12004_baseline	GM12004	baseline	200-350 bp	Single-end	98 nt	175,654,256	171,103,388	139,411,120	97.4%	79.4%
Sample 3	Sample_GM12750_Directional	GM12750	baseline, directional	180-200 bp	Single-end	97 nt	206,154,747	172,813,413	139,340,950	83.8%	67.6%
Sample 4	Sample_GM12004_Directional	GM12004	baseline, directional	180-200 bp	Single-end	97 nt	201,366,748	170,030,478	135,316,699	84.4%	67.2%
Sample 5	Sample_GM12750_Directional_ADAR_KD	GM12750	ADAR1-siRNA, directional	180-200 bp	Single-end	96 nt	186,855,107	136,709,952	111,042,298	73.2%	59.4%
Sample 6	Sample_GM12750_Directional_NT	GM12750	NTC siRNA, directional	180-200 bp	Single-end	95 nt	149,184,629	80,370,194	64,979,507	53.9%	43.6%
Sample 7	Sample_GM12750_NT	GM12750	NTC siRNA	200-350 bp	Single-end	97 nt	82,666,415	80,465,767	67,257,829	97.3%	81.4%
Sample 8	Sample_GM12750_ADAR_KD	GM12750	ADAR1 siRNA	200-350 bp	Single-end	96 nt	114,729,241	111,689,921	93,568,018	97.4%	81.6%
Sample 9	Sample_GM12004_NT	GM12004	NTC siRNA	200-350 bp	Single-end	98 nt	188,841,363	183,119,621	150,857,634	97.0%	79.9%
Sample 10	Sample_GM12004_ADAR_KD	GM12004	ADAR1 siRNA	200-350 bp	Single-end	98 nt	196,982,179	191,523,523	156,668,557	97.2%	79.5%
Sample 11	Sample_GM12004_ADAR2_kd	GM12004	ADAR2 siRNA	200-350 bp	Single-end	98 nt	188,282,790	183,311,559	152,896,261	97.4%	81.2%
Sample 12	Sample_GM12750_ADARkd_ATime	GM12750	ADAR1-siRNA, time point 24 hour	200-350 bp	Single-end	98 nt	171,412,869	166,839,294	141,015,077	97.3%	82.3%
Sample 13	Sample_GM12750_ADARkd_BTime	GM12750	ADAR1-siRNA, time point 48 hour	200-350 bp	Single-end	98 nt	192,108,845	186,522,411	156,761,432	97.1%	81.6%
Sample 14	Sample_GM12750_ADARkd_CTime	GM12750	ADAR1-siRNA, time point 72 hour	200-350 bp	Single-end	98 nt	192,043,296	186,020,035	152,778,114	96.9%	79.6%
Sample 15	Sample_GM12750_ADARkd_DTime	GM12750	ADAR1-siRNA, time point 96 hour	200-350 bp	Single-end	98 nt	188,777,483	182,741,246	149,791,979	96.8%	79.3%
Sample 16	Sample_GM12750_nt_Dtime	GM12750	NTC siRNA, time point 96 hour	200-350 bp	Single-end	98 nt	185,192,178	177,125,378	146,721,291	95.6%	79.2%
Sample 17	Sample_RNA-IP_ADAR_GM12004	GM12004	ADAR1 RNA-IP	200-350 bp	Single-end	98 nt	186,593,821	119,606,085	91,717,555	64.1%	49.2%
Sample 18	Sample_RNA-IP_IgG_GM12004	GM12004	control IgG RNA-IP	200-350 bp	Single-end	98 nt	204,394,300	130,890,544	98,581,153	64.0%	48.2%
Sample 19	Sample_RNA-IP_ADAR_KD_GM12750	GM12750	ADAR1 RNA-IP	200-350 bp	Single-end	98 nt	213,942,446	145,198,134	110,025,519	67.9%	51.4%
Sample 20	Sample_RNA-IP_IgG_GM12750	GM12750	control IgG RNA-IP	200-350 bp	Single-end	98 nt	215,323,408	146,778,885	111,075,019	68.2%	51.6%

Table S2 RNA-DNA differences identified from analysis of RNA-seq and DNA-seq of B-cells from two individuals, related to Figure 1.

Table S3 ADAR1 knockdown leads to changes in editing levels of target genes, related to Figure 3.

Table S4 ADAR2 knockdown leads to changes in editing levels of target genes, related to Figure 4.

Table S5 Editing sites identified from anti-ADAR1 RNA-immunoprecipitation experiment, related to Figure 4.

Table S6 Genes whose expression levels changed significantly following *ADAR1* knockdown in both individuals, related to Figure 3.

Table S7. Gene ontology analysis of genes whose expression levels changed following *ADAR1* knockdown, related to Figure 3.

Gene Ontology Term	# Gene	P-Value	Examples
Cell cycle	111	$8.5X10^{-18}$	*JUB, AURKA, SEPT8, CENPE, CDC25C, CDKN3*
ATP binding	133	$6.6X10^{-11}$	*MCM4, ATP8A1, KIFC1, ZAK, DTYMK, DSTYK*
Kinase	81	$5.4X10^{-10}$	*ZAK, PHKB, DTYMK, DSTYK, TTK, AURKA*
DNA damage response	57	$2.2X10^{-10}$	*MLH1, NEIL1, RAD52, RAD51, ATRIP, EXO1*
Chromosome segregation	22	$8.8X10^{-9}$	*NEK2, CENPE, BIRC5, SMC2, SMC4, CENPH*
Ubiquitin-like protein conjugation	64	$7.3X10^{-7}$	*UBE2C, UBE2S, USP1, ATG10, RNF14, USP1*
Zinc-finger	143	$1.8X10^{-6}$	*THRA, PLEKHM1, ZNF580, ZNF451, MCM10, ZNF251*
Chromatin	24	0.0004	*TMPO, HMGN1, HIST1H2AC, HMGN2, ARID4B, HDAC2*

Table S8. Genes whose expression levels and editing levels both changed at least 20% following *ADAR1* knockdown, related to Figure 5.

Gene Symbol	# Edited sites	Type	Chromosome	Average change in editing level (%)	Average change in FPKM (%)
AMD1	4	A>G	6	-99	-60
ANKDD1A	1	A>G	15	-100	30
AP3S2	3	A>G	15	-86	60
APC2	4	A>G	19	-47	60
APOL1	13	A>G	22	-76	-30
ARRDC2	1	A>G	19	-12	90
ATG12	3	A>G	5	-86	50
ATP5S	30	A>G	14	-86	40
AURKA	1	A>G	20	-93	-40
B4GALT2	1	A>G	1	-16	-40
BLCAP	1	A>G	20	-52	-70
C14orf118	5	A>G	14	-76	40
C15orf23	1	A>G	15	-66	60
C15orf40	3	A>G	15	-45	70
C16orf5	1	A>G	16	-100	70
C17orf85	1	A>G	17	-65	90
C19orf12	1	A>G	19	-100	30
CALR3	1	A>G	19	-13	-40
CARD8	7	A>G	19	-88	270
CBFA2T2	3	A>G	20	-100	-60
CBFB	1	A>G	16	-100	-20
CBX5	2	A>G	12	-86	50
CCDC84	6	A>G	11	-96	30
CD302	1	A>G	2	-100	50
CENPN	35	A>G	16	-78	-50
CFLAR	9	A>G	2	-86	-30
CHST12	1	A>G	7	-84	-50
CLTA	4	A>G	9	-42	-40
CPNE1	4	A>G	20	-100	40
CRCP	32	A>G	7	-71	70
CTH	4	A>G	1	-97	-30
DAP3	1	A>G	1	-100	90
DCLRE1C	3	A>G	10	-73	40
DCTN5	5	A>G	16	-76	-30
DHFRL1	2	A>G	3	-96	-50
DMTF1	1	A>G	7	-82	-30
ECHDC2	1	A>G	1	-100	30
EEF1D	1	A>G	8	9	120
EGR1	1	A>G	5	100	30
EIF2AK2	54	A>G	2	-87	120
EIF3L	2	A>G	22	-100	180
EPB41	1	A>G	1	-1	-70
FAM184B	1	A>G	4	-100	20
FAM55C	5	A>G	3	-78	50
FCER2	24	A>G	19	-86	30
FDPS	2	A>G	1	-70	40
FLNB	1	A>G	3	-88	-50
GSDMB	2	A>G	17	-93	-40
GSR	13	A>G	8	-91	-60
HAUS2	24	A>G	15	-89	-50
HM13	2	A>G	20	-100	60
IFNAR2	15	A>G	21	-84	-50
IKZF3	62	A>G	17	-83	40
IL15	1	A>G	4	-100	-30
ILDR1	1	A>G	3	-100	40
IQCB1	2	A>G	3	-72	-30

Gene Symbol	# Edited sites	Type	Chromosome	Average change in editing level (%)	Average change in FPKM (%)
LYRM2	3	A>G	6	-97	-30
MAVS	69	A>G	20	-70	330
MCART1	3	A>G	9	-90	-30
MCM4	5	A>G	8	-94	-40
MDM4	9	A>G	1	-50	30
NARF	3	A>G	17	-94	190
NASP	1	A>G	1	-45	-40
NDUFS1	31	A>G	2	-86	230
NIF3L1	1	A>G	2	-25	250
PAIP1	3	A>G	5	-47	60
PARVB	2	A>G	22	-73	130
PARVG	5	A>G	22	-86	-60
PEX26	17	A>G	22	-86	-30
PHF7	2	A>G	3	-91	-60
PILRA	1	A>G	7	-85	40
PML	2	A>G	15	-18	259770
PPHLN1	4	A>G	12	-66	340
PPP2R1B	4	A>G	11	-90	-60
PRKRA	27	A>G	2	-83	100
PUS1	2	A>G	12	-100	-60
RASSF1	1	A>G	3	-100	-40
RBBP4	2	A>G	1	-66	14510
RCC1	6	A>G	1	-85	30
RFFL	1	A>G	17	-94	30
RPL28	3	A>G	19	-67	50
RPL36A	2	A>G	X	-50	30
RSRC2	1	A>G	12	-100	70
SERBP1	5	A>G	1	-96	90
SH3BP2	6	A>G	4	-43	-30
SLC3A2	1	A>G	11	-34	70
SNX1	1	A>G	15	-69	60
TAPBP	1	A>G	6	-73	30
TCP11L1	7	A>G	11	-84	-40
TFDP2	12	A>G	3	-74	30
TGOLN2	2	A>G	2	-63	9490
TMEM116	2	A>G	12	0	-40
TMPO	16	A>G	12	-85	-30
TRIM5	3	A>G	11	-100	120
TRIM66	1	A>G	11	-38	30
UBE2H	1	A>G	7	-66	70
VNN2	1	A>G	6	-100	170
XIAP	42	A>G	X	-87	180
ZNF276	2	A>G	16	11	60
ZNF397	5	A>G	18	-97	30
ZNF573	1	A>G	19	-100	110
ZNF611	5	A>G	19	-86	60
ZNF615	2	A>G	19	-61	20
ZNF621	7	A>G	3	-52	-40
ZNF841	4	A>G	19	-86	30
ZNF844	3	A>G	19	-100	30

Table S9 Genes whose expression levels changed at least 2 fold following ADAR2 knockdown, related to Figure 4.

Table 10. Primers used in this study, related to experimental procedures.

**Primers using in Sanger Sequencing**						
**Chr**	**Position**	**Gene Symbol**	**Forward primer (external)**	**Reverse primer (external)**	**Forward primer (internal)**	**Reverse primer (internal)**
11	107741679 - 107741851	ATM	N/A	N/A	5'-CATACAGCAGGCCATAGACC-3'	5'-GGCCCACAGCAACCTTCACCTCCCAG-3'
6	90487981	MDN1	N/A	N/A	5'-TGGCTTGACTACCCTGTGTG-3'	5'-CATGCTTGCTTGTTGTGGTC-3'
7	89857293, 89857303	GTPBP10	5'-ACAGTGGACAAAAAGTGTTGGCAGA-3'	5'-AGCTGGCTACTGACACCCACTCA-3'	5'-CAGGGTCTCT CTGTGGAGTG-3'	5'-CTAATACATTTCCCTCTGCT-3'
9	32446365, 32446368,32446371	DDX58	N/A	N/A	5'-TGGCTACACAGAGAACATGAGAA-3'	5'-GAGAACATATTAATAGGGCAAGATG-3'
10	74678737	MRPS16	5'-GGGAGCTTTGACAACCACAG-3'	5'-AAGATACCATTGGGGCAGTG-3'	5'-CTAGCCAAATTATGTAATGT-3'	5'-ACAGCAAGACCCCGTCTCTA-3'
10	114199337, 114199343	VTI1A	5'-TCCACTGCCGGAGATCGTCTTGA-3'	5'-TGAAGCTCCTGTGTTTGGGCCT-3'	5'-TGAGGTCGGGAGTTTGAGAC-3'	5'-AATCTCAGCTCATCGCAACC-3'
14	19905006	TEP1	N/A	N/A	5'-CCCGGCCCATAAAAGAACT-3'	5'-AAAGAAAGGGCCTGAGGAAA-3'
14	52179482, 52179560	ERO1L	5'-GGAAAAATTTGTGTGTGTGTGTG-3'	5'-CTTTAATAATGTGGTACAAAT-3'	5'-TAGAAATCTCCTGTGCCTTT-3'	5'-AAAATGCTAAGGAGGCCTCA-3'
**Primers and oligos used for droplet digital PCR**						
**Chr**	**Position**	**Gene Symbol**	**Forward primer (external)**	**Reverse primer (external)**	**VIC probe**	**FAM probe**
1	159227224	F11R	5'-TATTTGGAAATCCCTAACAGAATTGAGTTT-3'	5'-CCTTGACTGATGGCTTCATTAGCAT-3'	5'-TTCTTTTTGGATCCTTAATAGA-3'	5'-CTTTTTGGATCCCTAATAGA-3'
2	20314300	PUM2	5'-AACAGATTAACAATCAACTGCATAAATATT-3'	5'-CTCGTTATTTGCATGATAGTTTGTGAATT-3'	5'-ATCAAAATACAACTTAACTCTT-3'	5'-AAAATACAACTCAACTCTT-3'
3	151819253	SELT	5'-ACTCCTGGGATCAAGTGAACCT-3'	5'-TCACATCTGTAATCCCAGCACTTTG-3'	5'-CTGCCTCAGCCTCC-3'	5'-TGCCTCGGCCTCC-3'
5	49728037	EMB	5'-GAGAGACCAACAAATGTATATTTATAACACAGAGT-3'	5'-CCCATACCTGGTAGAGCATGTAC-3'	5'-AACTCCACATTTATTTGTG-3'	5'-CTCCACATTTGTTTGTG-3'
5	67632909	PIK3R1	5'-TCCAACTTAACATGAAACTTGTCACCAT-3'	5'-ACACACACACACACACACACATATA-3'	5'-AGATAGCATTAGCTGCCC-3'	5'-ATAGCATTGGCTGCCC-3'
21	33845189	SON	5'-GCAGCCTGTGGCAACTG-3'	5'-GCCCCAGCTGCCATGA-3'	5'-CAGGCAACTCTAGTGCC-3'	5'-AGGCAACTCCAGTGCC-3'

**Primers used for RNA pull-down experiments**		
**Gene Symbol**	**Forward primer (with T7 promoter)**	**Reverse primer**
MCM4	5'-TAATACGACTCACTATAGGAGCCTTGTGAGCAAGGAAGGCTCCC-3'	5'-TTAAAGGTTTCAGAAATTATTTAT-3'
GSR	5'-TAATACGACTCACTATAGGCTTGAGCCCAGGAGTTCAAGAC-3'	5'-CTTTTCCCAGGTGGTTGGGAT-3'
CTH	5'-TAATACGACTCACTATAGGTATTCCAGAGCTGCTATTAGAAGCT-3'	5'-CCTCAGCCTCCCAAGTAGCTGGGAC-3'

Extended Experimental Procedures

Cell culture. Cultured B-cell lines from two CEPH individuals (Centre d'Etude du Polymorphisme Humain), GM12004 and GM12750, were obtained from Coriell Cell Repositories (Camden, NJ, USA). The B-cells were grown to a density of $5x10^5$ cells/mL in RPMI 1640 supplemented with 15% fetal bovine serum, 100 units/mL penicillin and 100 μg/mL streptomycin, and 2 mmol/L L-glutamine. For downstream experiments, cells were harvested 24 hours after addition of fresh medium.

siRNA knockdown and quantitative RT-PCR. $5X10^5$ cultured B cells were transfected with 2.5 nmol Accell siRNAs (Thermo Scientific) against ADAR1 and ADAR2 according to the manufacturer's protocols. 2.5 nmol of non-targeting control siRNA (NTC-siRNA) was transfected as negative controls. The cells transfected with siRNAs were incubated at 37°C in Accell transfection media for 96 hours. In time course experiment, cells were treated with another 2.5 nmol of siRNAs 24 hours following the first treatment. Accell media was then replaced with regular growth media. Cells were allowed to recover for 24 hours before being harvested. DNA and RNA were extracted using Qiagen DNeasy blood and tissue kit and RNeasy Mini kit with DNase treatment, respectively (Qiagen). Reverse transcription was carried out using Taqman reverse transcription reagent kit (Applied Biosystems). Gene expression was analyzed by quantitative PCR on ABI 7900HT system following manufacturer's protocol (Applied Biosystems).

Off-target effects of siRNA knockdown. In order to assess the off-target effects of siRNA knockdown, we transfected B-cells with four individual siRNA targeting different regions of *ADAR1* or pooled siRNA. Cells were harvested 96 hours post transfection, and real time RT-PCR and western blot were carried out to measure changes of *ADAR1*

at mRNA and protein levels. We found the pooled and individual siRNA reduced expression of ADAR1 similarly (Figure S2). In addition, pooling of siRNA is known to reduce off-target effects(Grimson et al., 2007). Therefore we used pooled siRNA throughout the study.

Next-generation sequencing and data analysis. Genomic DNA was extracted from cultured B-cells of GM12004 and GM12750 using DNeasy blood and tissue kit (Qiagen, Valencia, CA, USA). DNA-seq libraries were prepared and sequenced on HiSeq 2000 instrument (Illumina, San Diego, USA). Paired-end 100-nt reads were generated in order to achieve 60X and 30X coverage for GM12004 and GM12750, respectively. DNA-seq data were aligned to human genome (hg18) using CASAVA and BAM files generated. To select sites for further consideration: we identified those that 1) are covered by 10 or more reads, 2) have only one type of nucleotide in all reads (homozygous sites).

For transcriptome sequencing, total RNA was extracted using the RNeasy Mini kit with DNase treatment (Qiagen). RNA-seq libraries were prepared following Illumina TruSeq RNA sample preparation protocol or directional mRNA-Seq sample preparation protocol, respectively. The samples were sequenced using HiSeq 2000 instrument and 100-200 million 100-nt reads per sample were generated. Low quality bases at the 3' ends of reads defined by Illumina were trimmed, and the resulting reads were aligned to the human reference genome (hg18) using GSNAP (version 2011-03-28.v3) (Wu and Nacu, 2010) using the following parameters: Mismatches ≤[(read length+2)/12-2]; Mapping score ≥20; Soft-clipping on (-trim-mismatch-score=-3); Known exon-exon junctions (defined by RefSeq (downloaded March 7, 2011) and Gencode (version 3c))

and novel junctions (defined by GSNAP) were used. SNP sites in the CEU population from Hapmap (release #28) and 1000 Genomes (pilot project) were included for SNP-tolerant alignments. Only reads that aligned to one genomic location (uniquely mapped reads) were used in further analyses.

Expression levels of RNA transcripts were analyzed using Cufflinks version 2.1.1. using default prarmeters (Trapnell et al., 2010). We require RPKM>0.1 in at least 3 out of 4 samples (ADAR1-siRNA or negative control siRNA in B-cells from two individuals). Gene ontology analysis was carried out on DAVID Bioinformatics Resources 6.7 platform, based on a modified fisher exact test (Huang et al., 2009a, 2009b)

RNA-DNA differences. To identify RDDs, we compared RNA sequence to its corresponding DNA sequence. To be included as RDD sites in the final lists, the following criteria have to be met: 1) a minimum of 10 total RNA-seq reads covering that site in both individuals; 2) a minimum of 10 total DNA-seq reads covering that site in both individuals; 3) DNA sequence at this site is 100% concordant, without any DNA-seq reads containing alternate alleles; 4) level of RDD (# of RNA-seq reads containing non-DNA allele/# all RNA-seq reads covering a given site) is ≥10% (a minimum of two RNA-seq reads containing RDD) in at least one individual; 4) the RDD site is covered by stranded RNA-seq reads in directional RNA sequencing data, which defines strand specificity of RDD at this site; 5) RDD event is found in both individuals.

To ensure the accuracy of the RDD sites, additional filtering steps were performed using two additional mapping algorithms. First, we removed all the sites that reside in genome regions annotated as “pseudogene” in RefSeq. Second, local sequences around each

RDD site were aligned to the human reference genome to rule out misalignments to paralogous sequences or remaining pseudogenes. Specifically, for each RDD event, genomic sequences comprising sequences of length 25 bp, 50 bp, and 75 bp upstream and downstream of each site along with either the DNA variant or RNA variant were aligned to the human reference genome (hg18) using BLAT (v. 34x11). The settings '-stepSize=5' and 'repMatch=2253' were used to increase sensitivity. RDD events were removed if any of the 6 corresponding sequences aligned to another genomic location with ≤3 mismatches and with sequences that explain the RDD call (that is, if the genomic sequences match the RNA sequence). Lastly, to avoid potential misalignment of spliced reads in GSNAP due to its high gap penalty algorithm, we re-aligned all the RNA-seq reads that contain putative RDD alleles using BLAT. Human genome sequences in hg19 were included in our index in addition to sequences in hg18. Here, a low gap penalty was applied during BLAT alignment in order to compensate for high gap penalty of GSNAP alignment of spliced reads. Only RDD sites that are supported by both GSNAP and BLAT are retained for downstream analysis.

Validation of RDD using Sanger Sequencing and droplet digital PCR. To validate RDD using Sanger Sequencing, sequences surrounding RDD sites were PCR amplified using genomic DNA or cDNA as template, respectively. PCR products were sequenced using BigDye Terminator Cycle Sequencing on a 3730 DNA Analyzer (Applied Biosystems) and the results were analyzed by visual inspection. The 3'UTR region of ATM was amplified from cDNA of B-cells and cloned into TOPO vector (Invitrogen). Individual clones were sequenced, and 137 clones provided informative sequence information for downstream analysis. For sites within repetitive Alu regions, we carried

out nested-PCR with external primer annealing to non-repetitive sequences. Primers used for PCR were listed in Table S10.

For droplet digital PCR, DNA probes specific to the DNA and RNA variants at RDD sites were synthesized and labeled by VIC and FAM, respectively (ABI Biosystems, USA). PCR reaction was prepared using genomic DNA or cDNA from our subjects, VIC- and FAM- probes and Taqman reagents. Emulsion PCR was carried out following manufacture's protocol (Bio-Rad Laboratories, USA). Fluorescent signal representing each variant was quantified utilizing QuantaLife Droplet Reader (Bio-Rad Laboratories, USA). Primers and probes used in this assay are listed in Table S10.

Immunoprecipitation and western blot. Cells were lysed in Lysis buffer (20 mM Tris HCl (pH 8), 137 mM NaCl, 10% glycerol, 1% Nonidet P-40 (NP-40) and 2 mM EDTA) supplemented with 1XComplete protease inhibitors (Roche) and 1Xphosphatase inhibitors II and III (Sigma). Cell lysates containing 150 mg of total protein were incubated with 1 µg of anti-ADAR1 (#HPA003890, Sigma), or negative control rabbit IgG (ab46540, ABCAM) at 4°C overnight. Immuno-complex was pulled down using Protein A agarose (Roche), and washed 4 times with lysis buffer. Immunoprecipitate was eluted in 20 mM Tris/7.5, 150 mM NaCl, 2.5 mM MgCl2, 0.2% SDS at 30°C for 1 hour. In order to examine RNA-dependent interactions, whole cell lysates were diluted to 1 ug/ul, RNase A (Epicenter) was added to a final concentration of 0.1 ug/ul or RNase V1 (Ambion) was added to a final concentration of 0.002U/ul, and lysates were incubated at room temperature for 15 minutes. Protein samples were analyzed by western blot using anti-ADAR antibody or anti-tubulin (#05-661, Millipore), anti-EIF2AK2 (#sc-136038, Santa Cruz), anti-ILF3 (#SAB1406034, Sigma), anti-HuR (#ab54987,

ABCAM) antibodies. RNA pull-down experiment was carried out following manufactures' manual (Pierce, #20164). Primers used for amplifying DNA regions for *in vitro* synthesis of RNA transcripts are listed in Table S10.

Immunofluoresence. Primary fibroblasts were cultured at 37°C, 5% CO_2 on 1.5-thickness glass coverslips in 6-well culture plates to ~40% confluence. Cells were washed in 1xPBS and fixed in 4% paraformaldehyde in PBS for 15 minutes at room temperature. Cells were permeabilized and blocked in blocking buffer (1xPBS, 5% normal goat serum, and 0.3% Triton X-100) with gentle shaking for one hour at room temperature. Cells were then incubated with primary antibodies in antibody dilution buffer (1xPBS, 10% bovine serum albumin, 0.3% Triton X-100) at 4°C with gentle shaking overnight. Mouse anti-ADAR antibody (#ab88574, Abcam) was diluted to 10ug/ml and rabbit anti-EIF2AK2 (#3073, Cell Signaling) was diluted to 5ug/ml. Following overnight incubation, cells were washed in 1xPBS and incubated with secondary antibodies for 2 hours at room temperature with gentle shaking. The secondary antibody cocktail comprised of 2ug/ml of anti-mouse IgG with Alexa Fluor 488 conjugate (#4408, Cell Signaling) and 2ug/ml of anti-rabbit IgG with Alexa Fluor 555 conjugate (#4413, Cell Signaling). Cells were then immersed in 10ug/ml of Hoechst 33342 (#H3570, Life Technologies) in water for 30 seconds for nuclear staining. Cells were washed in 1xPBS, mounted with 12ul of VECTAshield mounting medium (#H-1000, Vector Labs), and affixed to glass microscope slides with clear nail polish. Standard epifluorescence visualization was performed on a Leica Microsystems DMI6000B with a DFC360FX camera using an HCX PL APO 40X / 0.75 Dry objective with a 1.6X magnification changer and analyzed with Leica Microsystems LAS AF6000

software. Confocal fluorescence visualization was performed on a Zeiss LSM 710 AxioObserver using a Plan-Apochromat 63X/1.4 Oil DIC M27 objective and analyzed with ZEN lite 2011 software. Shown is [z-position 7 out of 15] or [orthogonal view].

RNA-immunoprecipitation. Anti-ADAR1 and anti-HuR RNA-immunoprecipitation was carried out using Magna RNA-Binding Protein Immunoprecipitation Kit (Millipore) following manufacturer's protocol. Briefly, for each immunoprecipitation reaction, $2X10^7$ cultured B-cells were harvested and lysed in 100 µl of Lysis Buffer with protease and RNase inhibitors. 1 µg of anti-ADAR antibody (#HPA003890, Sigma), anti-HuR antibody (#sc-20694 AC, Santa Cruz), or negative control rabbit IgG (ab46540, ABCAM) were conjugated to Protein A/G beads. 100 µl of cell lysate was added into 900µl Immunoprecipitation Buffer with RNase inhibitor and incubated with 50 µl beads-antibody complex at 4°C overnight. Beads-bound immunoprecipitates were then washed six times using cold Wash Buffer with RNase inhibitor, and incubated with protease K in presence of 1% SDS at 55°C for 30 minutes. RNA was then extracted from supernatants using phenol:chloroform:isoamyl alcohol and precipitated using ethanol, followed by DNase digestion (DNA-free kit, Ambion). cDNA was synthesized using random hexamer primer by Taqman Reverse Transcription Reagent kit (Applied Biosystems). PCR and RNA-seq were carried out as described above. RNA-editing sites that were detected in transcripts pulled down by ADAR1 antibody but not by negative control IgG were considered as ADAR1-specific targets and retained for further analysis.

Overlap between editing sites identified in this and previous studies

In this study, we identified 64,931 A-to-G editing sites, from mRNA and other RNA species. We compared our results to the editing sites catalogued in the DARNED database and to those from recent studies (Bahn et al., 2011; Carmi et al., 2011; Kiran and Baranov, 2010; Li et al., 2009; Peng et al., 2012). There are 7,452 sites (11.5% of all editing sites identified in this study) that overlap between this and other studies (Figure S4B). Fifty-seven percent of the overlaps are those between the editing sites identified here and the study by Peng and colleagues. The larger overlap between these two studies is likely because the same cell type (cultured B-cells) was used for analyses. Since most of our sites were identified from analyzing products of ADAR RNA-IP which include different types of RNA (including immature and unprocessed RNA) whereas other studies mostly focused on processed mRNA, the small overlap between our and other studies is not unexpected. In addition to comparing the editing sites, we compared the genes that were edited and found that 3,306 genes (24.8% of all edited genes identified in this study) overlap between ours and previous studies. Others also found only modest overlap of editing sites among studies (Osenberg et al., 2009). This trend suggests that there are many editing sites and for most sites the editing levels are low; thus, each study samples only a different subset of these sites. A comprehensive catalogue of editing sites will require very deep nucleic acid sequencing.

Supplemental References

Bahn, J.H., Lee, J.-H., Li, G., Greer, C., Peng, G., and Xiao, X. (2011). Accurate identification of A-to-I RNA editing in human by transcriptome sequencing. Genome Research.

Carmi, S., Borukhov, I., and Levanon, E.Y. (2011). Identification of Widespread Ultra-Edited Human RNAs. PLoS Genet *7*, e1002317.

Grimson, A., Farh, K.K.-H., Johnston, W.K., Garrett-Engele, P., Lim, L.P., and Bartel, D.P. (2007). MicroRNA targeting specificity in mammals: determinants beyond seed pairing. Mol. Cell *27*, 91–105.

Huang, D.W., Sherman, B.T., and Lempicki, R.A. (2009a). Systematic and integrative analysis of large gene lists using DAVID bioinformatics resources. Nat Protoc *4*, 44–57.

Huang, D.W., Sherman, B.T., and Lempicki, R.A. (2009b). Bioinformatics enrichment tools: paths toward the comprehensive functional analysis of large gene lists. Nucleic Acids Res. *37*, 1–13.

Kiran, A., and Baranov, P.V. (2010). DARNED: a DAtabase of RNa EDiting in humans. Bioinformatics *26*, 1772 –1776.

Li, J.B., Levanon, E.Y., Yoon, J.-K., Aach, J., Xie, B., Leproust, E., Zhang, K., Gao, Y., and Church, G.M. (2009). Genome-wide identification of human RNA editing sites by parallel DNA capturing and sequencing. Science *324*, 1210–1213.

Osenberg, S., Dominissini, D., Rechavi, G., and Eisenberg, E. (2009). Widespread cleavage of A-to-I hyperediting substrates. RNA *15*, 1632–1639.

Peng, Z., Cheng, Y., Tan, B.C.-M., Kang, L., Tian, Z., Zhu, Y., Zhang, W., Liang, Y., Hu, X., Tan, X., et al. (2012). Comprehensive analysis of RNA-Seq data reveals extensive RNA editing in a human transcriptome. Nature Biotechnology.

Trapnell, C., Williams, B.A., Pertea, G., Mortazavi, A., Kwan, G., van Baren, M.J., Salzberg, S.L., Wold, B.J., and Pachter, L. (2010). Transcript assembly and quantification by RNA-Seq reveals unannotated transcripts and isoform switching during cell differentiation. Nature Biotechnology *28*, 511–515.

Wu, T.D., and Nacu, S. (2010). Fast and SNP-tolerant detection of complex variants and splicing in short reads. Bioinformatics *26*, 873–881.
